# Human-Centered Design of a Digital Health Tool to Promote Effective Self-care in Patients With Heart Failure: Mixed Methods Study

**DOI:** 10.2196/34257

**Published:** 2022-05-10

**Authors:** William Johnston, Alison Keogh, Jane Dickson, Stephen J Leslie, Peter Megyesi, Rachelle Connolly, David Burke, Brian Caulfield

**Affiliations:** 1 Insight Centre for Data Analytics University College Dublin Dublin Ireland; 2 School of Public Health, Physiotherapy & Sports Science University College Dublin Dublin Ireland; 3 Physiotherapy Department Beacon Hospital Dublin Ireland; 4 Cardiac Unit Raigmore Hospital Inverness United Kingdom; 5 Cardiology Beacon Hospital Dublin Ireland; 6 School of Medicine University College Dublin Dublin Ireland

**Keywords:** digital health, heart failure, cardiology, self-care, behavior change, eHealth, mHealth, mobile health, mobile app, mobile phone

## Abstract

**Background:**

Effective self-care is an important factor in the successful management of patients with heart failure (HF). Despite the importance of self-care, most patients with HF are not adequately taught the wide range of skills required to become proficient in self-care. Digital health technology (DHT) may provide a novel solution to support patients at home in effective self-care, with the view to enhancing the quality of life and ultimately improving patient outcomes. However, many of the solutions developed to date have failed to consider users’ perspectives at the point of design, resulting in poor effectiveness. Leveraging a human-centered design (HCD) approach to the development of DHTs may lead to the successful promotion of self-care behaviors in patients with HF.

**Objective:**

This study aimed to outline the HCD, development, and evaluation process of a DHT designed to promote effective self-care in patients with HF.

**Methods:**

A design thinking approach within the HCD framework was undertaken, as described in the International Organization for Standardization 9241-210:2019 regulations, using a 5-step process: empathize, ideate, design, develop, and test. Patients with HF were involved throughout the design and evaluation of the system. The designed system was grounded in behavior change theory using the Theoretical Domains Framework and included behavior change techniques. Mixed methods were used to evaluate the DHT during the testing phase.

**Results:**

Steps 1 to 3 of the process resulted in a set of evidence- and user-informed design requirements that were carried forward into the iterative development of a version 1 system. A cross-platform (iOS and Android) mobile app integrated with Fitbit activity trackers and smart scales was developed. A 2-week user testing phase highlighted the ease of use of the system, with patients demonstrating excellent adherence. Qualitative analysis of semistructured interviews identified the early potential for the system to positively influence self-care. Specifically, users perceived that the system increased their confidence and motivation to engage in key self-care behaviors, provided them with skills and knowledge that made them more aware of the importance of self-care behaviors, and might facilitate timely help seeking.

**Conclusions:**

The use of an HCD methodology in this research has resulted in the development of a DHT that may engage patients with HF and potentially affect their self-care behaviors. This comprehensive work lays the groundwork for further development and evaluation of this solution before its implementation in health care systems. A detailed description of the HCD process used in this research will help guide the development and evaluation of future DHTs across a range of disease use cases.

## Introduction

Heart failure (HF) is a significant global public health problem, with a prevalence of >100 per 1000 people aged ≥65 years [1]. As HF progresses, it is associated with significant morbidity and mortality, resulting in an increased number of hospitalizations. In addition to the impact that deteriorating health has on the patient’s quality of life, these hospitalizations place a significant burden on health services, representing 80% of the costs associated with HF care [2].

Self-care centers have autonomy, independence, and a person’s responsibility for healthy behaviors and the development of activities required to manage and monitor health conditions [3]. It is well-accepted that promoting effective self-care and disease management in patients with HF is central to reducing the burden on patients and the health system [3-5]. Despite this, patients often struggle to develop the wide range of skills required to become proficient in HF self-care [6]. For example, it is suggested that patients should be able to manage fluid retention through intake and weight monitoring; adhere to diet, medication, and exercise regimens; monitor symptoms; recognize deterioration; and ultimately, identify when they need to engage in help-seeking behaviors [3,6]. However, research has shown that most patients with HF do not understand the nature of their HF, cannot link changes in symptoms to their condition, and do not engage in these key self-care behaviors on a regular basis, suggesting that current educational practices around self-care are not effective or sufficient to elicit lasting change [6].

Over the past 20 years, consumer-wearable and mobile technologies have become ubiquitous. These technologies provide an opportunity to develop new approaches to empower patients to engage with and manage their chronic conditions at home and connect them to their care team in a timely manner when most required [7,8]. However, although existing solutions have demonstrated some success, their effectiveness has been relatively inconsistent [9-11]. One of the key reasons put forward for this lack of success is the failure of these solutions to integrate the users’ perspective at the point of design, thus neglecting to take into account the patients’ actual needs and failing to truly understand the problems to be solved [12-14]. Recently, the use of human-centered design (HCD) approaches has led to the successful development of digital health technologies (DHTs) designed to support chronic disease management [15]. As such, leveraging an HCD approach to the development of DHTs designed to promote self-care behaviors in patients with HF may positively influence the impact their condition has on their quality of life.

Therefore, the aim of this study was to use an HCD approach to design and develop a DHT to promote effective self-care behaviors in patients with HF. This paper provides a detailed step-by-step description of the HCD process followed in this research, detailing the process from initial requirement gathering and conceptual design to user testing.

## Methods

### Design Approach

**Figure 1:**
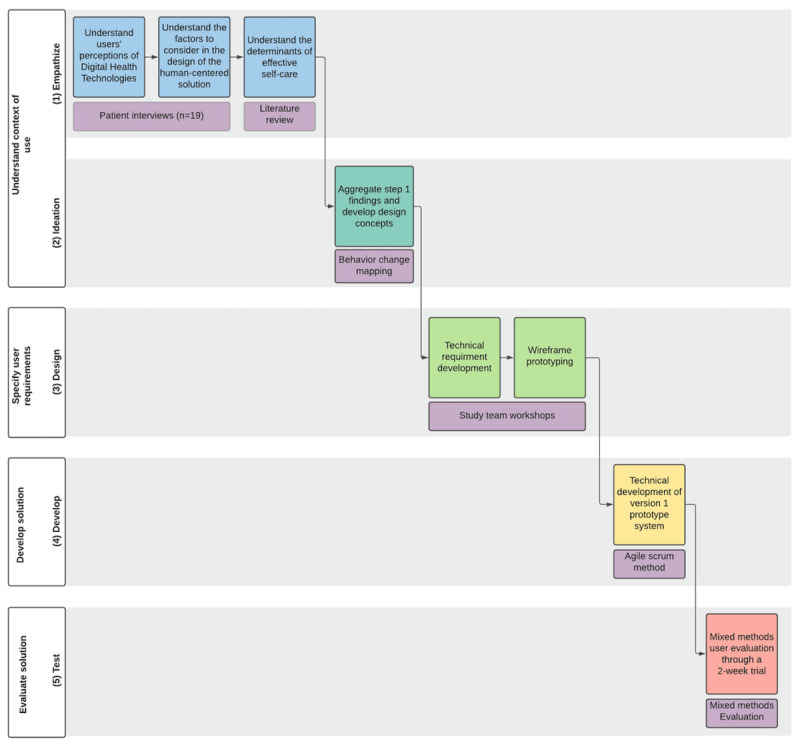
The multistep human-centered design process.

This study leveraged a design thinking approach within the HCD framework, as described in the International Organization for Standardization 9241-210:2019 regulations [16]. This methodology follows a five-step iterative process: (1) empathize, (2) ideate, (3) design, (4) development, and (5) test (Figure 1). The entire design and development process was conducted between 2019 and 2021. The methods used in each phase are detailed sequentially in the *Methods* and *Results* sections.

### Step 1: Empathize

#### Overview

The aim of step 1 was three-fold: (1) to understand the perceptions of patients with HF on the use of DHTs for the management of their condition, (2) to understand the factors to be considered in the design and implementation of DHTs, and (3) to understand the key determinants of effective self-care in patients with HF. To address the first 2 aspects, a cohort of patients with HF was recruited to participate in semistructured interview sessions (study 1) [17]. To further understand the key determinants of effective self-care in patients with HF, the previously published systematic review of patient and caregiver perceptions conducted by Clark et al [6] was consulted, alongside international best practice statements on HF self-care promotion by the American Heart Association [4] and the European Society of Cardiology [3].

#### Semistructured Interviews (Study 1)

##### Participants

A total of 19 participants (n=6 [32%] women and n=13 [68%] men; median age 71, range 36-84 years) were recruited from a private hospital and cardiac physiotherapy clinic in Dublin, Ireland, to take part in individual semistructured interviews. To be included in this study, participants had to have a confirmed diagnosis of HF, be aged >18 years, be willing to participate, and have the capacity to provide informed consent. Exclusion criteria included not having a confirmed diagnosis of HF, being a child or adolescent, lack of English understanding, a cognitive impairment that would preclude the capacity to provide informed consent, and having rheumatic heart disease or severe aortic or mitral valvular heart disease. This was to ensure that within the criteria, a representative sample would be included so that the impact of HF could be explored.

##### Study Methods

Patient data were collected during single face-to-face interviews (17/19, 89%) and over the phone (2/19, 11%) when patients could not attend the interview in person. Demographic data such as age, sex, occupational status, marital status, class of HF, and presence of other medical conditions were collected. Open-ended questions were used to explore their perceptions on the use of DHT for the management of their condition and understand the factors to be considered in the design and implementation of DHT. The interviews were audio recorded, and a topic guide was developed based on the aims of the study to ensure consistency across the interviews (Multimedia Appendix 1).

##### Data Analysis

The interviews were transcribed verbatim and anonymized. An inductive, thematic analysis of the transcribed data was undertaken using a realist approach, whereby the researcher assumes that the opinions of the patients reflect their real perceptions and can be considered real [5]. A total of 2 researchers (RC and AK) analyzed the transcribed data following the protocol of Braun and Clarke [5]. This protocol comprises the researchers familiarizing themselves with the data, generating a list of initial codes relevant to the aims of the study, and refining these codes by grouping them into potential themes. The list of codes and themes was iteratively revised until an agreement was reached using NVivo (version 12; QSR International) and Microsoft Excel.

#### Literature Review

Clark et al [6] conducted a systematic review of qualitative studies that investigated the determinants of effective self-care in patients with HF. This review analyzed data from 49 qualitative studies that examined patients with HF and caregivers’ views and needs about the nature and determinants of effective self-care. This comprehensive work formulated a series of key skills that are crucial to the development of effective self-care in this population. The key skills highlighted by Clark et al were then combined with the findings of the semistructured interviews and the best practice statements on HF self-care promotion by the American Heart Association [4] and the European Society of Cardiology [3] and were carried forward into the ideation process.

### Step 2: Ideation

The aim of step 2 was to aggregate the findings from step 1 to formulate ideas and design concepts for a prototype solution to promote self-care behavior change. The barriers to and facilitators of the use of DHT and key design considerations identified in the qualitative interviews were first mapped to the determinants of effective self-care highlighted by Clark et al [6] and then subsequently to the relevant domain and intervention functions as listed in the Theoretical Domains Framework (TDF) [18]. The TDF is a theory-based framework that helps researchers understand their target behavior; identify its determinants; select intervention functions that best influence these determinants; and finally, select relevant behavior change techniques (BCTs) that are most effective toward these functions and determinants [18]. BCTs are the smallest, reproducible component of an intervention designed to change behavior either alone or in combination with other BCTs. Thus, once the desired intervention outcomes and intervention functions were mapped from the TDF, potential BCTs, as listed within the BCT Taxonomy version 1 [19], were identified using the web-based Theory and Techniques tool [20]. BCTs to be included in the DHT were selected based on their evidence or hypothesized links to TDF domains and intervention functions, along with their potential to be operationalized in a DHT, to ensure that the determinants of behavior were targeted within the contents of the system.

### Step 3: Design

The identified BCTs from step 2 were taken by the study team (a consultant cardiologist, a mobile developer, and 2 digital health clinical research physiotherapists), and a series of high-level requirements for the system were iteratively developed, taking into account the patient requirements identified in step 1, as well as clinical and technical feasibility. These requirements were then used to develop wireframe prototypes and technical requirements to guide mobile development.

### Step 4: Develop

The finalized wireframe design and requirements were developed into a version 1 prototype solution, which was then evaluated with a group of patients with HF in step 5. As detailed further in the Results section, the developed system comprised a mobile phone app, a Fitbit Charge 4 activity tracker (Fitbit Inc), and Aria Air smart scales (Google).

### Step 5: Test (Study 2)

#### Overview

The developed prototype solution was examined in a 2-week pilot evaluation phase. A mixed methods approach was used to evaluate the feasibility of the system, focusing on the acceptability, usability, demand, and practicality of the DHT system according to the participants [21]. In addition, this phase was used to identify any technical issues that needed to be addressed in a version 2 system and highlight any additional feature requirements identified by the patients that would be required in any further evaluation of the system. A 2-week period was deemed acceptable for this initial feasibility phase as it allowed sufficient time for users to familiarize themselves with the system, identify usability issues, and explore the practicality of using it within their daily life. This initial, short follow-up is commonly used in HCD studies [22,23].

#### Participants

A convenience sample of 9 participants (n=4 [44%] women and n=5 [56%] men; mean age 74, range 54-91 years) volunteered to participate in the study. Participants were recruited from a private hospital in Dublin, Ireland, and had previously been diagnosed with HF. This purposive sample allowed for the aggregation of usability and acceptability data pertaining to the initial system, facilitating the iterative development of a system for deployment in the full feasibility study. Previous research has shown that a sample size of approximately 9 participants is sufficient to reach data saturation in similar contexts [24]. Participants were deemed eligible if they could provide written informed consent; were previously diagnosed with HF; were under the care of Beacon Hospital Cardiology (aged ≥18 years); were under New York Heart Association classification 1 to 3; were open to the use of technology in the promotion of HF self-care; had access to an internet connection or mobile data; and were intellectually, visually, and auditorily capable of communicating with the investigator and understanding and complying with the requirements of the study. Participants were deemed ineligible if they were medically unstable or undergoing medical treatment judged not to be medically compatible by the investigator or if they had any skin condition that may affect the integrity of their skin when wearing the activity tracker.

#### Study Methods

The recruited participants were invited to an initial setup session at the hospital. Demographic data such as age, sex, and the highest level of education were collected at the beginning of the session. The participants then completed the European HF Self-care Behavior Scale (EHFScBS) [25,26] and the Minnesota Living with HF Questionnaire (MLwHFQ) [27,28]. These questionnaires were designed to evaluate self-care behaviors in patients with HF and the effect of HF treatments on the quality of life.

Following a setup and familiarization session with the first author (approximately 40 minutes), participants were asked to use the system as part of their usual daily routine for the following 2 weeks. During this period, the patients were asked to wear the Fitbit Charge 4 activity tracker on their wrist, take their weight every morning using the Fitbit Aria Air scales, and interact with the developed mobile app. A *check-in* symptom questionnaire was also completed 7 days into the trial period [29].

At the end of the 2-week period, individual semistructured interviews were completed over the phone and recorded with each participant. Open-ended questions were used to explore their perceptions of the acceptability, usability, and practicality of the system; understand their experiences pertaining to the impact of the system on their self-care behaviors; identify usability and user experience issues; and identify aspects that could improve the system (Multimedia Appendix 1). Before completing the interviews, participants also completed 3 questionnaires: System Usability Scale (SUS), a questionnaire designed to measure system usability [7]; Wearable Technology Motivation Scale (WTMS), a questionnaire based on the intrinsic needs listed within self-determination theory [30]; autonomy, competence, and relatedness and the Comfort Rating Scale (CRS), a questionnaire designed to assess the comfort of wearable devices across the dimensions of emotion, attachment, harm, perceived change, movement, and anxiety [31].

#### Data Analysis

The recorded interviews were transcribed verbatim and anonymized. The same thematic analysis approach was used as detailed in the *Step 1: Empathize* section.

The questionnaire data were scored using the appropriate standardized procedure for each questionnaire, and the scores were presented as medians and ranges. The MLwHFQ is broken down into 2 components, the physical and emotional dimensions, which are combined to form the total score. It is scored by summing each of the components, resulting in a score ranging from 0 to 105 (high impairment) [27]. The EHFScBS is scored by summing the components of the questionnaire, *z* score normalizing, and calculating the percentiles. This results in a score ranging from 0 to 100 (good self-care), with <30 deemed as inadequate [25]. The SUS is scored out of 40 but converted to a 0 to 100 scale as per the standard procedure, with >68 deemed acceptable and >80 considered excellent [32]. The CRS is scored by summing the components, resulting in a score from 0 to 120 (poor comfort) [31]. Finally, the WTMS is scored by calculating the average score across the different components for each participant, resulting in a score ranging from 0 to 7 (extremely motivated) [30]. In addition, adherence was determined by identifying the number of days a user wore the Fitbit device throughout the day and recording their weight.

### Ethics Approval

The study received ethical approval from the Beacon Hospital Research Ethics Committee (BEA0114 and BEA0151), and written informed consent was obtained from all participants before commencing the study.

## Results

### Step 1: Empathize

#### Study 1: Semistructured Interviews

The full and detailed results of these interviews are listed elsewhere [17]. In summary, the results highlight that although patients are generally interested in engaging with technology, aspects such as technology literacy, previous exposure to technology, age, and lack of perceived usefulness are all important factors in driving whether it is adopted or not. Furthermore, several key factors to be considered in the design of a DHT were identified (Textbox 1).

**Key factors identified by patients with heart failure.**

**Key factors and details (required elements of the factor)**

**Ease of use**

- Easy to use, with appropriately sized font and easily understandable language and health data

**Vital sign monitoring**

- Support vital sign (eg, resting heart rate, blood pressure, and oxygen saturation) monitoring and actuation

**Physical activity promotion**

- Support physical activity monitoring and guide patients in pacing strategies and exercise targets

**Weight and fluid control**

- Facilitate weight and fluid management through daily weight tracking

**Feedback loops**

- Feedback from the vitals and other health data should be patient specific and not generic comparisons with healthy nonclinical populations

**Health data analytics**

- Background analysis of the collected health data to help proactively inform about potential deterioration and facilitate help seeking

**Reminders**

- Include a reminder feature for medications and appointments

**Medical information**

- Educational information should be included from a reliable source and should not be intimidating for the user
- Provide patients with the requisites to participate in their own condition management

**Included devices**

- Any devices associated with the digital health technology should not be medically oriented to reduce negative associations of having a chronic illness

**Diet tracking**

- Ability to record calorie intake, similar to solutions such as MyFitnessPal

**Emergency information**

- Medical information about their condition and medications for emergency situations

**Social support**

- A social networking aspect to facilitate social support

#### Literature Review

In the Clark et al [6] systematic review investigating the determinants of effective self-care, the authors highlighted that patients demonstrate or report a low knowledge of HF or lack of understanding of self-care behaviors through the following:

- Lack of recall about the basic elements of the nature of HF
- Apparent misattribution of HF symptoms to other conditions, age, or medication
- Low understanding of the links between signs or symptoms of HF
- Absence of references to the importance of weight management or monitoring
- Avoidance or low awareness of the severity of HF

In addition, they identified that HF self-care was shared between the patients and informal caregivers.

The skills for effective self-care, as identified in this review, are presented in Textbox 2 [6]. In addition to these, the international best practice statements on HF self-care by the American Heart Association [4] and the European Society of Cardiology [3] highlight that the desired goal of supporting self-care in HF is to improve quality of life, reduce the need for unnecessary hospitalizations, and reduce the risk of early mortality.

**Key skills for effective self-care.**

**Skill and description**

**Integrating self-care within normal life patterns**

- It is helpful to incorporate self-care into daily life as it facilitates adherence.

**Early detection of signs and symptoms**

- Patients have an overreliance on subjective symptoms.
- Patients are often only able to identify relevant changes associated with worsening conditions formatively with experience.

**Caregivers, their knowledge, and the range of heart failure**

- Caregivers typically provide substantial support with medication and diet adherence.
- Caregivers rarely help with daily weighing, fluid restriction, physical activity, and timely help seeking.

**Caregivers foster patient independence**

- Poor caregiver support can lead to difficulties with managing self-care, although a lack of support is not limited to those who live alone.

### Step 2: Ideate

On the basis of the key determinants of effective self-care, combined with the best practice recommendations and patient interviews, a number of behavioral outcomes were identified as a focus for the development of DHT: (1) engagement in regular physical activity; (2) management of fluid status daily through weight monitoring; (3) adherence to medication; (4) understanding the signs and symptoms of HF, including the consequences of an exacerbation; and (5) seeking medical help appropriately when required.

The barriers to and facilitators of self-care behavior were mapped to the determinants of behavior and appropriate TDF domains (Multimedia Appendix 2). A pragmatic decision was taken to focus initially on the key needs and requirements of version 1 of the system. If this simple version is successful in supporting self-care in HF, features designed to address additional behaviors can be iteratively added to the solution. This incremental approach is in line with recommendations for the development of complex interventions [33,34] and has previously been applied in the development of DHT for chronic disease management [15]. Subsequently, the determinants of behavior were mapped to the TDF domains, appropriate intervention functions, and evidence-based BCTs as listed within BCT Taxonomy version 1 to ensure that the determinants of behavior were targeted throughout the contents of the system (Multimedia Appendix 3).

### Step 3: Design

#### Overview

The system was required to comprise a cross-platform (iOS or Android) mobile app capable of linking to a consumer activity tracker and smart scales. Owing to the common use of Fitbit activity trackers and smartwatches, the Fitbit Charge 4 and Aria Air Smart scales were chosen for use in this system. Fitbit activity trackers have previously been shown to demonstrate sufficient consistency in their measurement for relative comparisons of within-subject use in community-dwelling older adults and patients with HF [35-37]. The designed prototype was broadly divided into five sections—(1) advice, (2) symptom reporting, (3) activity tracker and scale data (exercise, weight, heart rate, and sleep), and (4) medication reminders and other vital sign tracking—all targeted through the inclusion of specific BCTs (detailed in Multimedia Appendix 3). The wireframe design for the prototype system is presented in Multimedia Appendix 4.

#### Advice

This section was designed to provide targeted educational content through a series of animated explainer videos and to-camera information videos by a consultant cardiologist. In addition, *how to* instructional videos were included throughout the system to guide patients in the use of the DHT. Additional information on educational content and BCT mapping is provided in Multimedia Appendix 5.

#### Symptom Report

This section allowed patients to complete a short symptom check-in questionnaire if they felt that their symptoms had changed in the preceding days. Once completed, the questionnaire was sent to the Beacon Hospital Cardiology Team, which would follow up within 24 to 48 hours to facilitate timely help seeking.

#### Fitbit/Scales Data

To facilitate the promotion of paced exercise, sleep hygiene, heart rate monitoring, and weight and fluid retention tracking, the mobile app was integrated with the Fitbit activity tracker and smart weighing scales to help the integration of self-care into daily life and detection of early signs and symptoms and prompt early help-seeking. The system was designed to automatically track patients’ exercise (step count), sleep time, resting heart rate, and weight. In addition, to support patients in understanding how these measurements fluctuate over time and how a change from their *normal* is presented, these end points were visually displayed within the mobile app. The individual’s baseline for any of the 4 end points was displayed as a green band, termed their *personal baseline*. This was also designed to automatically monitor for alterations in these end points from the individuals’ *personal baseline*. When a patient’s weight, resting heart rate, sleep time, or step count changed by >2 SDs of their *personal baseline* for 3 consecutive days within the past 5 days, participants could be prompted with a symptom questionnaire, which would be shared with the cardiology care team for evaluation [38]. However, because of the short 2-week trial duration, only the visual feedback of the *personal baseline* was used. Future evaluations of the long-term use of this system will incorporate fully automated monitoring and symptom questionnaire triggering. Additional information on the computation of the *personal baseline* and trigger logic can be found in Multimedia Appendix 6 [38].

#### Medication Reminders

As patients with HF are typically required to take a large number of medications, the ability to add medications and set time-based reminders was incorporated into the mobile app.

#### Other Vital Sign Tracking and Reminders

The capacity to add additional vital sign end points (eg, blood glucose, blood pressure, and spirometry) was incorporated into the design to facilitate patients with HF with relevant comorbidities in an attempt to centralize most of their monitoring requirements. Users could also set time-based reminders to facilitate adherence to the monitoring.

### Step 4: Develop

**Figure 2:**
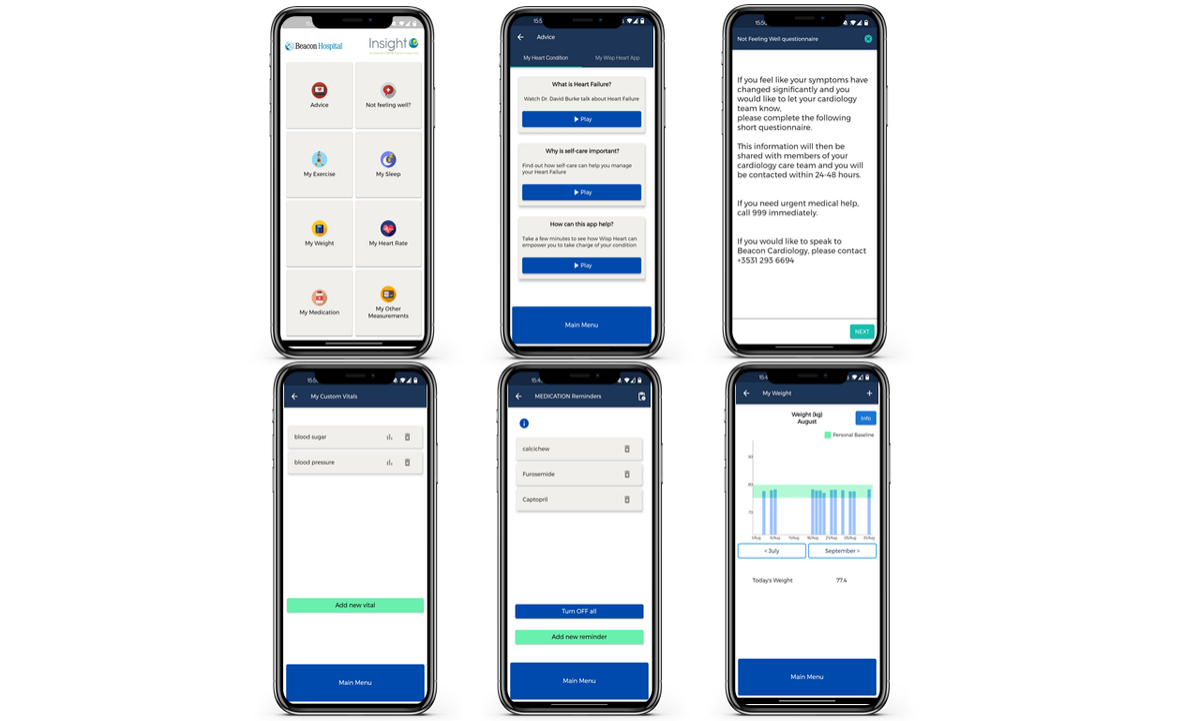
Screenshots from the mobile app detailing the main menu, advice section, symptom report, other vital sign tracker, medication tracker, and weight tracker screens.

The finalized wireframe design and requirements were then developed into a version 1 prototype mobile app (Figure 2). A video demonstrating the mobile app can be viewed in the Johnston study [39].

### Step 5: Test

#### Overview

All 9 participants completed the 2-week trial period, semistructured interviews, and evaluation questionnaires. Table 1 presents the demographic characteristics of the participants recruited during this phase of the research.

During the 14-day trial, 78% (7/9) of participants wore the watch for the entire 14 days, whereas 22% (2/9) of participants wore it for 13 of the 14 days. Similarly, 56% (5/9) of participants recorded their weight on all 14 days, whereas the minimum number of days on which weight was recorded was 10. The median scores for the MLwHFQ and EHFScBS were 24 (0-78) and 32 (10-93), respectively. For the EHFScBS, 44% (4/9) of participants were deemed to have inadequate self-care. Table 2 details the responses for the SUS, CRS, and WTMS, which were collated at the end of the 2-week trial period, all of which demonstrate the acceptability of the system to participants and the impact on motivation to exercise.

**Figure 3:**
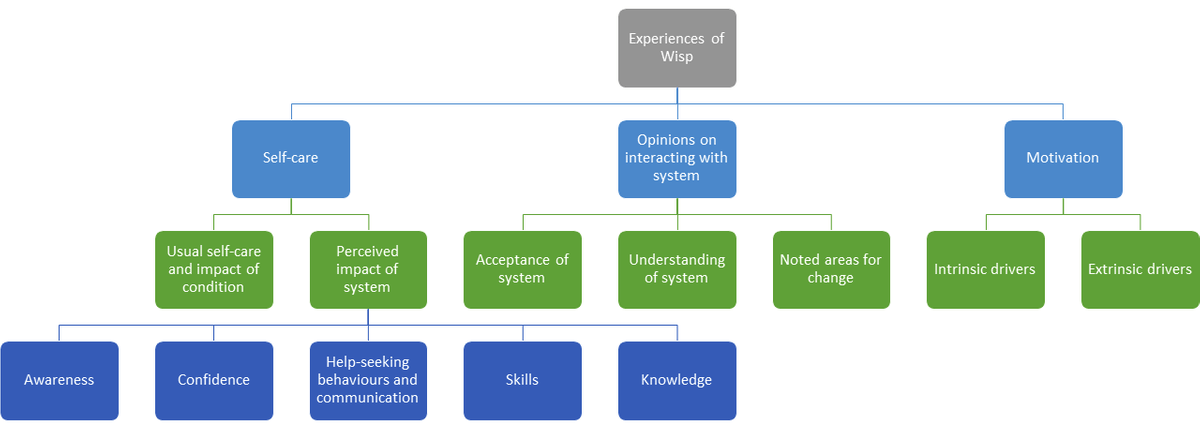
Themes and subthemes from the semistructured interview analysis.

The analysis of the semistructured interview data identified 3 key themes and their associated subthemes (Figure 3).

**Table 1:** Demographic data for the recruited patients with heart failure (N=9).

Demographic details			Participants, n (%)
**Lives with**			
	Spouse	6 (67)	
	Family	1 (11)	
	Alone	2 (22)	
**Marital status**			
	Married	6 (67)	
	Single	2 (22)	
	Widowed	1 (11)	
**Phone operating system type**			
	iOS	2 (22)	
	Android	7 (78)	
**Contributed own Fitbit**			
	Yes	1 (11)	
	No	8 (89)	
**New York Heart Association classification**			
	1	3 (33)	
	2	4 (44)	
	3	2 (22)	
	4	0 (0)	

**Table 2:** Questionnaire descriptive statistics.

Questionnaire	Values, median (range)
System Usability Scale (0-100)	92.5 (72.5-100)
Comfort Rating Scale (0-120)	6 (0-38)
Wearable Technology Motivation Scale (0-7)	5.9 (5.1-7)

#### Self-care

##### Overview

Within self-care, 2 key subthemes were evident: usual self-care and the impact of the condition and perceived impact of system/changes resulting from it. Within the *Perceived impact of the system*, 4 additional subthemes (awareness, confidence, help-seeking behaviors and communication, and skills and knowledge) were identified. These themes are closely related to the focus of self-care—autonomy, independence, and a person’s responsibility for healthy behaviors—and the development of activities required to manage and monitor health conditions [3].

##### Usual Self-care and Impact of Condition

Patients highlighted that HF significantly and negatively affected their confidence and perceived ability to engage in self-care promoting activities such as physical activity, thus affecting their quality of life:

before I got the blockage cleared in my heart, my breathing got to the point that I felt that every step I took I was pushing a boulder in front of me, or dragging one behind me, one or the other, I just didn’t have the energy to do anything and it knocked me [sic] confidence an awful lot because I’ve never had to build myself back up from doing two, three years of doing nothing, whereas before you could get a period with six months where you’re doing nothing and you have to go back and start being physical again,... it’s not that I’m afraid to get out and do anything it’s just that I felt that I’ve been so long doing nothing I didn’t have it in me, and my breathing’s still not 100%, so it sort of scared me

Male, 54 years, inadequate self-care

Regarding usual self-care behaviors, participants highlighted that they were aware of helpful self-care behaviors; however, they found it difficult to build and continue a structured routine. For example, when discussing setting a sleep routine, one of the participants detailed how she struggled to build good sleep hygiene practices despite knowing what was required:

You know, the first night I did it, it worked great. But then the next night I wanted to watch a programme that was on a bit late, a documentary that was on a bit late, and I thought I’m not going to get to bed till after midnight. And I didn’t. And I haven’t done it since, properly. But I am going to try and do it now again. I’m going to try and start doing it.

Female, 81 years, inadequate self-care

Similarly, the difficulty in maintaining consistency in routines was highlighted when discussing physical activity:

Well I would have been very inconsistent. I would spend a week doing great, I’d go out walking every day and then the following week I wouldn’t go out at all, maybe only one or two days or even less. So, it [FitBit] made a huge difference to the consistency side of things.

Male, 64 years, inadequate self-care

##### Perceived Impact of System/Changes Resulting From it

Overall, all patients reported a positive experience using the version 1 prototype system and expressed an interest in its continued use beyond the evaluation. When focusing on the perceived impact of the system and the changes resulting from its use, the topics of awareness, confidence, help-seeking behaviors and communication, and skills and knowledge emerged.

- Awareness: The participants stated that the use of the system had a positive impact on their awareness of their condition, self-care behaviors, and lifestyle. In particular, participants noted that while using the system, they developed an increased awareness and consciousness of their physical activity levels and weight:

Apart from, now, keeping a closer eye I suppose on the weight. And now I’ll be keeping a closer eye on my exercise as well, with it. So over all, it has, made me...I wouldn’t say made me, but convinced me to focus a little bit more on what your doing, rather than just doing it. Focusing a little bit more on trying to get a little bit more information about yourself as you are doing stuff.

Male, 68 years, adequate self-care

Although the participants noted that they could see value and comfort in sleep and resting heart rate tracking, their understanding of the intended specific purpose of these measures was less clear:

my pattern of sleep or going to bed or anything like that never changed, but it just gives me the picture that I am sleeping for eight-and-a-half-hours every night most nights and you know it’s a great comfort to know that you’re doing that.

Female, 91 years, inadequate self-care

That is very comforting to know what your resting heartrate is and mine has been literally the same the whole way through the test, I think it’s gone from about 64 and the highest it’s gone resting is about 68, and it has stayed that way every single day, for the whole of the test. Which, it is comforting to know.

Female, 81 years, inadequate self-care

Importantly, several participants noted that over the course of the trial, they developed a greater awareness of the impact that positive self-care behaviors, such as increasing physical activity and proactively monitoring weight and fluid consumption, had on their symptoms and quality of life:

No, I think I get pains in my joints from the fluid, in my knees mostly and in my hips, but the exercise helps with that. Whereas before I was doing nothing and when I was sitting down I’d get pains in my hip joints and when I go to move then my knees felt like they were dislocating, but I, it doesn’t, the exercise, a walk helps with that.

Male, 54 years, inadequate self-care

One day it frightened me, because I went up to, eh, I went up to 0.9...[pause]...Up to 67.9 or something. And I got an awful fright because I’m going upwards or downwards. Or not static. But I realised I hadn’t been to the bathroom, I hadn’t been to the toilet sort of, for two days. Now I wasn’t constipated, but I just hadn’t been. There was a big difference the next day, from that reading to the next day. There was nearly a kilo. I wasn’t eating anything different as such, you know I still have my three meals a day. I’m trying to stick to my three meals a day. Now I do graze at night time. You know what I’m talking about?

Female, 81 years, inadequate self-care

- Confidence: Participants also stated that using the system provided them with increased confidence in their condition, providing them with an increased sense of control over themselves and their conditions. Furthermore, using the system made them aware of how capable they were of engaging in self-care behaviors such as increasing their physical activity, thus further increasing their confidence:

It just made me more secure, if you like in a sense, that I knew nothing was happening, that I was still the same, that I wasn’t putting on weight, or losing too much weight, excuse me, but other than that, no, just nice to look at it.

Female, 91 years, adequate self-care

It [the system] has, it’s given me a lot more confidence around it [physical activity], where I was a bit, not that I was scared or anything but I was apprehensive about doing anything anymore, and the kids were sort of treating me like I was made out of glass, you know that sort of way, so, and I know my heart failure isn’t as serious as it could be, but it was just that I got a blockage in my foot and I got a blockage in my heart and just everything seemed to collapse in on me at the same time and it sort of knocked my confidence about everything really, so yeah I, listen I don’t feel that way now, I know I’m not in any real danger where I wasn’t really in any real danger once I’d been sorted out anyway but I just felt a lot, I just felt I wasn’t doing well, you know that sort of way, I was afraid to do stuff, my wife was afraid to do anything with me, if I came home tired from work she would say go to bed, which is totally the wrong thing to do, but yeah it has certainly helped me in that respect, it has taught me an awful lot about what I can and can’t do, what I should and shouldn’t do, you know? So yeah listen I’m a huge advocate for it, I think it’s great, I really think it’s great and so does my wife.

Male, 54 years, inadequate self-care

- Help-seeking behaviors and communication: The participants were universally positive about the prospect of the system monitoring various end points. They discussed that being monitored would provide them with increased comfort and that it would create an additional safety net to facilitate help seeking in the event that their condition changed. Interestingly, participants did not appear to mind whether their data was being monitored by a human or a computer-based system, although it is unclear how much they understood the difference between the two. This was particularly clear when the topic of data security arose. No participants had any privacy concerns and trusted that as the system was managed by health care and research institutions, there were sufficient data protection procedures in place:

I think [the monitoring is] absolutely brilliant. I think it is a super idea that that sort of apparatus can be used for people to see what is happening to you. Whereas I might not pick up on something that it would be going else where and experts would be able to look at it and say something is happening here and we can see what the situation is. I think it is a super idea.

Male, 74 years, inadequate self-care

It wouldn’t make any difference once it had monitored if I had something wrong with me. I think it’s great to have it monitored on all the time, and the fact, the way I look at it is, if it’s being monitored then a human is looking then at the monitoring and then sees the way things are going, it’s a two-part thing, really.

Female, 81 years, inadequate self-care

Participants appeared to feel that simply having access to the data collected by the system would facilitate communication about their condition with health care professionals. Importantly, one of the participants (male, 84 years, adequate self-care) used the system to share an acute change in symptoms with the hospital’s cardiology care team and reported a recent increase in dizziness. This process initiated consultation with the cardiology care team:

As I said, when I go back to talk to the consultant, I feel like I can have a better conversation with him about my condition and if I have any questions, I feel like I am in a better position to ask. And when he is speaking to me, I am able to give him better information than I was before. So other than that, I think it was great and well worth doing.

Male, 68 years, adequate self-care

- Knowledge: Linked closely to increased confidence and awareness, the participants also perceived that the data provided within the app increased their knowledge about themselves and their conditions and helped them make more informed self-care decisions. However, despite this, many of the participants also felt that the educational content throughout the app was relatively basic and did not provide them with much new information from what they would have obtained at diagnosis:

Well it did because it made me aware that I had a heart condition and it’s making me think more of my health, but I’ve always been reasonable about getting out and doing things, I don’t sit around and that sort of thing but the heart, it’s making me feel safer, like having that set there and knowing, it’s like as if I have help on my doorstep.

Female, 81 years, inadequate self-care

I was going out for one long walk, and I over did it one day when I was out for an hour and a half and I felt a bit...I didn’t feel great after it, so I decided I’d change. And I saw something in the advice about being active every hour or something like that. I don’t know how it said it. But this is very beneficial to my arthritis as well as I was having back trouble. I think it is improving, so touch wood, I think it is improving since I have gone onto this new regime and it was thanks to the app I wouldn’t have found anything.

Male, 64 years, inadequate self-care

I did, I watched the videos. They’re very simple and very quick so they’re easy to follow you know? Again a lot of the information I’d know anyway just from my time in it but yeah they’re helpful, they are definitely helpful.

Male, 54 years, inadequate self-care

Although participants generally reported a positive impact of the system on their knowledge of their condition, some participants demonstrated a perception that they lacked control over the system end points other than physical activity. This suggests that the potential impact of the other end points was not clear or that participants were not aware that no change should be seen as a good thing in some cases:

I said it made me probably do a bit more exercise sort of thing. Other than that, at the exercise side of it, I probably wouldn’t have seen anything other than that as being beneficial you know. Not because...That’s probably the only thing on it that you can judge yourself into what you’re doing physically and the exercise end of it tells you weather you’re doing it or not doing it. It gives you the incentive to do a little bit more. Other than that, the other things I don’t think I can do an awful lot about the sleep. At the moment I cant do an awful lot about the sleep...As hard as I’m trying. And eh, the heart rate is pretty good and explains itself.

Male, 74 years, inadequate self-care

- Skills: Participants perceived that using the system over the 2-week period helped them develop self-care skills that they felt they lacked before the trial. Specifically, they highlighted that being able to track physical activity allowed them to set measurable goals for activity and promoted structured weight and fluid monitoring routines. Furthermore, many noted that they used the heart rate function on the Fitbit watch to help guide pacing and recovery during bouts of physical activity:

oh big time, everything is based on it now at the moment. First thing in the morning I do the weight and I’m very aware that every hour or so that I get up and get off sitting on my arse looking at the television and I do a thousand steps or so and I accumulate over 10,000 before the day is over, and also the climbing of the stairs is good and all that. Oh yes it has impacted big time on my life.

Male, 64 years, inadequate self-care

It changed, where before, I was sort of, where I said if I was cutting the grass, doing anything physical like that I would sort of feel, I’m getting a little warm here, are you doing a bit too much or that carry on. Now over the last fortnight, what I was doing is if I felt like that I would just look at the heart rate on the fitbit and see it had gone up, and if that’s a bit high just take a break for a few minutes, and that type of thing. And that has worked very well for me, because I felt by doing that, I am able to do a lot more during the day.

Male, 68 years, adequate self-care

When asked about medication and other vital sign tracking, participants’ responses toward these functions were mixed. Most participants did not use these functions as they reported their own strategies for medication adherence. However, 22% (2/9) of participants reported that they used it to keep on top of medications that followed a different dose timing requirement for most of their medications:

no I didn’t use either of those. The reason for that is I wanted to sort of put myself in a position where I was looking at stuff myself. Now if I were to use this thing long term, I probably would.

Male, 68 years, adequate self-care

#### Opinions on Interacting With System

When considering participants’ interactions with the system, 3 key subthemes emerged: acceptance of the system, understanding of the system, and areas for change.

##### Acceptance of the System

When talking about their experience over the 2-week trial, most participants communicated that they were initially intimidated by the thought of learning to use new technology and were scared of using the system incorrectly. However, they universally expressed that they quickly became comfortable with the system and found it easy to use:

Yeah, well starting off I was nervous, and the reason I was nervous is I wanted to get it right, but when I got it set up and going, I felt pretty confident, but it took a couple of days for me to be confident.

Female, 81 years, adequate self-care

very easy to use...very easy to use. I was a bit apprehensive at the start. Because as I said I am not a techie person, I’m not into gadgets or anything like that. A bit apprehensive, but it only took me a day or two to fall into line with it and find then my way around with it. I found it very useful. One thing led onto another and I find it very useful now.

Male, 68 years, inadequate self-care

##### Understanding of the System

Participants generally had a good understanding of the system and found it easy to use and beneficial to their self-care. However, despite feeling that the system was generally easy to use, some participants discussed particular incidents where they were not completely sure of the functioning of the system. Furthermore, although there was no universal struggle with regard to broadly understanding how the system worked, individuals reported difficulties with certain aspects of its functionality. For instance, participants were often unable to clearly distinguish between the proprietary Fitbit app and the version 1 DHT system. Others struggled with reading the digital weighing scales. In addition, one of the participants expressed a lack of understanding of the purpose and functionality of the heart rate monitoring aspect. Despite the Fitbit activity tracker simply measuring the heart rate, they discussed how they were concerned about *an irregular heart*, suggesting that they were under the impression that the device was similar to an electrocardiogram:

My experience with it has been very positive. It’s very like taking, or somebody injecting you with something without you knowing about it, because it just sort of, I don’t know, I’ve wore FitBits before I think, but they didn’t do this to me sort of, you know what I mean? It didn’t get me thinking about it.

Male, 57 years, inadequate self-care

I started using the other app a bit, I got into using the other app and adding in the different things. Now for example, for the resting heart rate it just gives the resting heart rate for the day. The wisp [Version 1 DHT] app doesn’t give you, as far as I know, doesn’t give you a graph showing the highs and the lows.

Male, 74 years, inadequate self-care

Well it would because when things start to happen to you you’re worried about an irregular heart or something, that’s what I suffered from originally, and there’s none of that and I don’t pick anything up and it’s kind of a safeguard.

Male, 84 years, adequate self-care

##### Areas for Change

Despite their generally positive experience using the system, participants noted some technological glitches that occurred during the trial and aspects that they would like to see improved (Table 3).

**Table 3:** Identified areas for refinement of the digital health technology.

Component	Details	Solution
Font size changes	Although adaptive scaling was implemented within the version 1 system, it was not optimized for the largest font sizes on small screens.	Updated when identified within the trial
Smart scales not compatible with implanted medical devices	Participants who had a fitted medical device requested that an alternative smart scale could be used to reduce the need for manual input.	An alternative scale has been identified for version 2
Difficulty in setting up smart scales	Of 9 participants, 8 (89%) required technical support when setting up the Fitbit Smart scales because of the need for a Wi-Fi connection. Therefore, an alternative, easier to set up scale was requested.	An alternative scale has been identified for version 2
Decimal point issue on iOS	iOS users initially identified the inability to input a decimal point when manually recording weight and other vital signs.	Updated when identified within the trial
Screen time-out in videos	One of the participants noted that their screen would time out during educational videos.	Updated when identified within-trial
Information button not obvious	Participants were not initially aware of the location of the information button throughout the version 1 system.	Will be incorporated into version 2
Intermittent issue with displaying activity and sleep data	Approximately 33% (3/9) of participants experienced a technical glitch lasting 3 days whereby “Todays Sleep” and “Todays Weight” did not display.	Updated when identified within-trial
Within-day heart rate data alongside resting heart rate	Approximately 33% (3/9) of participants expressed an interest in being able to visualize within-day heart rate data within the version 1 system to help inform pacing.	Will be incorporated into version 2

#### Motivation

Across the board, participants expressed that using the DHT system over the 2-week trial motivated them to engage in effective self-care behaviors, with both intrinsic and extrinsic motivators at play.

##### Intrinsic Drivers

Participants commonly identified that they were keen to be involved with research activities that may help them or others with HF and were motivated by the nature of being on a *program*. However, it would appear that the key motivational driver for them interacting with the system was increased perception of both autonomy and competence. Specifically, seeing changes in their data based on their own behaviors was an important element of this:

well I honestly feel that if you go and support other people, no matter what it is. Whether it is this or something else you’re doing, you get something back. No matter what you do you get something back. You go and you volunteer to do something, it doesn’t matter what it is. You do something in the community and you always get paid back. You always get something back from it. So, you don’t do it for getting something back, but it always seems to come your way.

Male, 68 years, inadequate self-care

I feel more confident in what I can do. And that...it feels like I am in control of what I can do. And watch what I can do and know how far I can push myself and that sort of thing. I found it very good from that point of view.

Male, 68 years, adequate self-care

but as I say when I look at the scales, I know, like the 800 grams I put on yesterday which is nearly 2 pounds, you don’t put that on overnight when you haven’t eaten anything extra, and you haven’t drank anything extra, so I knew that yesterday I had to stop drinking water and have less tea and all of that stuff to try and, and I’m raging a bit at myself because it would have told me whether I was successful or not today but it is handy knowing day to day what you weigh.

Male, 54 years, inadequate self-care

##### Extrinsic Drivers

Participants also discussed how many of the motivating factors for engaging in self-care activities were driven by extrinsic factors. Specifically, participants reported feeling safe as they perceived they were being *watched* by someone. Their social support structures encouraged the use of the system, and it also acted as a mechanism to encourage conversation with friends and family about health-promoting activities:

But I certainly will talk, like I do discuss it with her, I discuss it with her quite regularly, is this normal and is that normal, you know, and just her, like she set the target for me at 8000, and that’s going back quite some time ago.

Female, 71 years, adequate self-care

I found I was doing it and the lads were doing it. I said I look, I have this fitbit—“is that expensive that.” They start doing it as well. That type of thing. So I just pace off and the lads would pace off as well. That sort of thing was good. It was sort of like, four people playing together and rather than one person pacing off, everyone was. It was sort of a fun thing to do, I’ll put it that way. everyone was sort of happy to do it and we all fell in line with one another.

Male, 68 years, adequate self-care

## Discussion

### Principal Findings

This study provides a detailed description of the design, development, and initial evaluation of an evidence-based, human-centered DHT designed to promote effective self-care in patients with HF. The mixed methods evaluation demonstrated that it was generally easy to use, positively affected their motivation to engage in key self-care behaviors, provided them with skills and perceived knowledge that made them more aware of the importance of self-care behaviors, positively influenced their confidence, and facilitated help seeking. This process also identified aspects of the system that required further attention before progressing to the next stage of development and evaluation.

Although this research is not the first to use technology in the quest to promote self-care in HF, the effectiveness of previous solutions has been relatively inconsistent. A systematic review by Cajita et al [40] investigating mobile health–based HF interventions demonstrated that the impact of the developed systems on end points such as all-cause mortality, cardiovascular mortality, HF-related hospitalizations, length of stay, New York Heart Association functional class, left ventricular ejection fraction, quality of life, and self-care were inconsistent at best. One of the key reasons put forward for the lack of success of these solutions is their failure to thoroughly integrate users’ perspectives at the point of design. This results in the development of solutions that are not user-centric and will likely not drive continued engagement, failing to affect behavior in the long term [12-14]. To address this limitation, our research followed a best practice HCD approach, as outlined in the International Organization for Standardization 9241-210:2019 regulations, to ensure the development of a system that places the user at the center, maximizing the potential positive impact on self-care behaviors and quality of life [16]. This process involved initial patient interviews and HF literature consultations, followed by behavior change mapping to develop evidence-informed design concepts. These were then used to guide the development of the technical requirements and initial wireframe prototyping. A version 1 system was then developed and subsequently evaluated using a mixed methods approach with a cohort of patients with HF. This theory-driven approach ensured that the evidence backed the development of a system that was not only usable but was best placed to drive self-care behavior change. This approach of combining HCD with behavior change theory has led to the successful development of other DHTs, such as that developed by Korpershoek et al [15] for chronic obstructive pulmonary disease (COPD). Korpershoek et al [15] used an HCD process to design and develop a system to enhance self-management in patients with COPD. Following an approach similar to that followed in our research, they demonstrated that the developed DHT met the needs and preferences of patients with COPD and therefore had a high potential to be effective in reducing exacerbation impact. Although within a different chronic disease context, the overarching principles remain the same, demonstrating the value of engaging in an HCD approach. One of the key aspects of the developed system is the use of a consumer-wearable activity tracker. This approach was leveraged because of the outcome of the initial qualitative interviews conducted in study 1, where patients highlighted the need for devices that did not draw attention to their condition. Although this is one of the first studies to outline the comprehensive development of DHT-leveraging consumer technology for this population, similar approaches have been used within the context of cardiac rehabilitation. The iCardia system was developed using user-centered design approaches and was designed to support remote monitoring and health coaching in cardiac rehabilitation. Interestingly, the iCardia system predominantly centered on remote monitoring by specialists, whereas the DHT described in this paper was designed to empower patients to take control of their condition and engage in effective self-care behaviors. This is an important distinction, as it is acknowledged that engaging in effective HF self-care and disease management is central to reducing the burden on patients and the health system [3-5].

The use of a science-driven approach to design within this work has led to the development of a solution that may be more likely to be used by patients with HF and successfully promote self-care behaviors, positively influencing the impact of their condition on their quality of life. However, further research is required to investigate this in full. High engagement with the technology was highlighted by excellent compliance with wearing the activity tracker and completing daily weighing during the 14-day trial. Furthermore, although the 2-week trial did not lend itself to robustly evaluate the longitudinal impact on self-care behaviors, the results of the EHFScBS questionnaire completed at enrollment highlight the impact of the system on behaviors in the short term. For example, only 33% (3/9) of participants initially reported completing daily weighing at the commencement of the study. Despite this, during the 2-week trial, 56% (5/9) of participants weighed themselves every day, whereas the remaining 44% (4/9) of participants weighed themselves on days 13, 12, 11, and 10. Furthermore, the qualitative analysis highlighted that using the system resulted in increased awareness and consciousness of their weight, noting the impact of lifestyle factors on fluid retention and associated increases in weight. This indicates that the solution may address the key behavior outcome of facilitating the daily management of fluid status through weight monitoring (key target 2). Similarly, perceived increases in awareness and self-care skills were identified by patients in relation to physical activity behaviors (key target 1), with many participants identifying that the system increased their motivation to engage in exercise, aided goal setting/achievement, and helped them with pacing strategies. Importantly, many participants also identified a positive relationship between the perceived increase in physical activity and improvement in their symptoms. The impact on motivation to engage in physical activity was also highlighted by the high WTMS score (median 5.9, range 5.1-7). In addition, the effect on key aspects of self-care behavior is of note, as an inability to link relevant changes with an evolving condition has been identified as a key trait in patients with HF [6]. The short 2-week trial suggests a positive impact on self-care behaviors, along with excellent engagement with technology. However, it is possible that engagement may decrease over extended periods, reducing the positive impact on self-care behaviors [41]. As such, it is planned that future research will investigate the impact of this technology over a longer period.

The key targets of the solution were to support patients in understanding the signs and symptoms of HF and facilitate timely help-seeking behaviors. Although it was beyond the scope of this initial evaluation to thoroughly investigate this, participants reported an increase in their awareness of their condition and how self-care behaviors can positively affect their condition. Furthermore, one of the participants (male aged 84 years) used the *not feeling well* functionality to report a recent increase in dizziness to the cardiology team during the 2-week trial. This resulted in prompt consultation and review with the cardiology team to determine if the intervention was further required. In this instance, the participant showed the ability to link changes in symptoms with a potential alteration in their condition, with the system facilitating timely help seeking. The final key target of the solution was to promote medication adherence. Despite participants in the initial study requesting such a feature in a digital solution, only 22% (2/9) of participants used the medication reminders. The qualitative interviews highlighted that the main reason for this was that most participants reported having a relatively simple medication regime that required most medications to be taken in the morning or evening. Indeed, the participants reported having their own reminder systems or triggers in place. Why these appear to be more easily integrated into their routines than other self-care behaviors needs to be explored in greater depth. Furthermore, it must be considered that self-reported adherence to medication regimens may not reflect true adherence [42]. The 2 participants who used the medication reminder component did so for individual medications that did not follow their typical regimen. As such, although most participants did not engage with this feature during the 2-week trial, it may still be useful for the selection of patients who have more complex dosing regimens.

The solution developed in this process focused on key skills, as informed by the *empathize* stage. Although a broader intervention that endeavored to incorporate all aspects of HF self-care could have been attempted, it was decided that a pragmatic approach to the development of a simple targeted system would be most appropriate, focusing on key behaviors identified by Clark et al [6] and addressing the key needs highlighted during requirement gathering (study 1). It is envisaged that if the version 1 system is successful in driving targeted behavior change, features designed to address additional behaviors could be iteratively added to the solution. This stepwise approach aligns with the guidelines for the development of complex interventions [33,34] and has been applied in the development of chronic disease management DHTs [15].

A key limitation of the evaluation component of this study was that participants were required to be smartphone users and have access to the internet to ensure an adequate level of technology literacy. Although this results in a nonrepresentative sample of the HF population, the key aim of the study was to investigate the perceived impact of the system on self-care behaviors without the requirement to provide extensive training in smartphone use before being able to interact with the newly developed system. This factor reduces the generalizability of these findings to wider HF cohorts, and future HCD efforts should focus on identifying the additional features and/or training required to ensure suitability for a wider, more representative sample of patients with HF. It should also be considered that over time, it is likely that a high proportion of patients with HF will be technologically literate smartphone users.

Despite the promise of these findings, the design, development, and initial 2-week evaluation form the first key steps in the process; however, it is clear that further research is required before this system is incorporated into clinical practice. To do so, we are engaged in a 6-month observational trial to investigate the feasibility and utility of this system; establish whether engagement remains high; and observationally evaluate whether the solution affects end points such as quality of life, self-care behaviors, hospitalizations, and mortality. Furthermore, we seek to understand how best to incorporate the developed technology within a health care system to ensure that it fits into the daily practices of health care professionals. If this work is successful, a full-scale evaluation within a randomized control trial may be warranted to investigate whether the solution is efficacious when compared with standard care.

### Conclusions

This study describes in detail the HCD approach used in the development of a DHT to promote self-care in patients with HF. This science-informed methodology has resulted in the development of a system that patients indicate is easy to use, positively affected their confidence and motivation to engage in key self-care behaviors, provided them with skills and knowledge that made them more aware of the importance of self-care behaviors, and might facilitate timely help seeking. This science-driven design process will lay the groundwork for further development and evaluation of this solution before its implementation in health care systems. Furthermore, a detailed description of the HCD process used in this research will help guide the development and evaluation of future digital health solutions across a range of disease use cases.

## Appendix

Multimedia Appendix 1 Topic guides for the qualitative semistructured interviews.

Multimedia Appendix 2 Determinants of self-care behaviours.

Multimedia Appendix 3 Behaviour change technique mapping.

Multimedia Appendix 4 App design wireframe.

Multimedia Appendix 5 Mapping of behavior change techniques to the specific educational content of the system and system requirements.

Multimedia Appendix 6 Baseline data window computation method.

## Abbreviations

BCT: behavior change technique
COPD: chronic obstructive pulmonary disease
CRS: Comfort Rating Scale
DHT: digital health technology
EHFScBS: European Heart Failure Self-care Behavior Scale
HCD: human-centered design
HF: heart failure
MLwHFQ: Minnesota Living with Heart Failure Questionnaire
SUS: System Usability Scale
TDF: Theoretical Domains Framework
WTMS: Wearable Technology Motivation Scale
