# Supplementary material for: Human-Centered Design of a Digital Health Tool to Promote Effective Self-care in Patients With Heart Failure: Mixed Methods Study
Source: JMIR Form Res. 2022 May 10;6(5):e34257. doi: 10.2196/34257 (PMC9131139; doi:10.2196/34257)
Supplement: Multimedia Appendix 1 [file formative_v6i5e34257_app1.docx]

# Supplemental Appendix 1

# Study 1

Table 1: The topic guide for the step-1 qualitative study (empathise)

| Topic | Discussion Points and Questions |
| --- | --- |
| Condition Experience | 1. Discuss your experience of HF. 2. Discuss the types of things do you do to help manage your condition. 3. Talk me through how you have done this in the last week. 4. Discuss how you feel about the self-management of your condition. 5. Discuss the last time you went to your general practitioner or went to hospital. |
| Healthcare Pathway Experience | 1. Discuss the things you do when you feel that your condition is getting worse. 2. Tell me how you feel about the care that you have received for your HF. 3. Tell me a bit about the education and advice you have received for the management of your condition. 4. Discuss what you would like to see included in a HF education programme. |
| Technology | 1. I see you own X technology, tell me what you use these devices for. 2. Discuss the problems you have using these devices. 3. Talk me through how you have tried to overcome these problems. |
| Health Data and DHT | 1. Tell me how you think technology could play a part in the management of your condition in your home. 2. Discuss how you would feel about using a digital health technology for example an activity tracker, Bluetooth weighing scales, a blood pressure monitor, ECG and a HF related smartphone app to generate health data/information about yourself. 3. Discuss how collecting health information would impact how you manage your condition. 4. If the health information from these devices could be sent to your doctor on a regular basis, where they can see the information you are collecting, discuss how you would feel about that. 5. Tell me a bit about how you think your HCP could use your health data/information to manage you HF. 6. Discuss how would you might fit this into your day. |

## Study 2

## Topic Guide

Table 2: The topic guide for the step-2 mixed-methods study (test)

| **Topic** | **Discussion Points and Questions** |
| --- | --- |
| Experience with system | 1. First impressions 2. Did they change – why? 3. Thoughts/emotions – why? 4. Likes/Dislikes 5. Ease of use – why? 6. Any difficulties – what and why? 7. Feedback    1. Understandable? Why/why not? 8. Content    1. Preferred style    2. Understanding    3. Reminders |
| Impact on day-to-day life | 1. What changed from normal routine?    1. May need to go into what is normal for them? 2. Impact on behavior – why/why not?    1. Medication adherence    2. Physical activity    3. Sleep    4. Weight 3. Altered decision making? 4. Time spent on monitoring 5. Interaction with others in life? 6. Awareness of the Fitbit/interaction with clothes etc. |
| Impact on condition perceptions | 1. Confidence?    1. In themselves/their ability    2. Knowledge of condition    3. Understanding signs and symptoms 2. Motivation 3. Privacy concerns 4. Impact on healthcare relationship 5. Being monitored remotely 6. Any remaining uncertainties |
| Changes to system | 1. Features 2. Ease of use 3. Content of app 4. The Fitbit itself 5. Understanding of condition 6. What are remaining needs? 7. Support of family? |
