# Supplementary material for: Human-Centered Design of a Digital Health Tool to Promote Effective Self-care in Patients With Heart Failure: Mixed Methods Study
Source: JMIR Form Res. 2022 May 10;6(5):e34257. doi: 10.2196/34257 (PMC9131139; doi:10.2196/34257)
Supplement: Multimedia Appendix 2 [file formative_v6i5e34257_app2.docx]

# Supplemental appendix 2

Determinants of self-care behaviours.

| **Identified barrier/facilitator** | **Determinant of self-care** | **TDF Domain** |
| --- | --- | --- |
| Lack of understanding about basic elements of the nature of HF | Integrating self-care into daily life | Knowledge |
| Apparent misattribution of HF symptoms to other conditions, age or medications | Early detection of signs and symptoms | Knowledge  Beliefs about consequences |
| Lack of understanding around what symptoms of flare-up are | Early detection of signs and symptoms | Knowledge |
| Lack of understanding regarding meaning of, or how to react to, any changes in condition or outcomes (timely help seeking) | Early detection of signs and symptoms | Knowledge  Skills  Beliefs about capabilities  Memory, attention and decision processes  Behavioural regulation |
| Poor understanding regarding what self-care behaviours are important in HF | Integrating self-care into daily life | Knowledge  Beliefs about capabilities |
| Self-reliance for daily weighing | Integrating self-care into daily life | Knowledge  Skills  Beliefs about capabilities  Memory, attention and decision processes  Behavioural regulation |
| Self-reliance for physical activity levels | Integrating self-care into daily life | Knowledge  Skills  Beliefs about capabilities  Memory, attention and decision processes  Behavioural regulation |
| Self-reliance for help seeking | Integrating self-care into daily life | Knowledge  Skills  Beliefs about capabilities  Memory, attention and decision processes  Behavioural regulation |
| Self-reliance for fluid restriction | Integrating self-care into daily life | Knowledge  Skills  Beliefs about capabilities  Memory, attention and decision processes  Behavioural regulation |
| Integration of self-care with normal life patterns (ADLs). | Integrating self-care into daily life | Skills  Memory, attention and decision processes  Behavioural regulation |
| Support required for medication adherence | Integrating self-care into daily life  Caregiver support skills | Skills  Social influences  Beliefs about capabilities  Environmental context and resources  Memory, attention and decision processes  Behavioural regulation |
| Support required for diet/sodium | Integrating self-care into daily life  Caregiver support skills | Skills  Beliefs about capabilities  Social influences  Environmental context and resources  Memory, attention and decision processes  Behavioural regulation |
| Potential lack of informal carer knowledge or support | Caregiver support skills | Social influences  Environmental context and resources |
| Lack of equipment (scales, heart rate monitor, blood pressure etc) | Integrating self-care into daily life | Environmental context and resources  Behavioural regulation |
| Need for formative experiences to help understanding meaning of changes in signs/symptoms | Early detection of signs and symptoms | Beliefs about consequences  Reinforcement  Emotion |
| Help healthcare providers make informed decisions. | Social support | Social influences  Environmental context and resources  Social role and identity |
| Reliance on informal healthcare givers | Caregiver support skills | Social influences  Beliefs about capabilities  Environmental context and resources |
| Avoidance or low awareness of severity of their HF condition | Early detection of signs and symptoms | Beliefs about consequences |

Table 4: Behaviour Change Technique mapping

| **Determinants of behaviour** | **TDF domain** | **Intervention function** | **Behaviour change techniques as per BCTTv1** |
| --- | --- | --- | --- |
| Integrating self-care into daily life | Knowledge | Education | 2.1 monitoring of behaviour without feedback  2.2 feedback on behaviour  2.3 self-monitoring of behaviour  2.6 biofeedback  2.5 monitoring of outcome of behaviour without feedback  2.7 feedback on outcome of behaviour  3.1 social support  4.1 instruction on how to perform the behaviour  5.1 information on consequences of behaviour  5.3 information on social and environmental consequence  7.1 prompts/cue  8.1 behavioural practice  8.3 habit formation  9.1 credible source  11.1 pharmacological support  12.5 adding objects to the environment  15.1 verbal persuasion about capability |
|  | Skills | Training |  |
|  | Memory, attention and decision processes | Environmental Restructuring and Restrictions |  |
|  | Behavioural regulation | Enablement |  |
|  | Environmental context and resources  Reinforcement  Beliefs about consequences  Beliefs about capabilities  Social influences | Persuasion |  |

| Early detection of signs and symptoms | Knowledge | Education | 2.2 feedback on behaviour  2.3 self-monitoring of behaviour  2.6 biofeedback  2.7 feedback on outcome of behaviour  3.1 social support  4.1 instruction on how to perform the behaviour  5.1 information on consequences of behaviour  5.3 information on social and environmental consequence  7.1 prompts/cues  8.1 behavioural practice  8.3 habit formation  9.1 credible source  12.5 adding objects to the environment  15.1 verbal persuasion about capability |
| --- | --- | --- | --- |
|  | Skills | Training |  |
|  | Beliefs about capabilities | Enablement |  |
|  | Reinforcement  Emotion  Memory, attention and decision processes | Environmental Restructuring  Persuasion |  |
|  | Behavioural regulation |  |  |
|  | Knowledge  Beliefs about consequences |  |  |
| Caregiver support skills | Social influences | N/A for this intervention | N/A for this intervention |
|  | Beliefs about capabilities |  |  |
|  | Environmental context and resources |  |  |
|  | Social influences  Memory, attention and decision processes  Behavioural regulation |  |  |
| Social support | Social influences | Enablement | 3.1 social support  7.1 prompts/cues |
|  | Environmental context and resources | Persuasion |  |
|  | Social role and identity |  |  |
|  |  |  |  |
