# Supplementary material for: Human-Centered Design of a Digital Health Tool to Promote Effective Self-care in Patients With Heart Failure: Mixed Methods Study
Source: JMIR Form Res. 2022 May 10;6(5):e34257. doi: 10.2196/34257 (PMC9131139; doi:10.2196/34257)
Supplement: Multimedia Appendix 3 [file formative_v6i5e34257_app3.docx]

# Supplemental appendix 3

Behaviour change technique mapping

| **Determinants of behaviour** | **TDF domain** | **Intervention function** | **Behaviour change techniques as per BCTTv1** |
| --- | --- | --- | --- |
| Integrating self-care into daily life | Knowledge | Education | 2.1 monitoring of behaviour without feedback  2.2 feedback on behaviour  2.3 self-monitoring of behaviour  2.6 biofeedback  2.5 monitoring of outcome of behaviour without feedback  2.7 feedback on outcome of behaviour  3.1 social support  4.1 instruction on how to perform the behaviour  5.1 information on consequences of behaviour  5.3 information on social and environmental consequence  7.1 prompts/cue  8.1 behavioural practice  8.3 habit formation  9.1 credible source  11.1 pharmacological support  12.5 adding objects to the environment  15.1 verbal persuasion about capability |
|  | Skills | Training |  |
|  | Memory, attention and decision processes | Environmental Restructuring and Restrictions |  |
|  | Behavioural regulation | Enablement |  |
|  | Environmental context and resources  Reinforcement  Beliefs about consequences  Beliefs about capabilities  Social influences | Persuasion |  |

| Early detection of signs and symptoms | Knowledge | Education | 2.2 feedback on behaviour  2.3 self-monitoring of behaviour  2.6 biofeedback  2.7 feedback on outcome of behaviour  3.1 social support  4.1 instruction on how to perform the behaviour  5.1 information on consequences of behaviour  5.3 information on social and environmental consequence  7.1 prompts/cues  8.1 behavioural practice  8.3 habit formation  9.1 credible source  12.5 adding objects to the environment  15.1 verbal persuasion about capability |
| --- | --- | --- | --- |
|  | Skills | Training |  |
|  | Beliefs about capabilities | Enablement |  |
|  | Reinforcement  Emotion  Memory, attention and decision processes | Environmental Restructuring  Persuasion |  |
|  | Behavioural regulation |  |  |
|  | Knowledge  Beliefs about consequences |  |  |
| Caregiver support skills | Social influences | N/A for this intervention | N/A for this intervention |
|  | Beliefs about capabilities |  |  |
|  | Environmental context and resources |  |  |
|  | Social influences  Memory, attention and decision processes  Behavioural regulation |  |  |
| Social support | Social influences | Enablement | 3.1 social support  7.1 prompts/cues |
|  | Environmental context and resources | Persuasion |  |
|  | Social role and identity |  |  |
|  |  |  |  |
