# Supplementary material for: Human-Centered Design of a Digital Health Tool to Promote Effective Self-care in Patients With Heart Failure: Mixed Methods Study
Source: JMIR Form Res. 2022 May 10;6(5):e34257. doi: 10.2196/34257 (PMC9131139; doi:10.2196/34257)

Main Menu

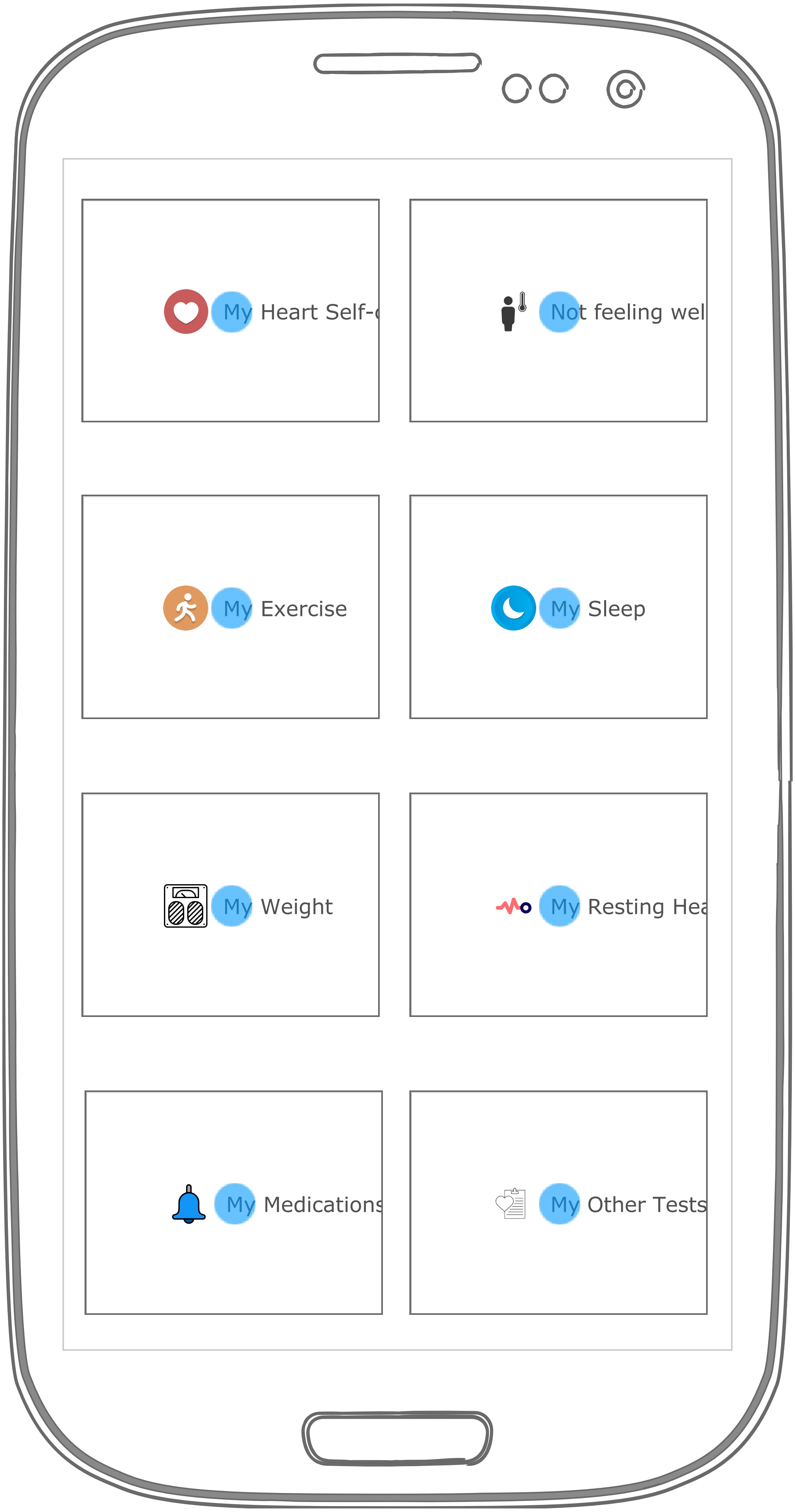
My Heart Self-
Not feeling wel
My Exercise
My Sleep
My Weight
My Resting Hea
My Medications
My Other Tests

Exercise Week

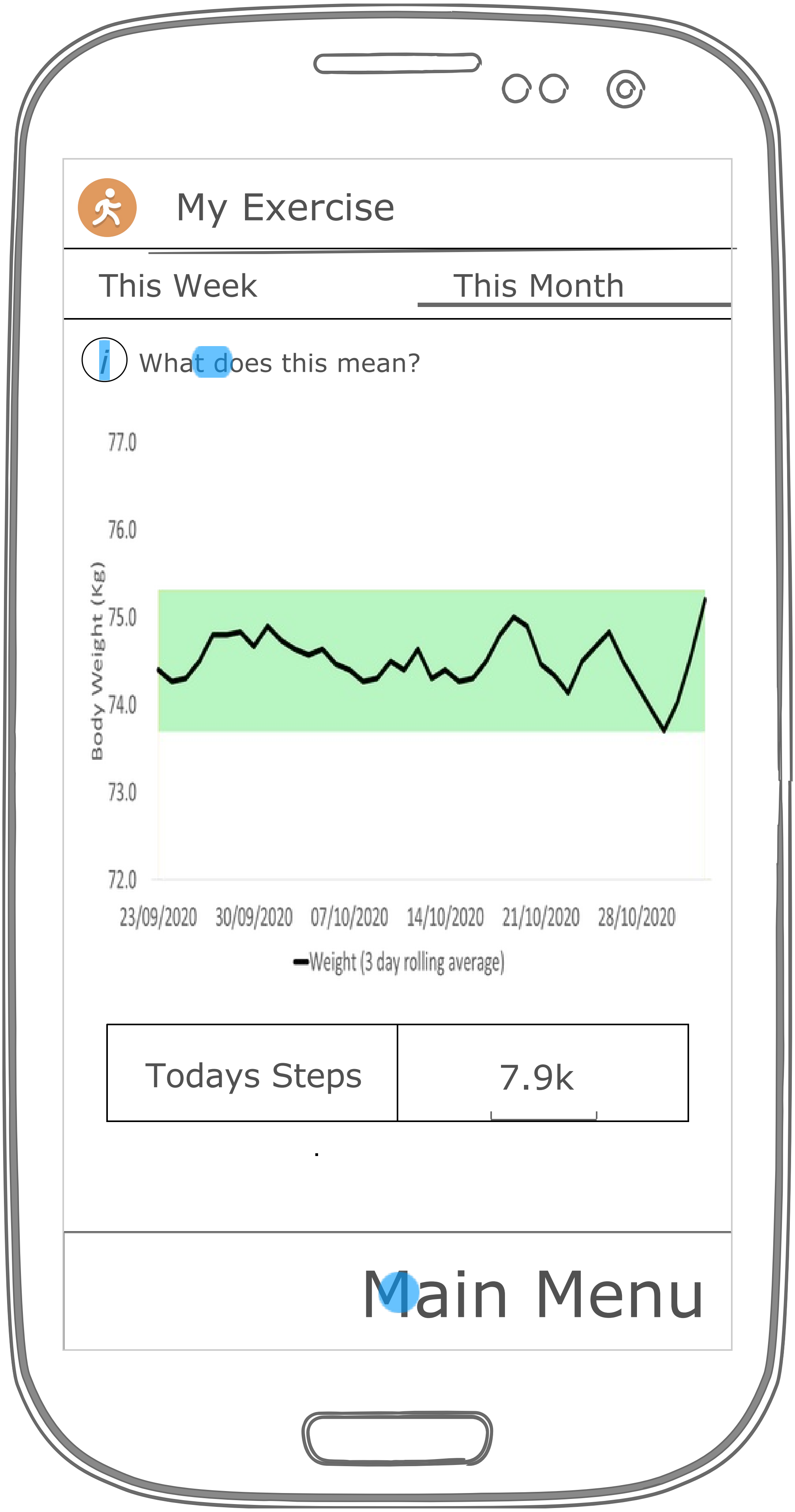

Weight month

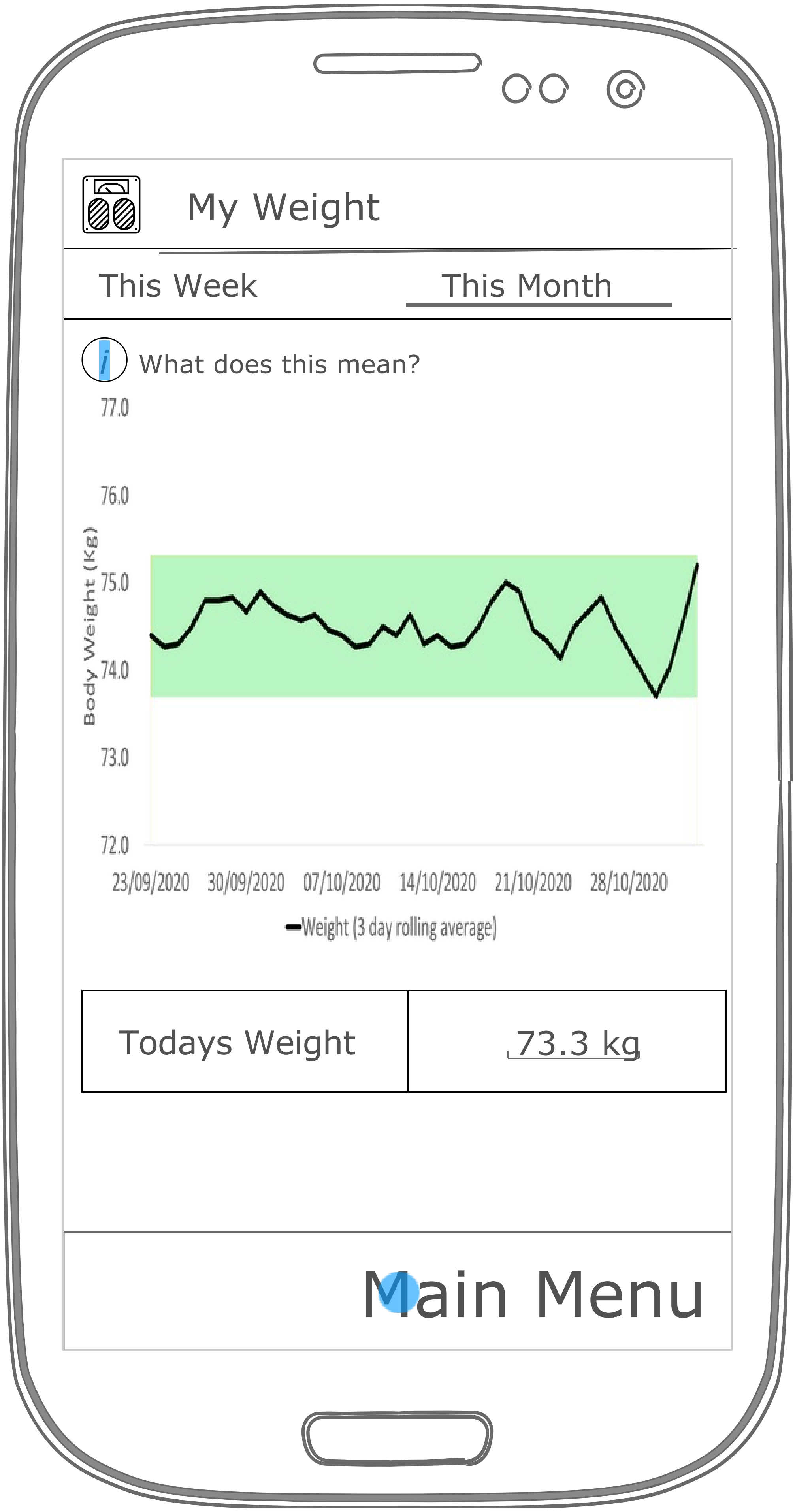

Heart Rate

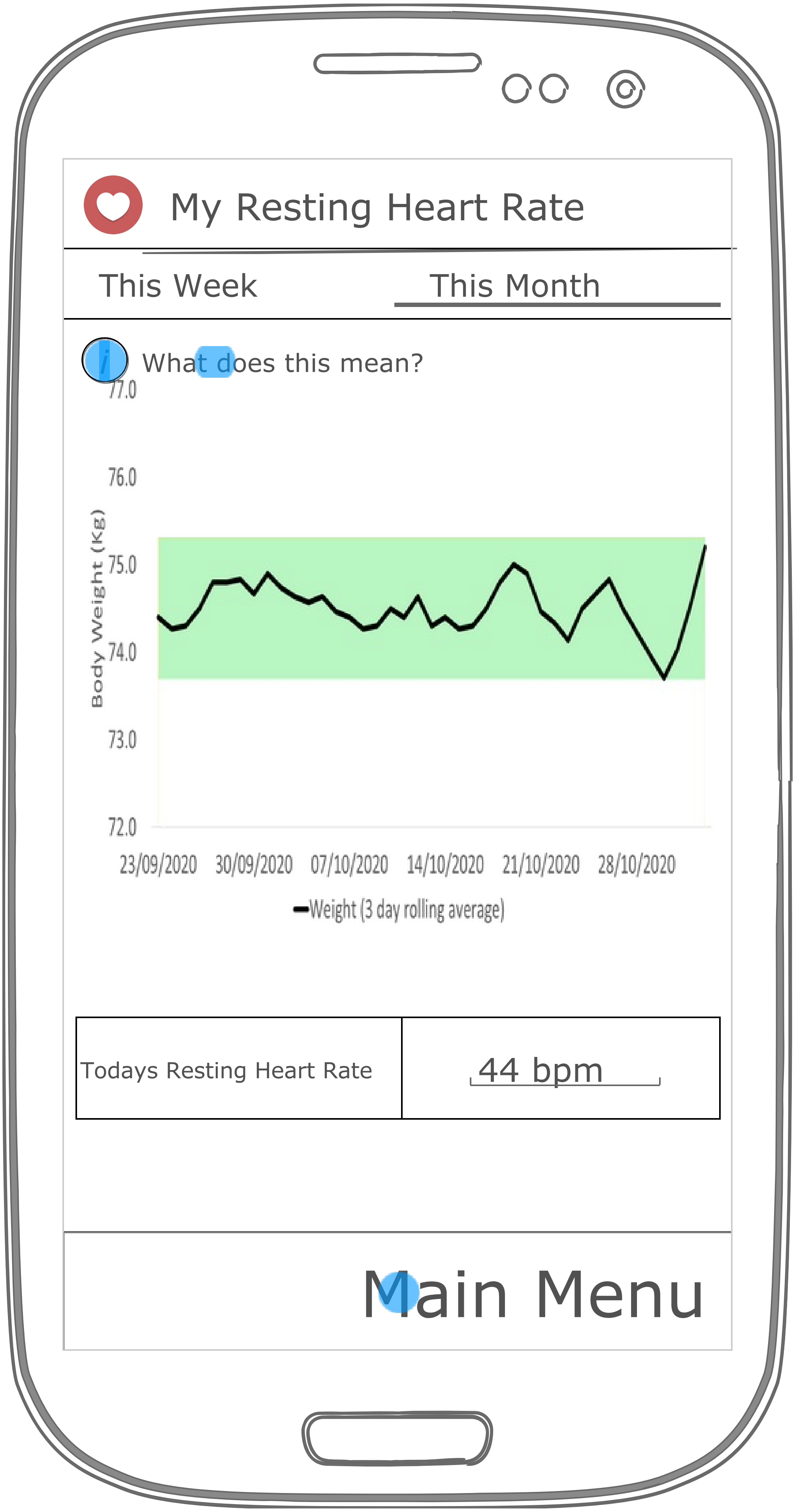
My Resting Heart Rate
This Week
This Month
What does this mean?
77.0
76.0
75.0
74.0
73.0
72.0
Body Weight (Kg)
23/09/2020
30/09/2020
07/10/2020
14/10/2020
21/10/2020
28/10/2020
Weight (3 day rolling average)
Todays Resting Heart Rate
44 bpm
Main Menu

Sleep Week

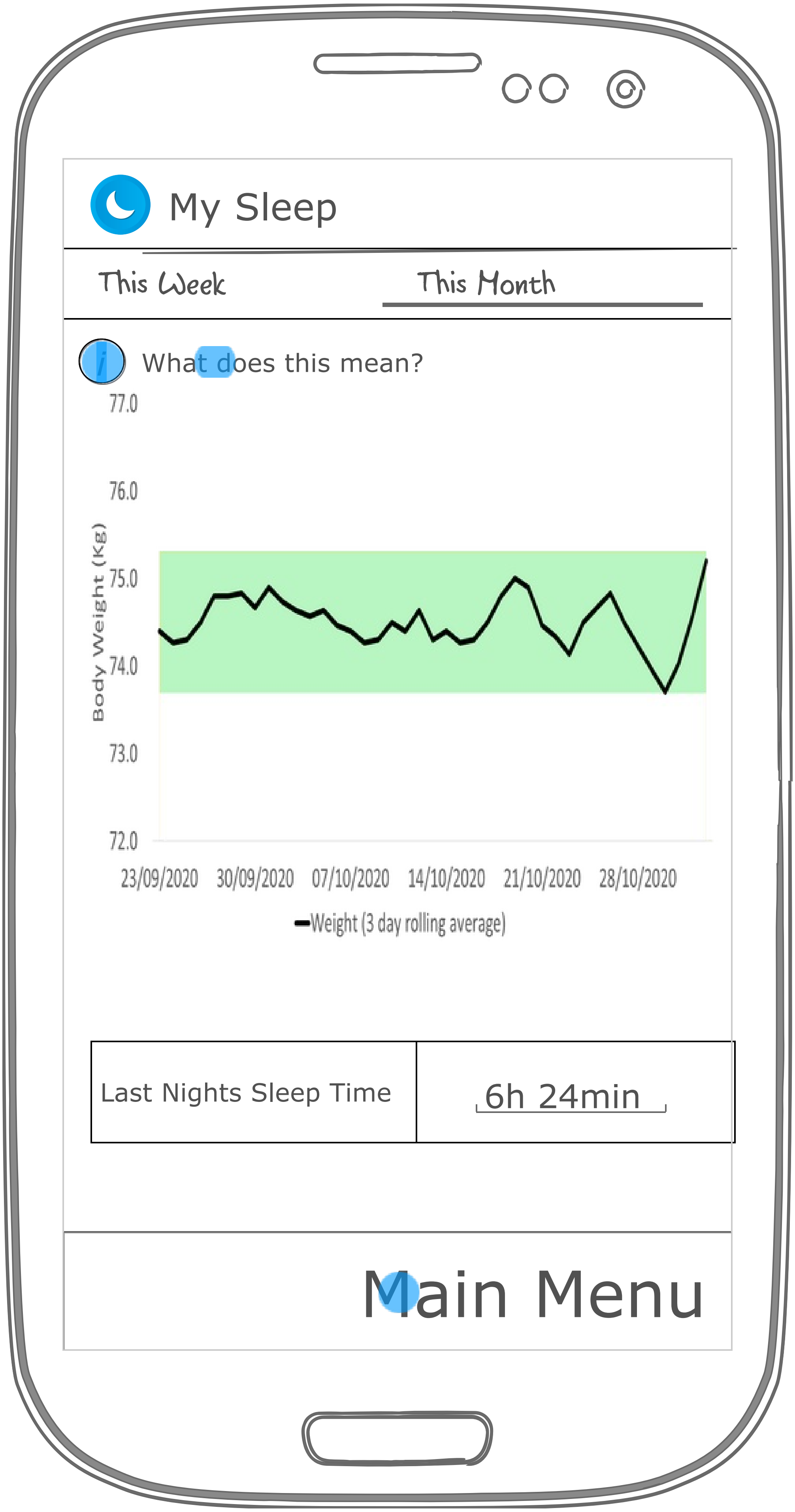
My Sleep
This Week
This Month
What does this mean?
77.0
76.0
75.0
74.0
73.0
72.0
Body Weight (Kg)
23/09/2020
30/09/2020
07/10/2020
14/10/2020
21/10/2020
28/10/2020
Weight (3 day rolling average)
Last Nights Sleep Time
6h 24min
Main Menu

Medication Reminder

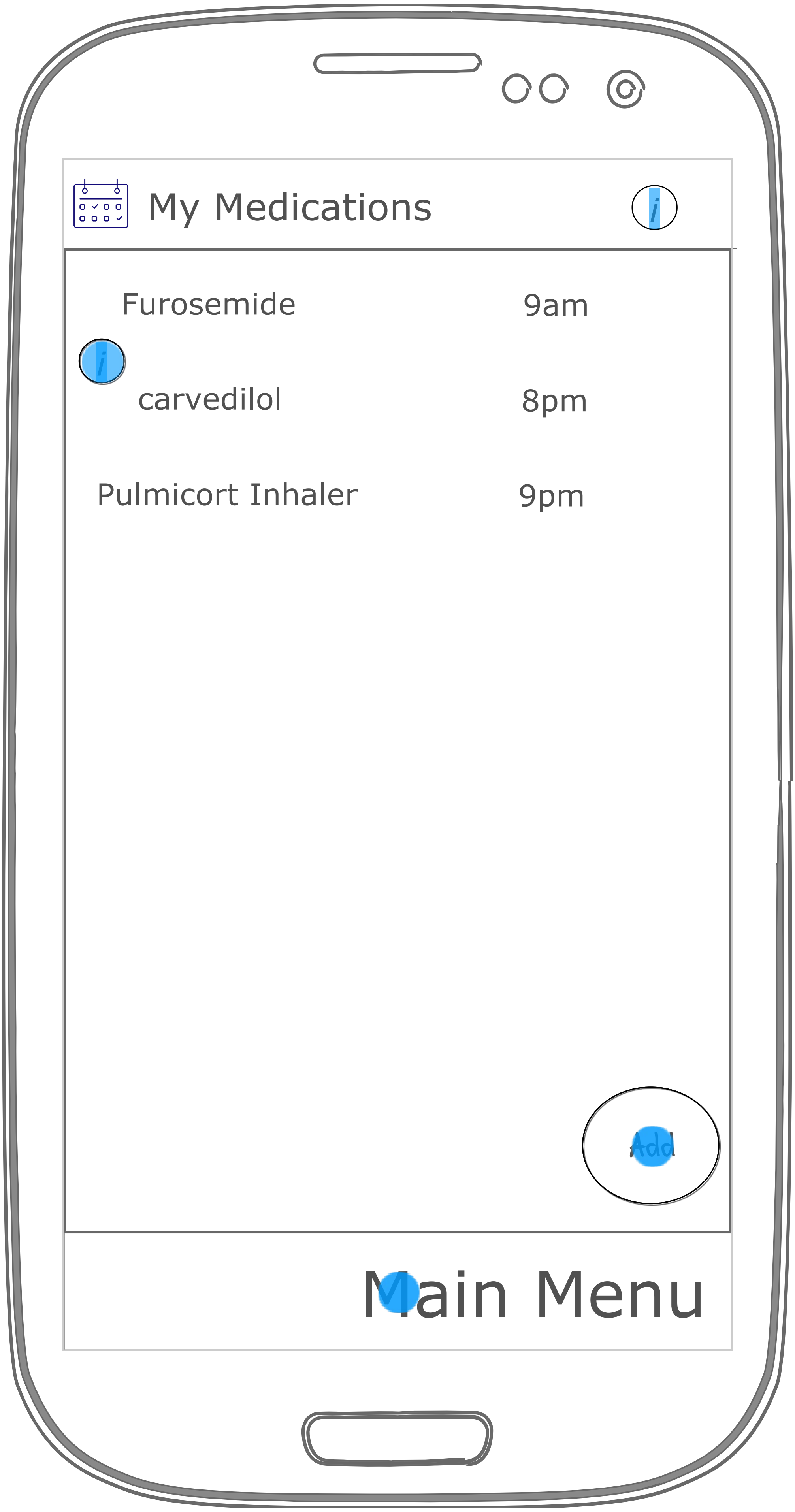

Medication Info

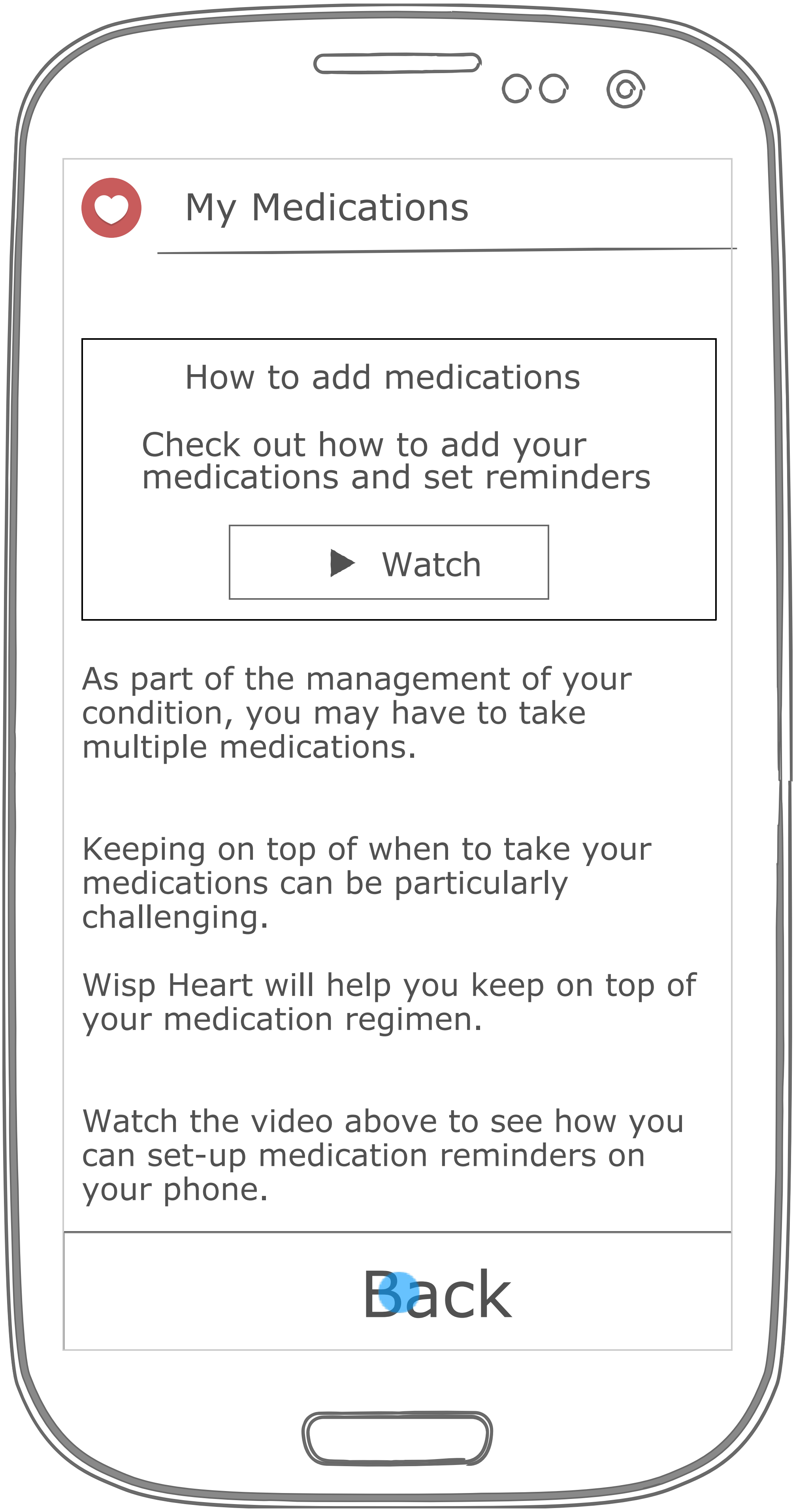

Adding Medication

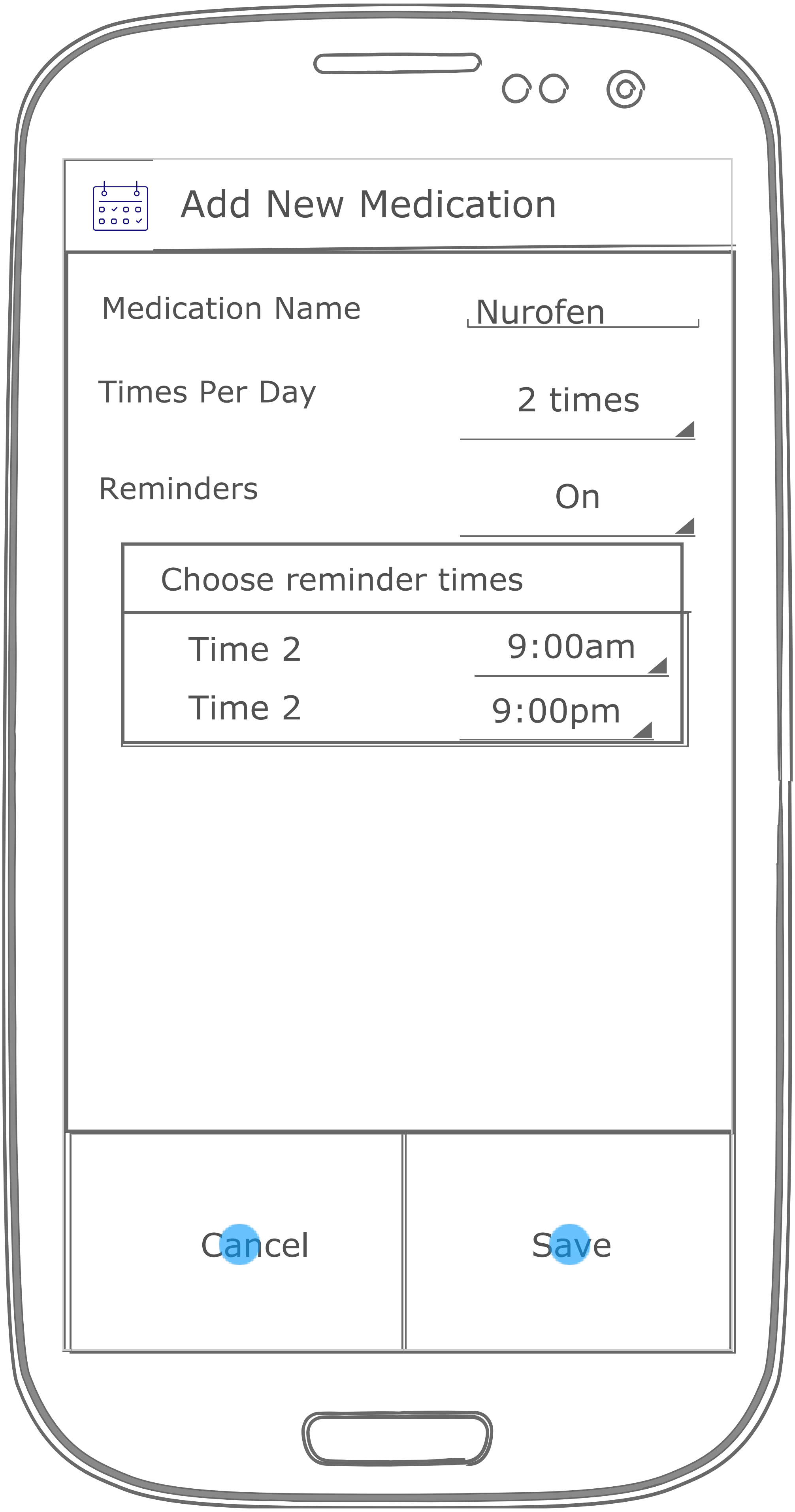

Appointment

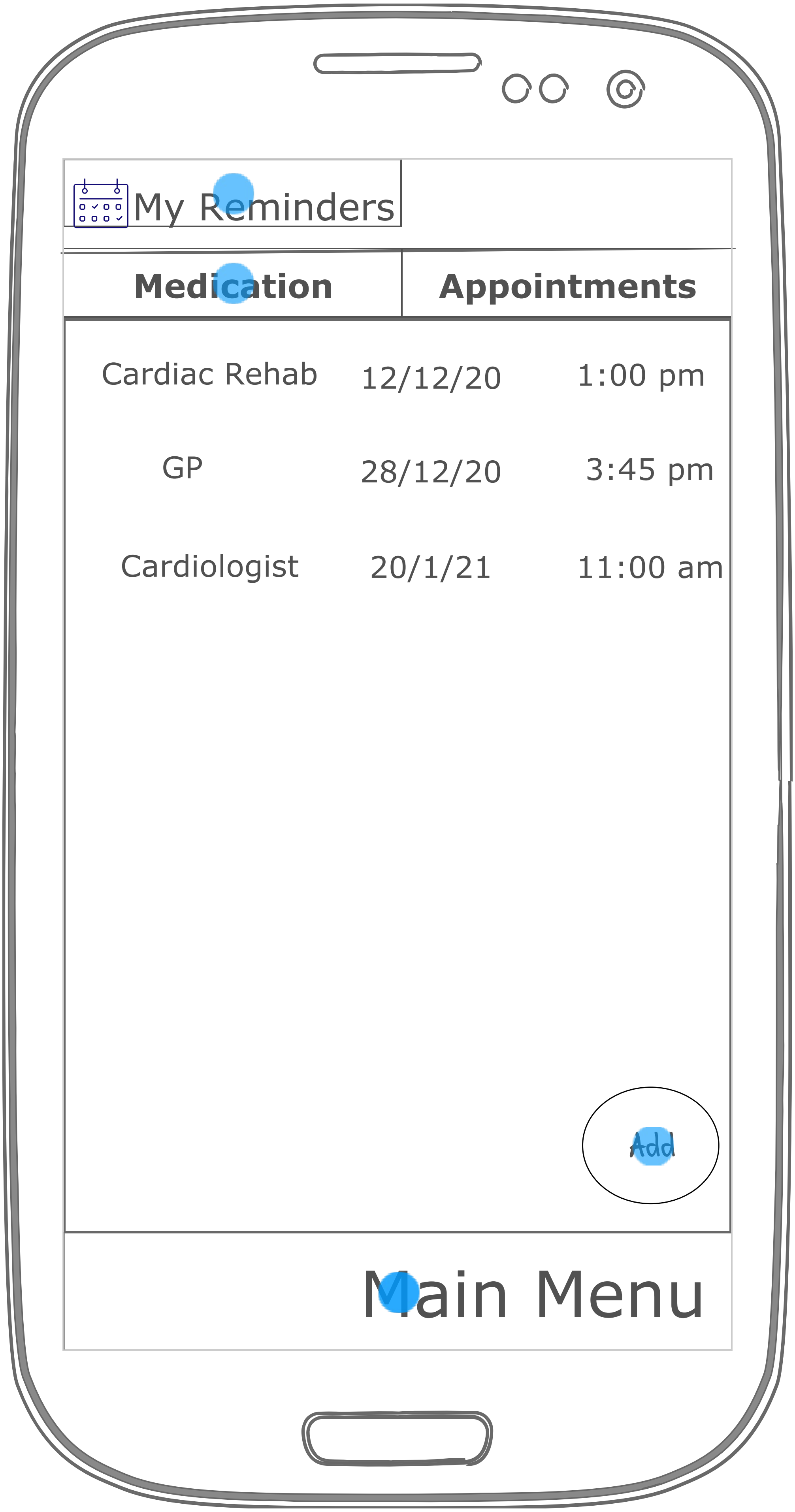

Adding Appointment

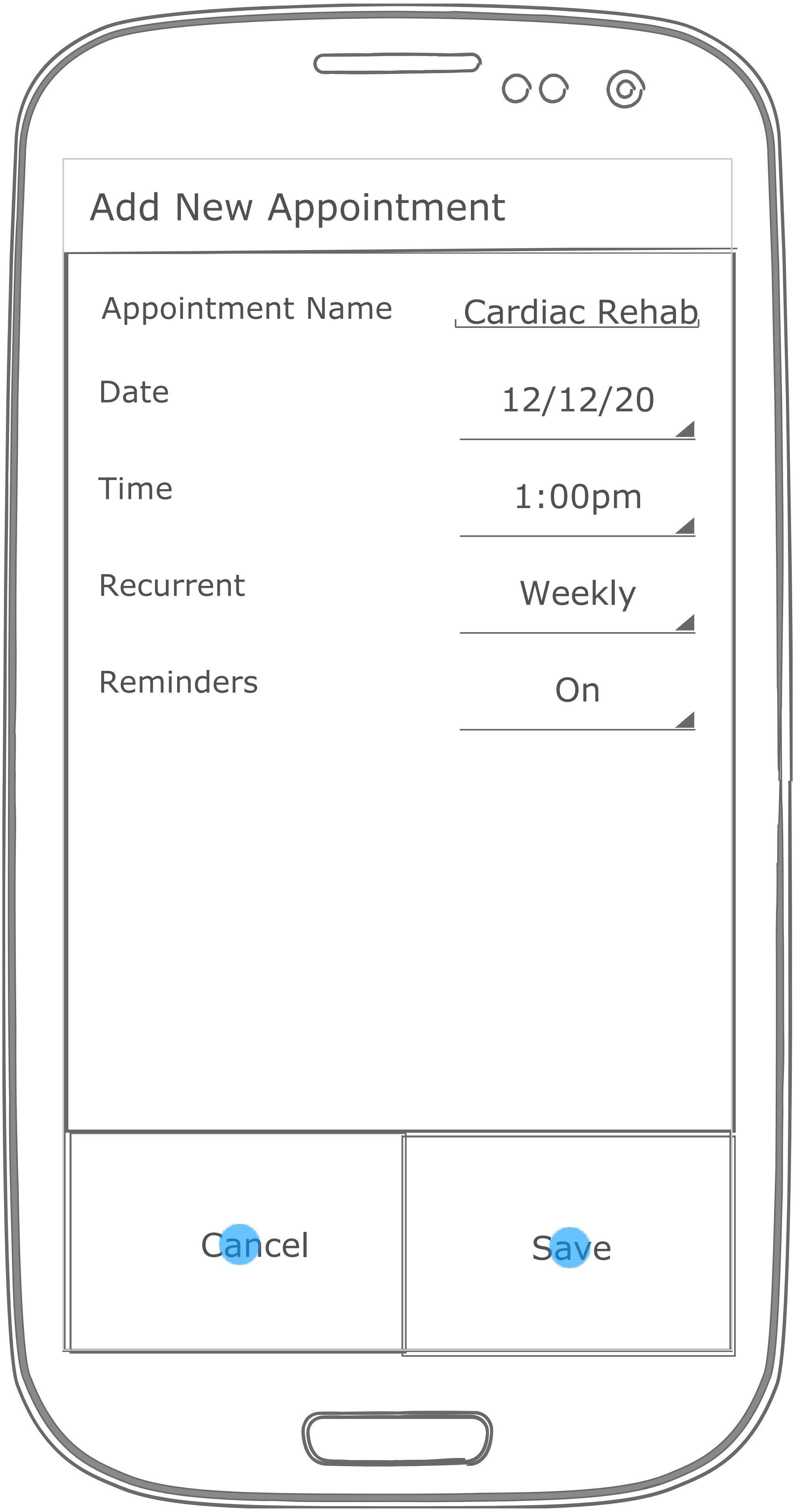

Other Tests

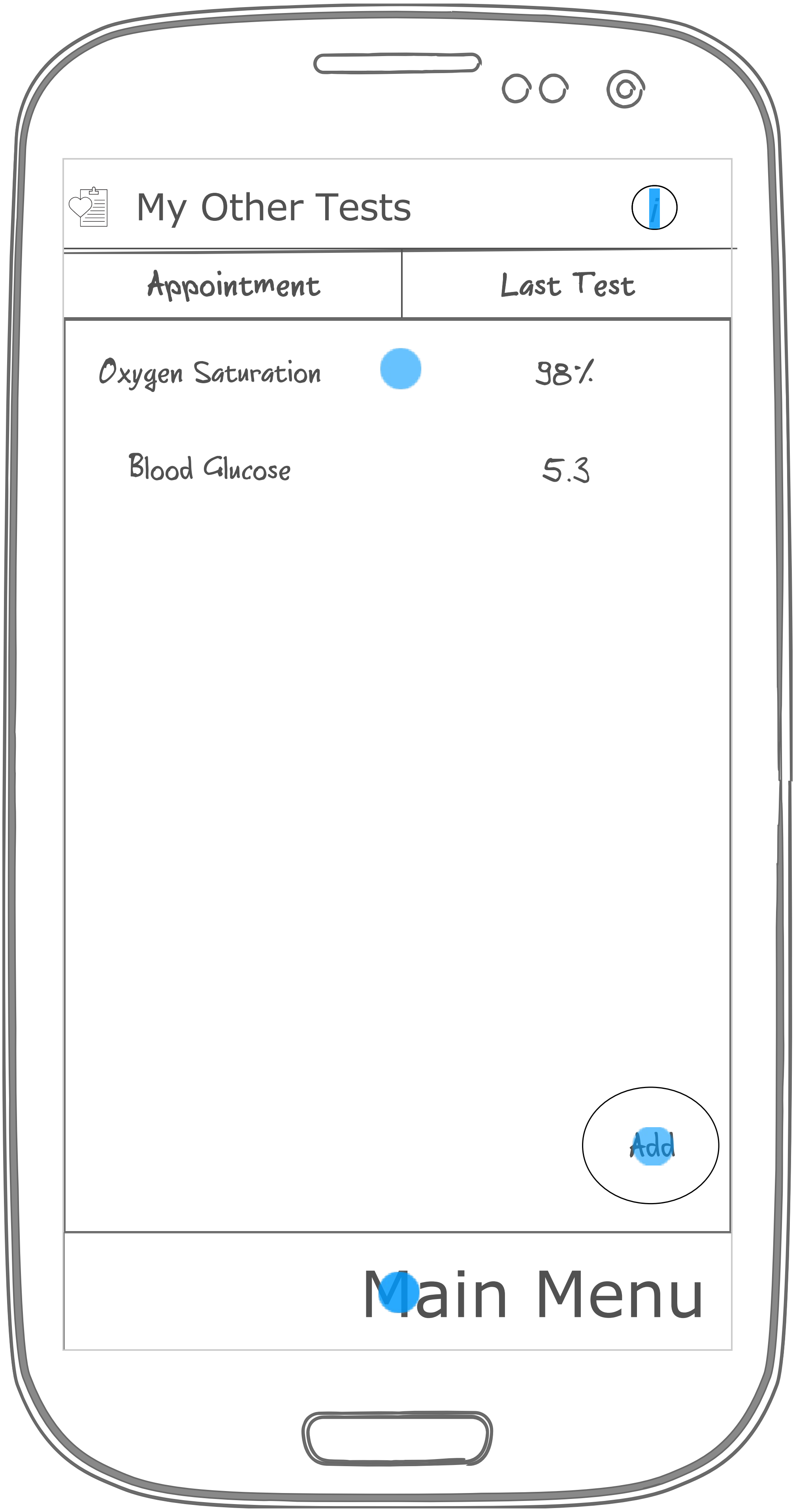
My Other Tests
Appointment
Last Test
Oxygen Saturation
98%
Blood Glucose
5.3
Add
Main Menu

Add Test

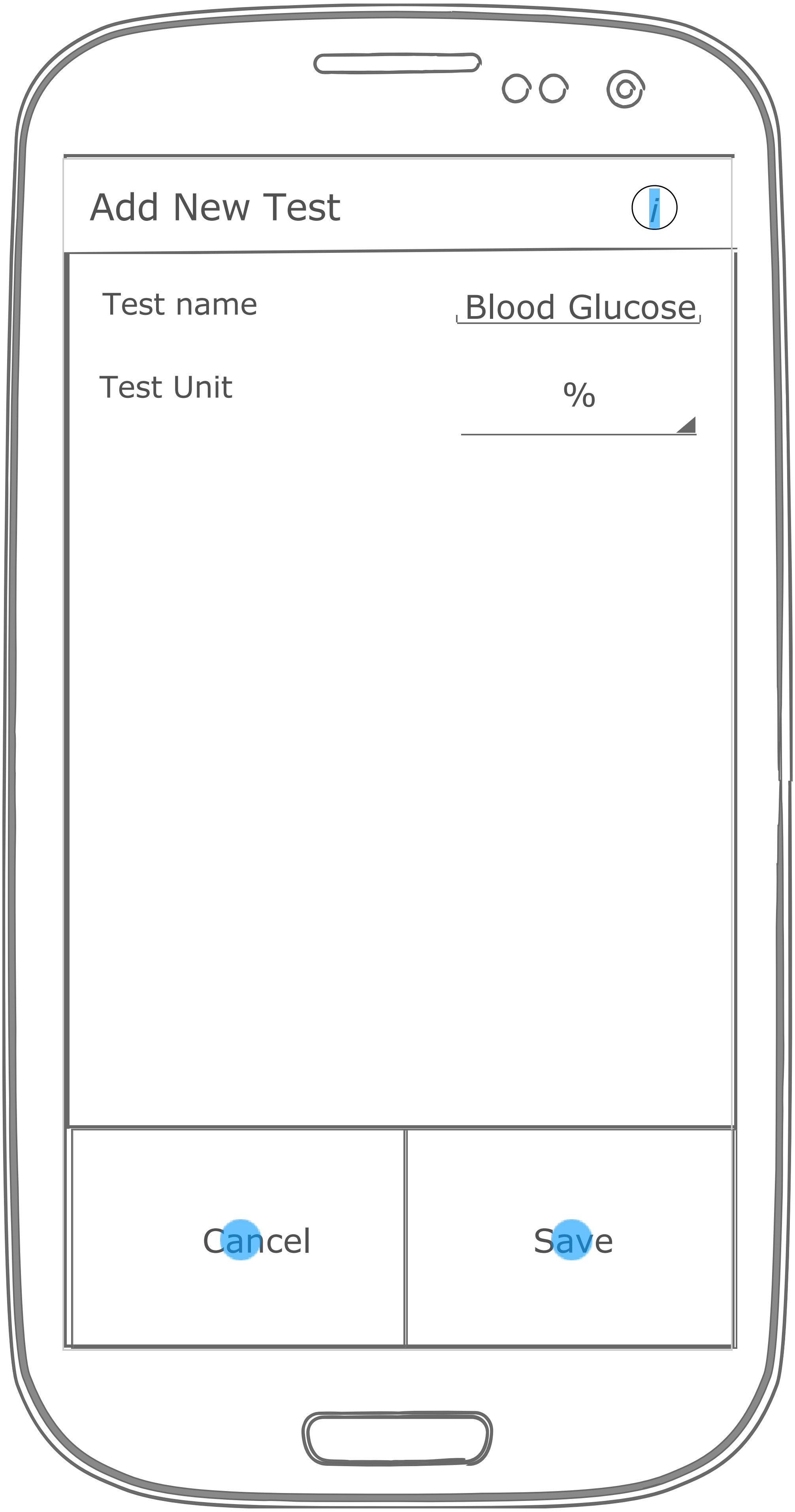

View Test

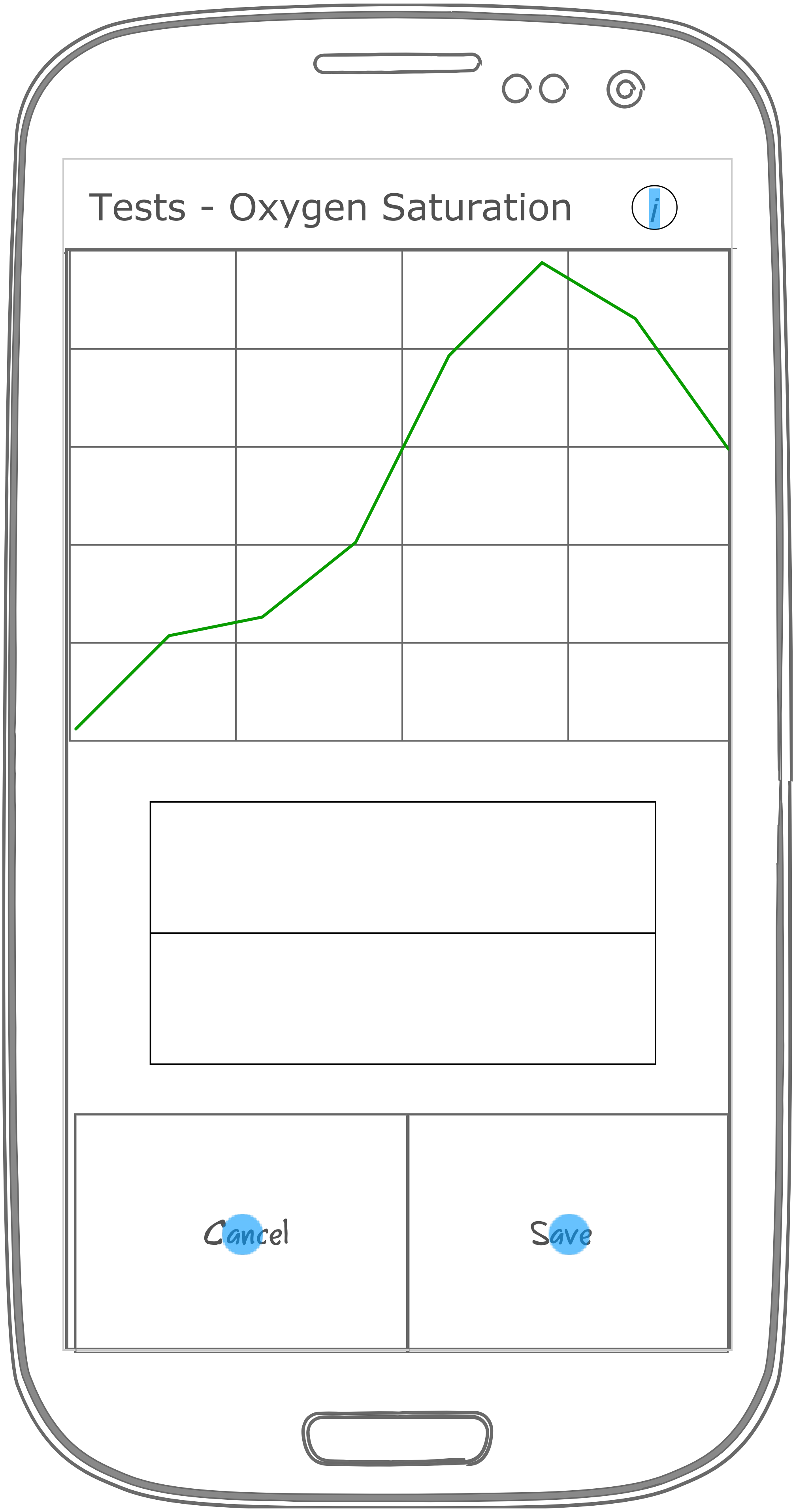

Other Vital info

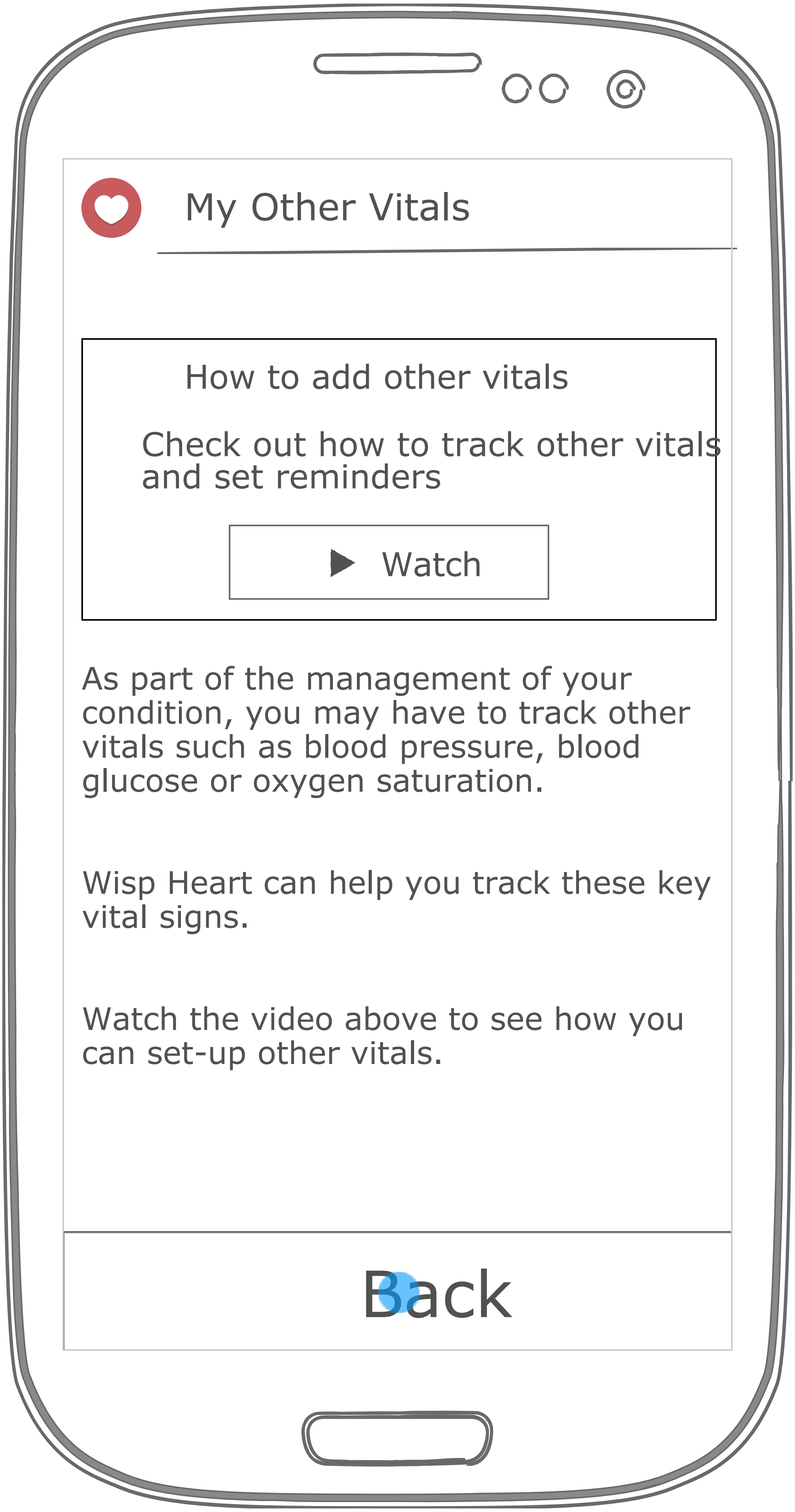

Baseline Questionnaire

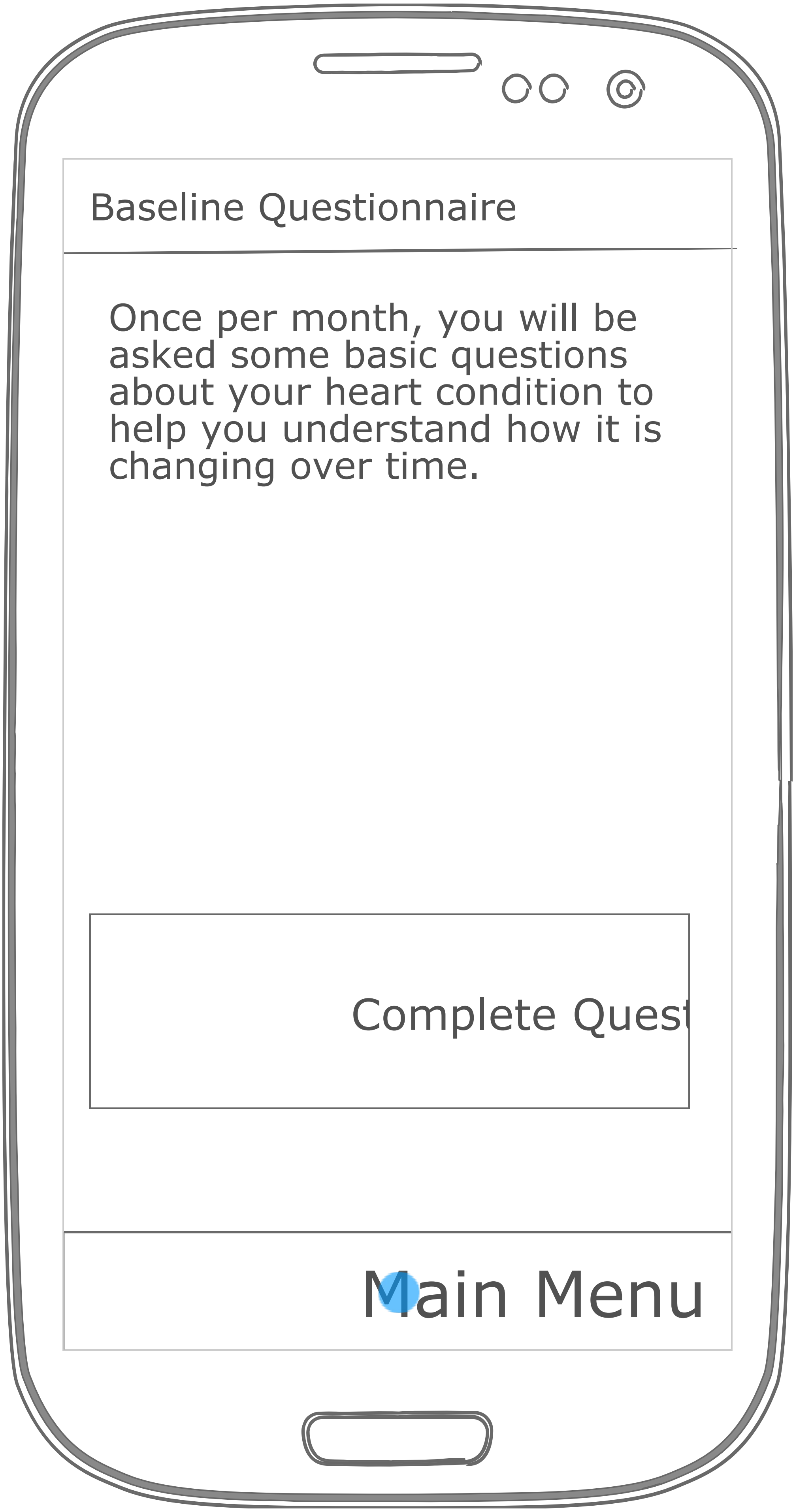

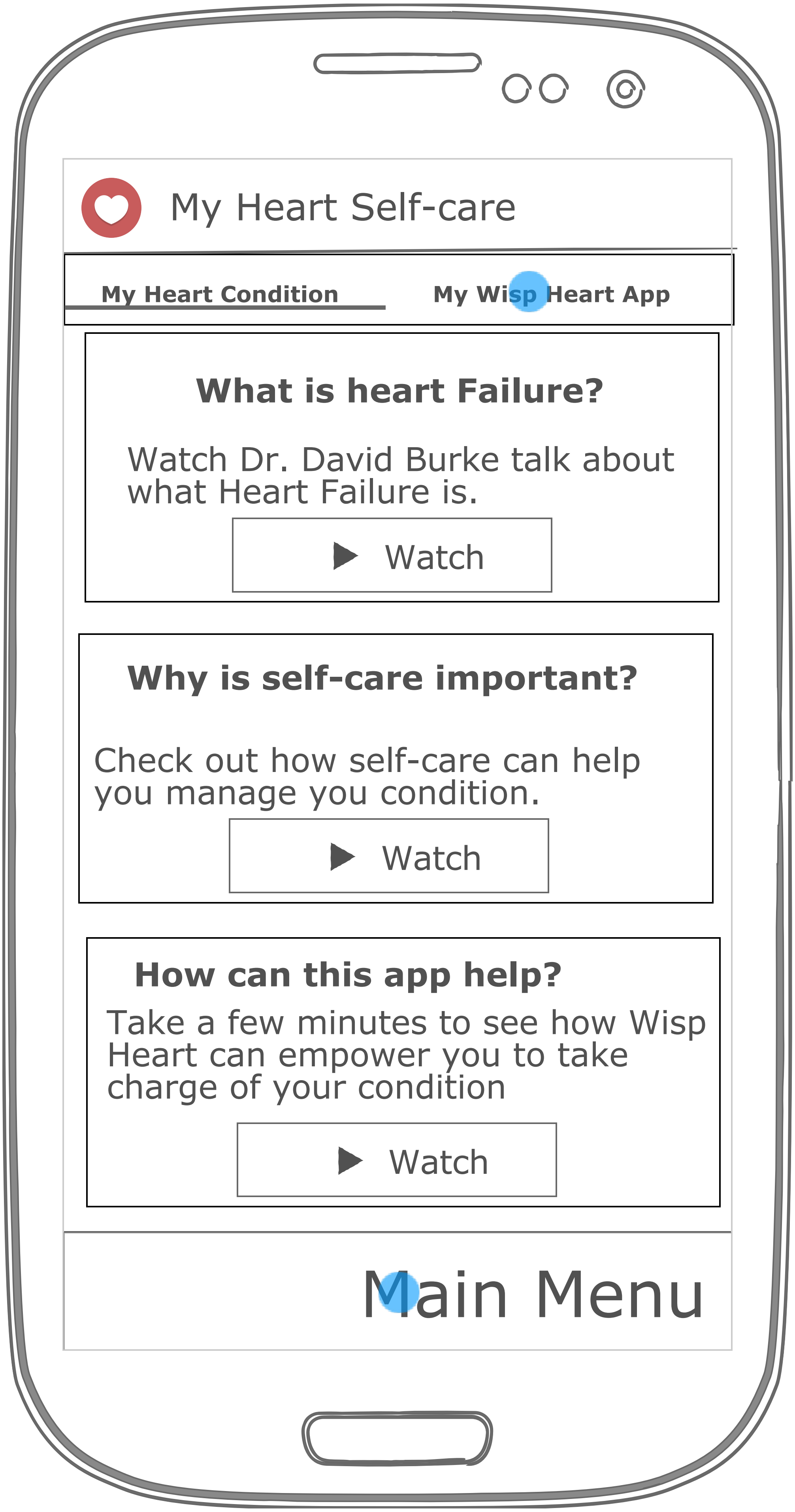
My Heart Self-care
My Heart Condition
My Wisp Heart App
What is heart Failure?
Watch Dr. David Burke talk about what Heart Failure is.
Watch
Why is self-care important?
Check out how self-care can help you manage you condition.
Watch
How can this app help?
Take a few minutes to see how Wisp Heart can empower you to take charge of your condition
Watch
Main Menu

Alternative Education

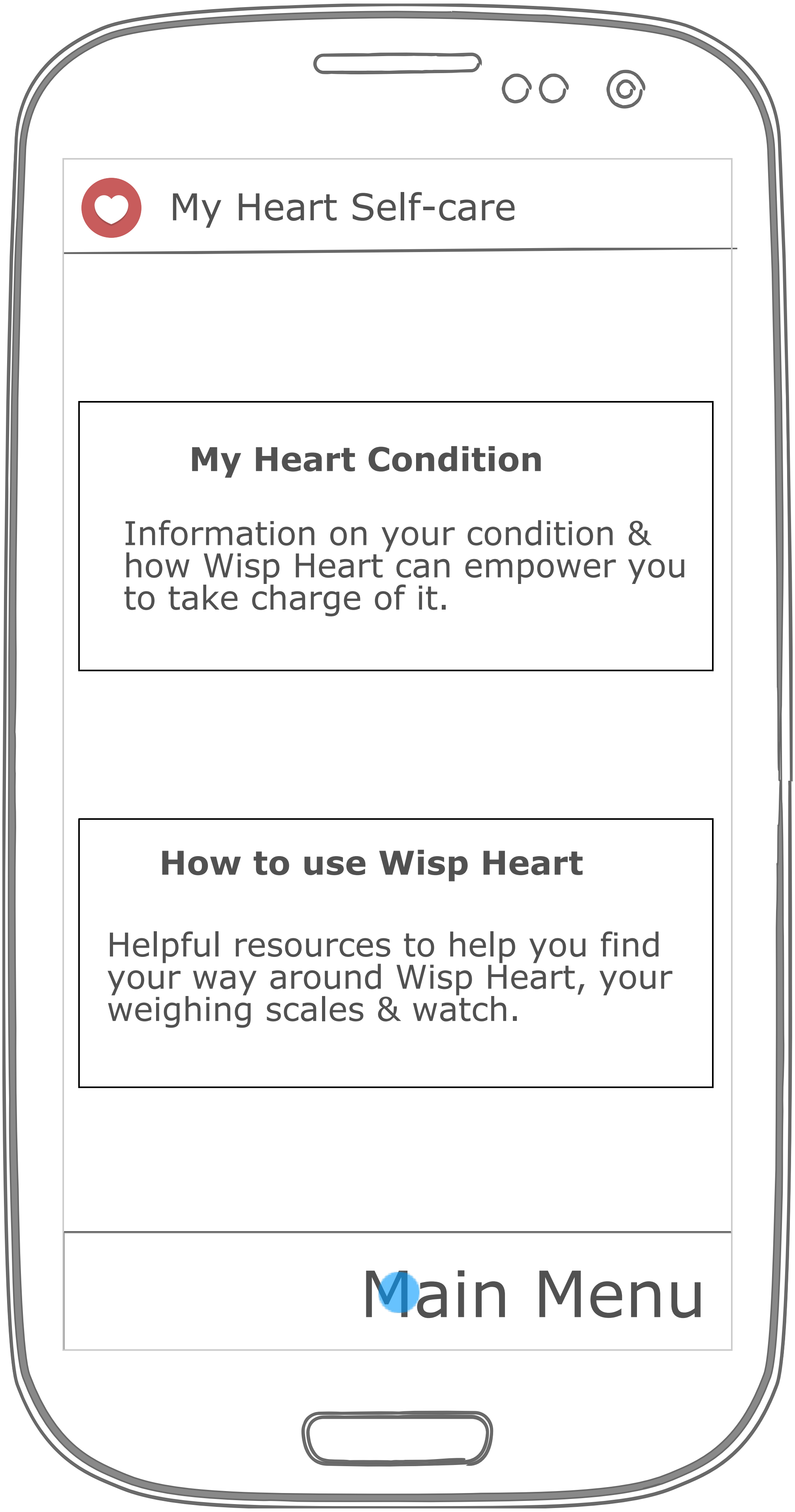

My self-care app

My Heart Self-care

My Heart Condition | **How do I use Wisp Heart**

Set-up and how to videos

▶ Watch

Monitoring your symptoms is a great way to stay aware of any changes that you might not see otherwise. Regular monitoring allows you to live a more active life.

You can help control, your symptoms by following your prescribed treatment plan every day, monitoring yourself routinely for worsening symptoms or sudden changes in key vital signs, and knowing what to do if symptoms worsen.

WispHeart helps you do this. Watch the video to see how.

Main Menu

Why track your weight?

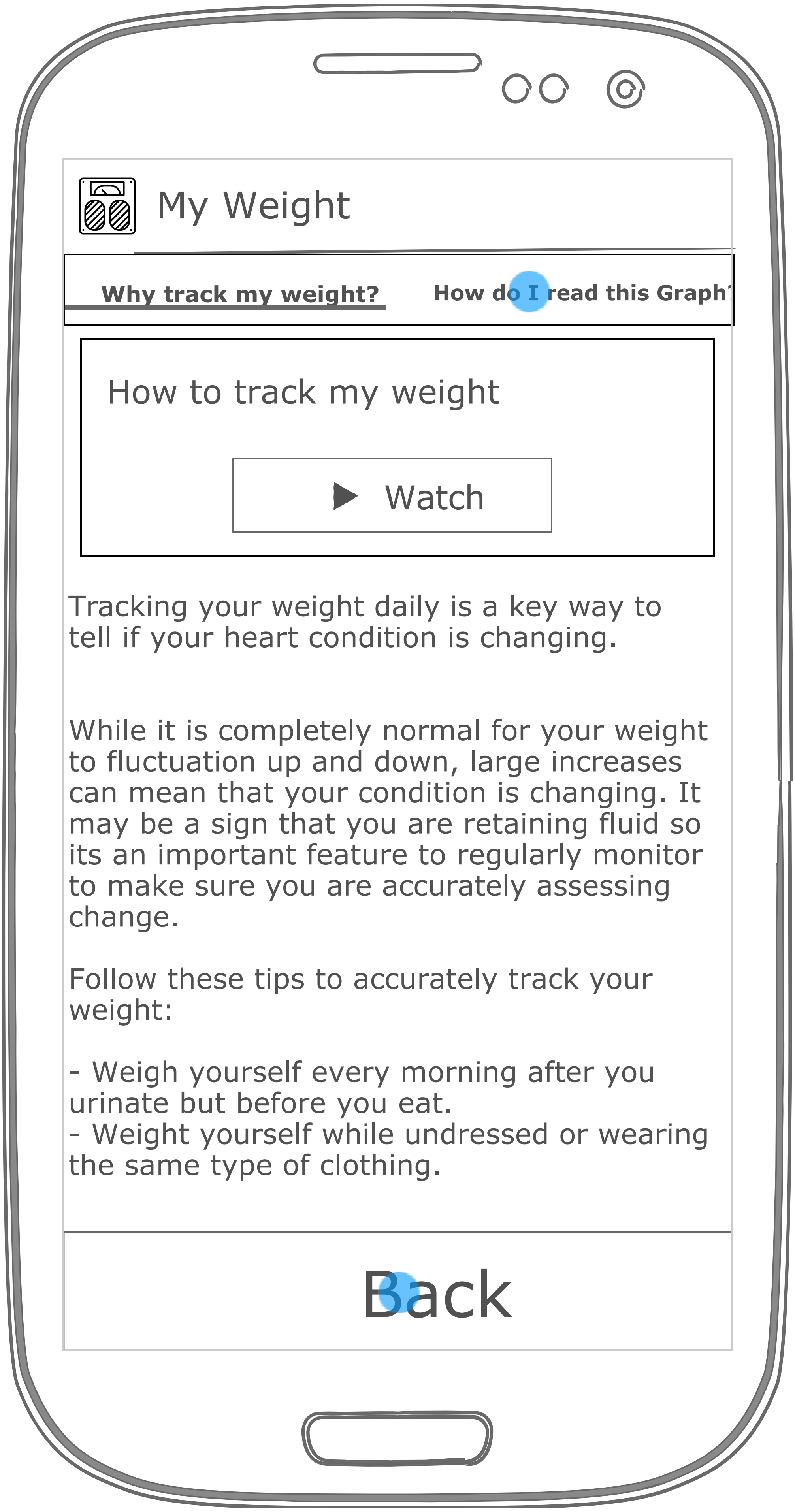

Weight interpretation

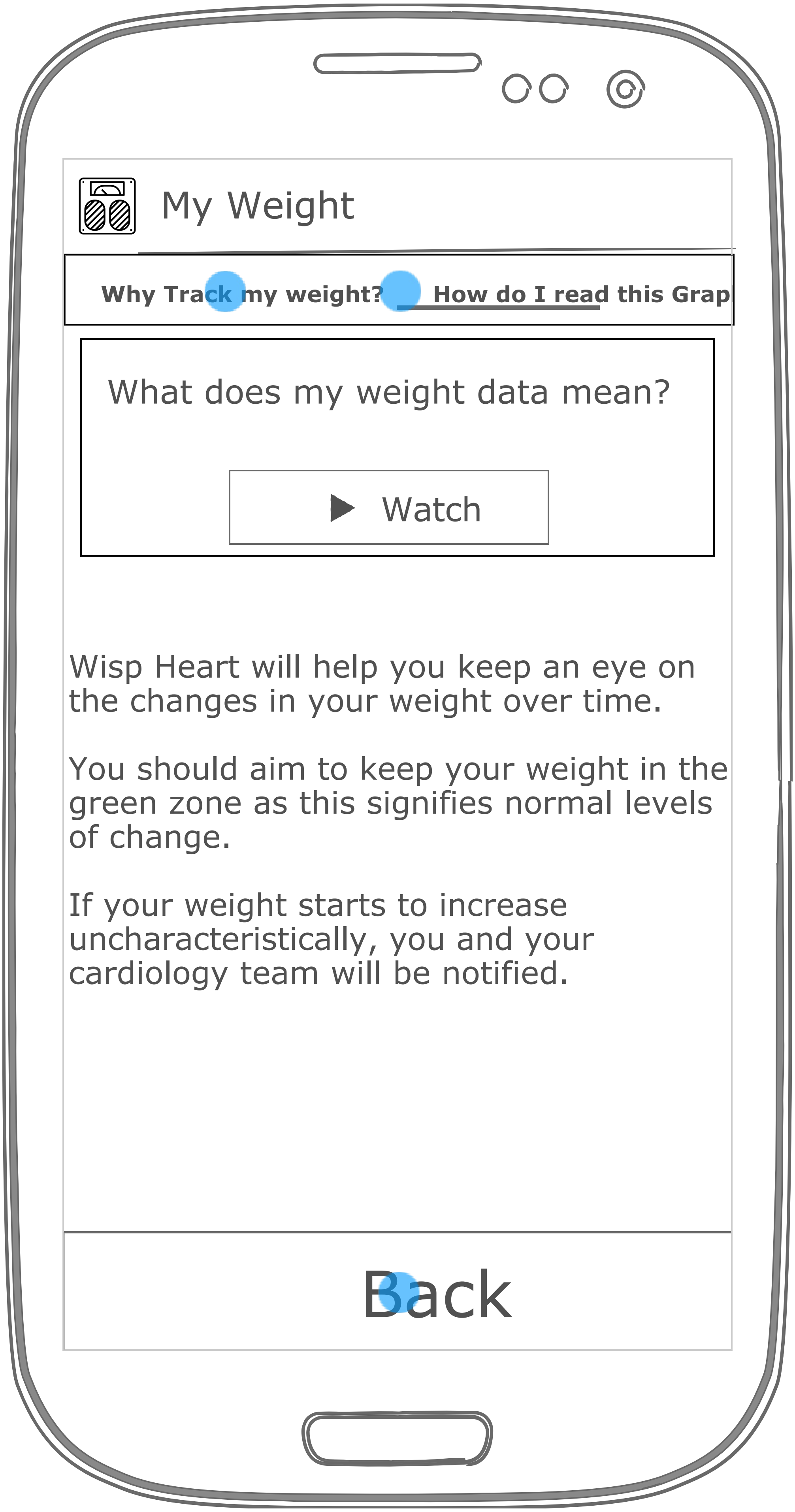

Sleep Graph Education

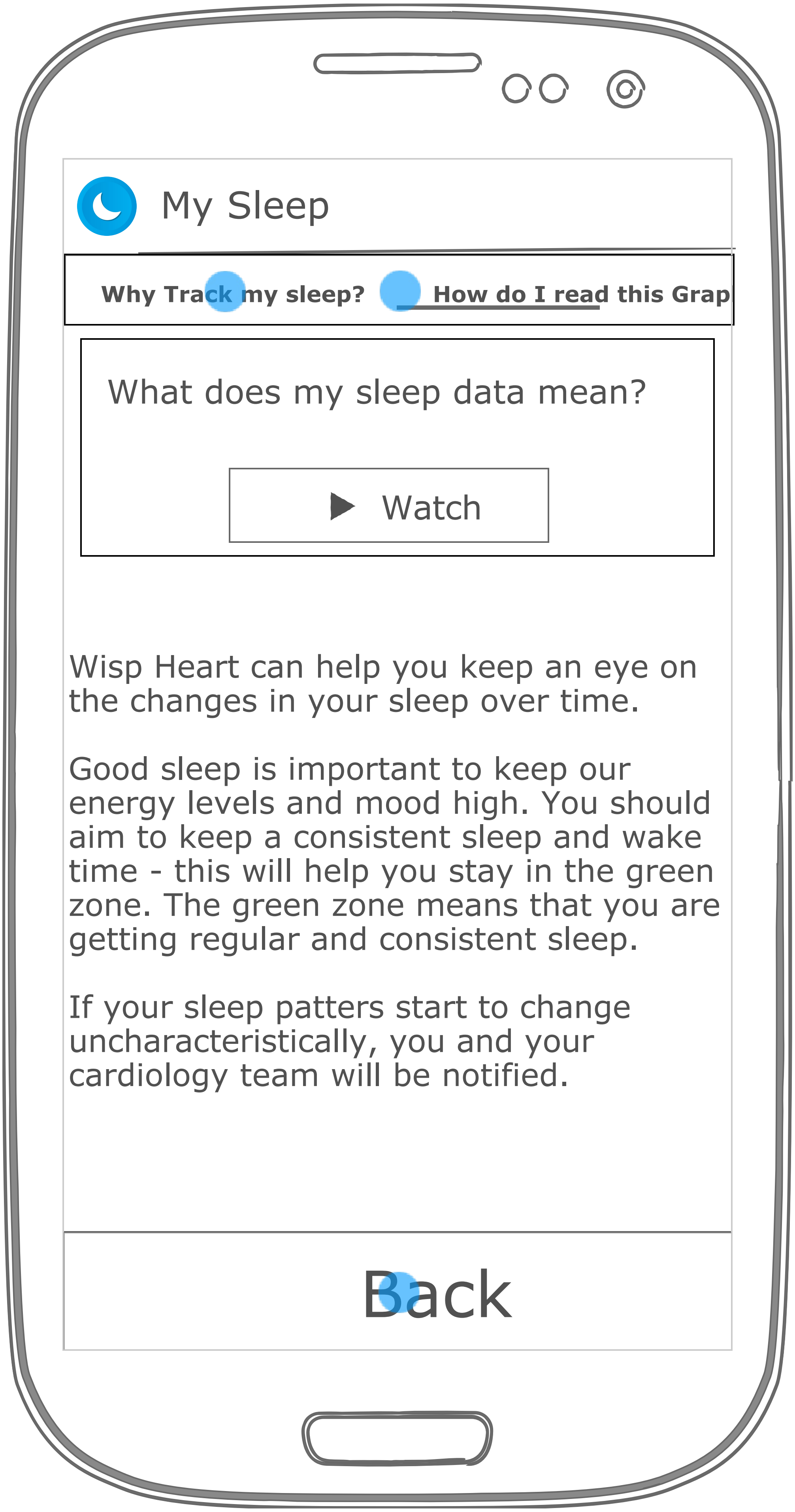

Sleep why measure

My Sleep

Why track my sleep? **How do I read this Graph?**

Generic Sleep image

Maintaining a consistent sleep pattern is an important aspect of your self-care.

While it is completely normal for your sleep patterns to change a little from night to night, a consistent change from your usual pattern can indicate that your condition is changing.

For example, an increase in your sleep time over a week may indicate that you are suffering from fatigue, while a decrease in sleep time may be caused by increases in symptoms such as breathlessness.

Back

HR Graph Education

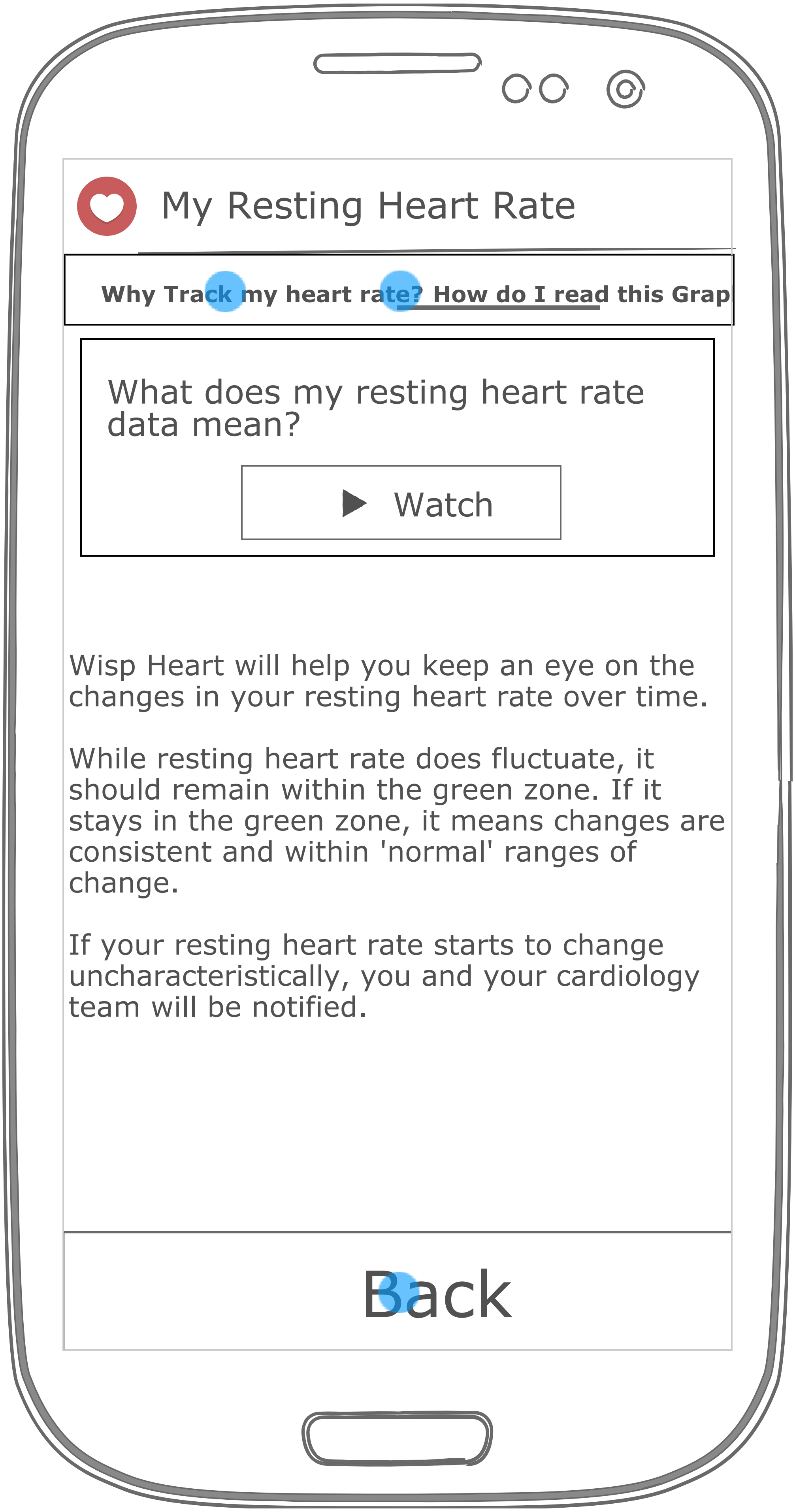

My Resting Heart Rate

Why track my heart rate? **How do I read this Graph?**

Generic Heart Rate Image

Resting heart rate is your heart rate while you are at rest. It is calculated automatically by your Fitbit.

Resting heart rate is an important indicator of your condition, providing a snap-shot into the functioning of your heart.

Large changes in resting heart rate can be caused by changes in your condition, medications, exercise levels, sleep and alcohol consumption.

Back

My exercise

Why Track my exercise? **How do I read this Grap**

What does my exercise data mean?

▶ Watch

Wisp Heart will help you keep an eye on the changes in your activity over time. This should be used alongside the guidance provided by your cardiology team.

You should aim to keep consistent exercise levels - this will help you stay in the green zone. Staying in the green zone means you are pacing your changes appropriately. Consistent, smaller changes to activity are the safest way to build your fitness levels.

If your activity patters start to change uncharacteristically, you and your cardiology team will be notified.

Back

Activity why measure

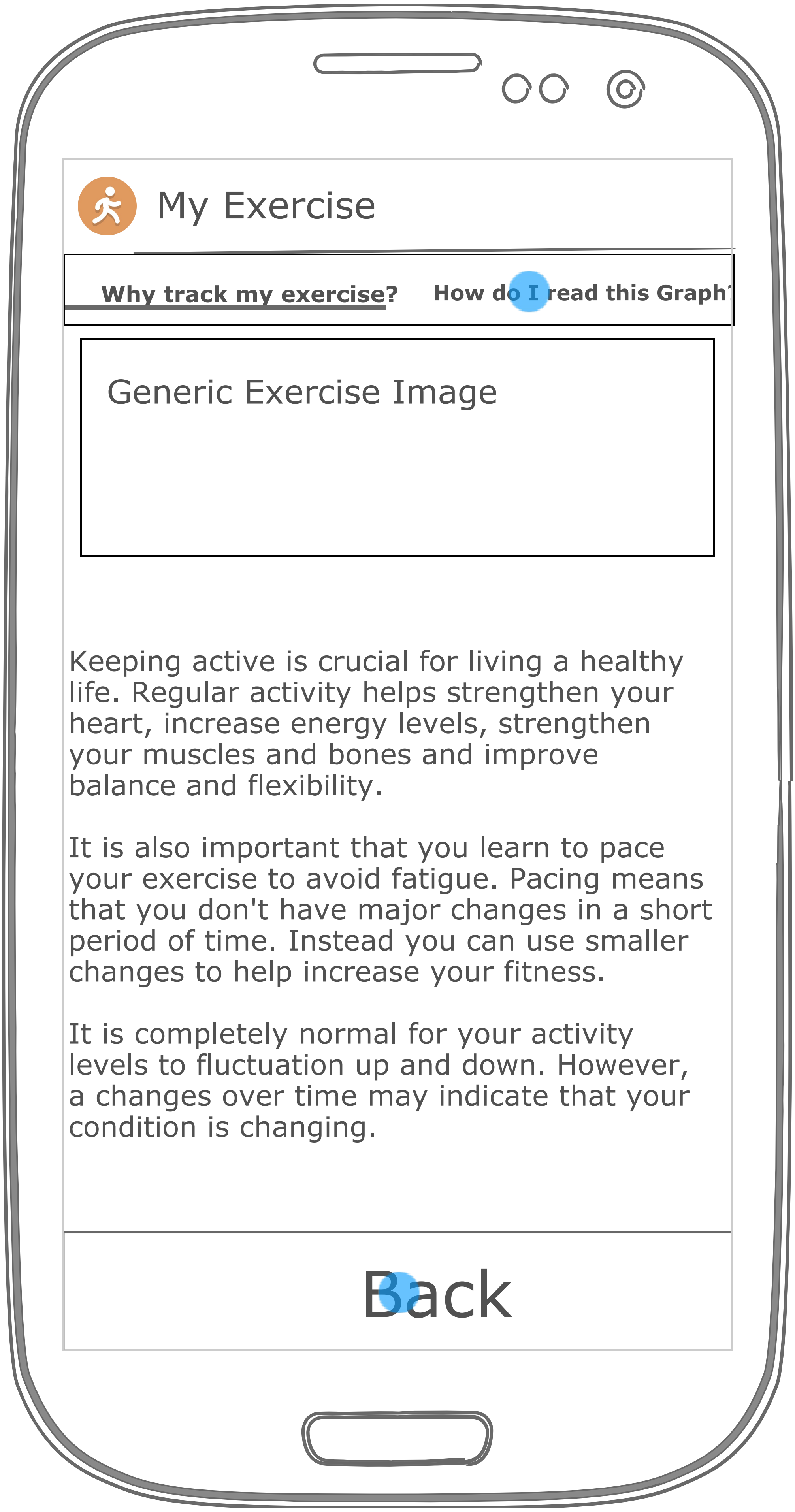

Self Alert

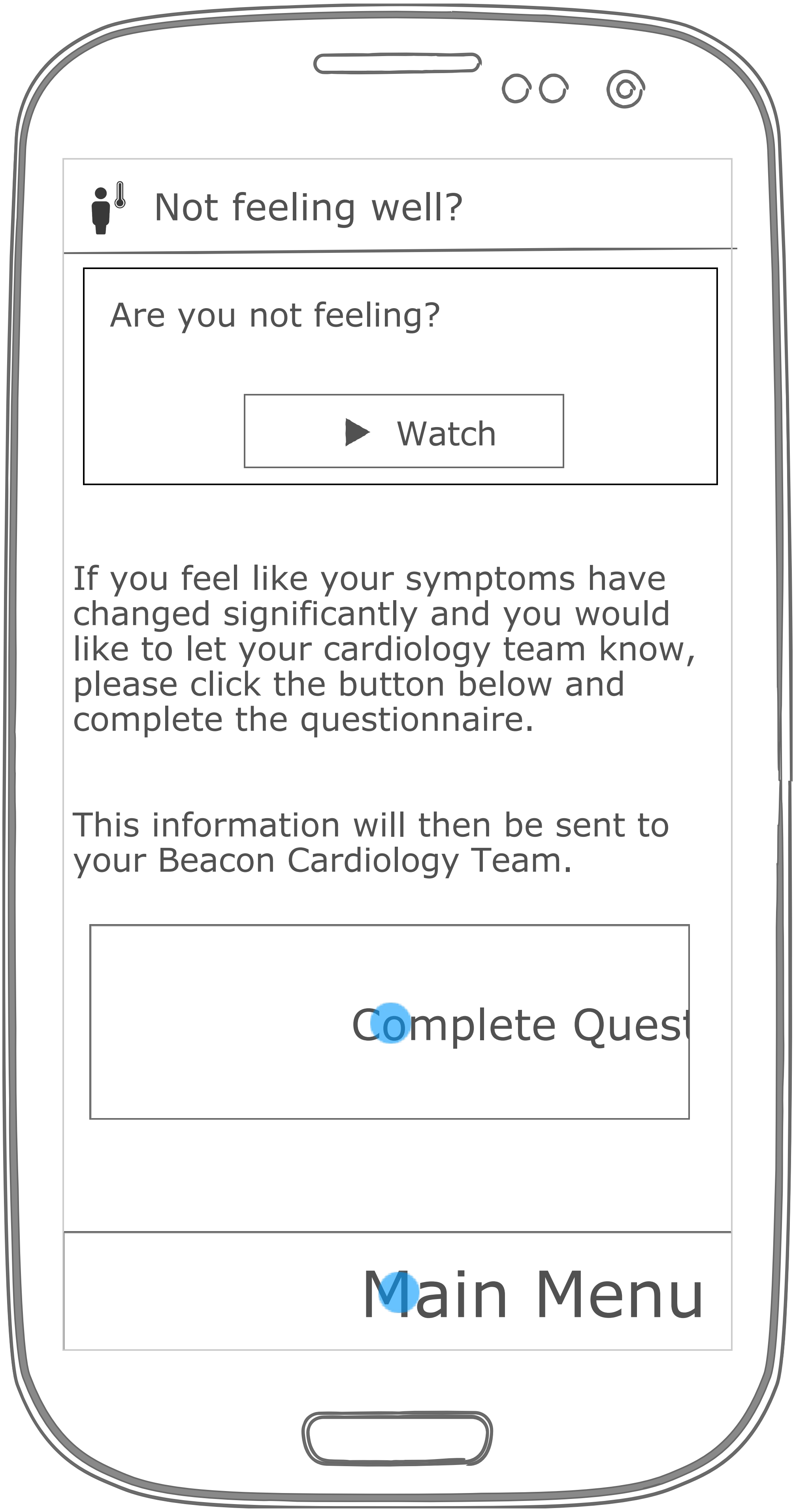
Not feeling well?
Are you not feeling?
Watch
If you feel like your symptoms have changed significantly and you would like to let your cardiology team know, please click the button below and complete the questionnaire.
This information will then be sent to your Beacon Cardiology Team.
Complete Quest
Main Menu

Alert Finish

Symptom Questionnaire

Thank you for completing the questionnaire.

An alert has now been sent to the Beacon Cardiology Team.

You will be contacted within the next 24-48 hours.

If you need urgent medical help, call 999 immediately.

If you would like to speak to Beacon Cardiology, please contact +3531 293 6694

Main Menu

Alert q1

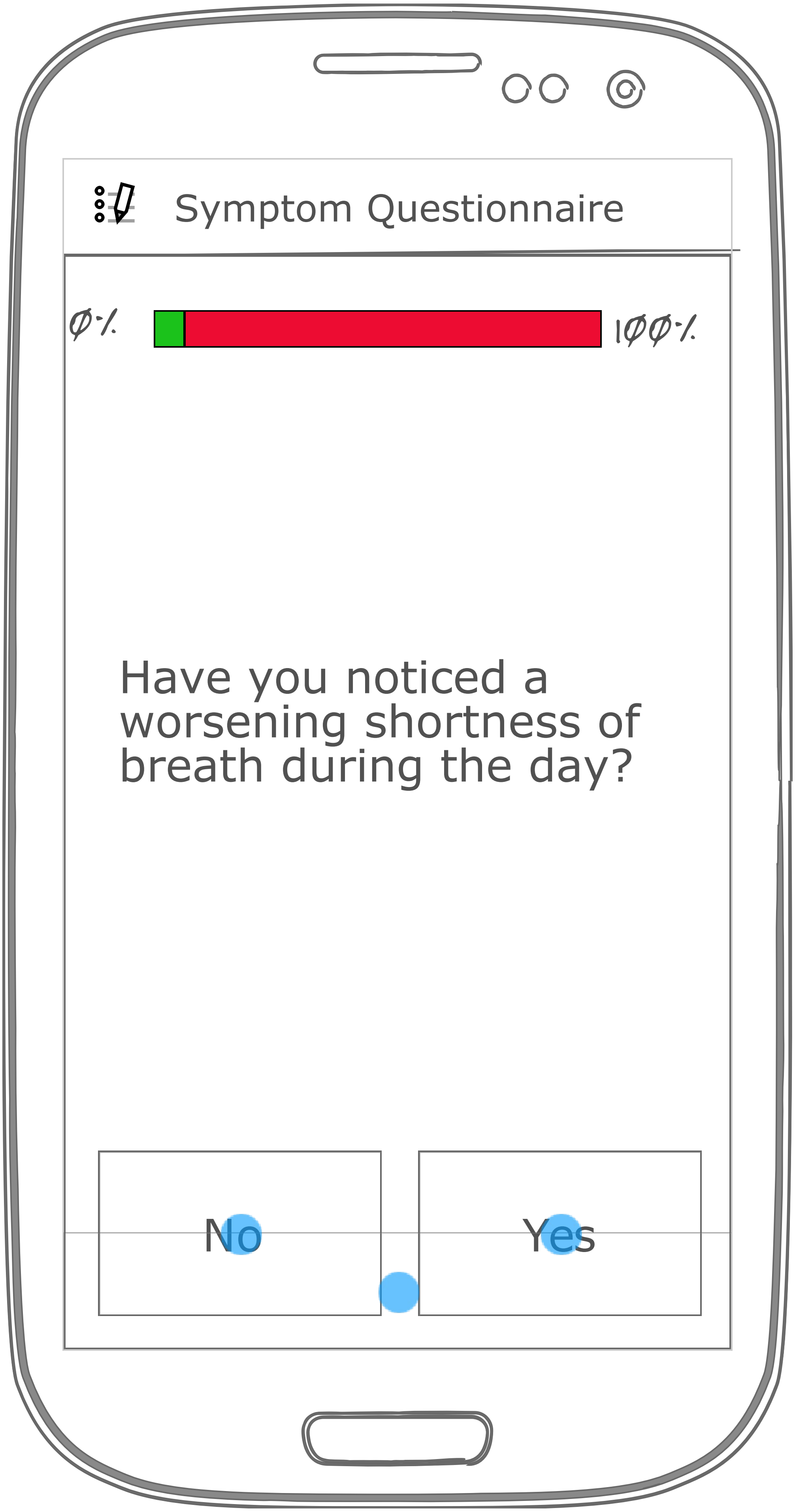

Symptom Questionnaire
0%
100%
Have you noticed a worsening shortness of breath during the day?
No
Yes

Alert q2

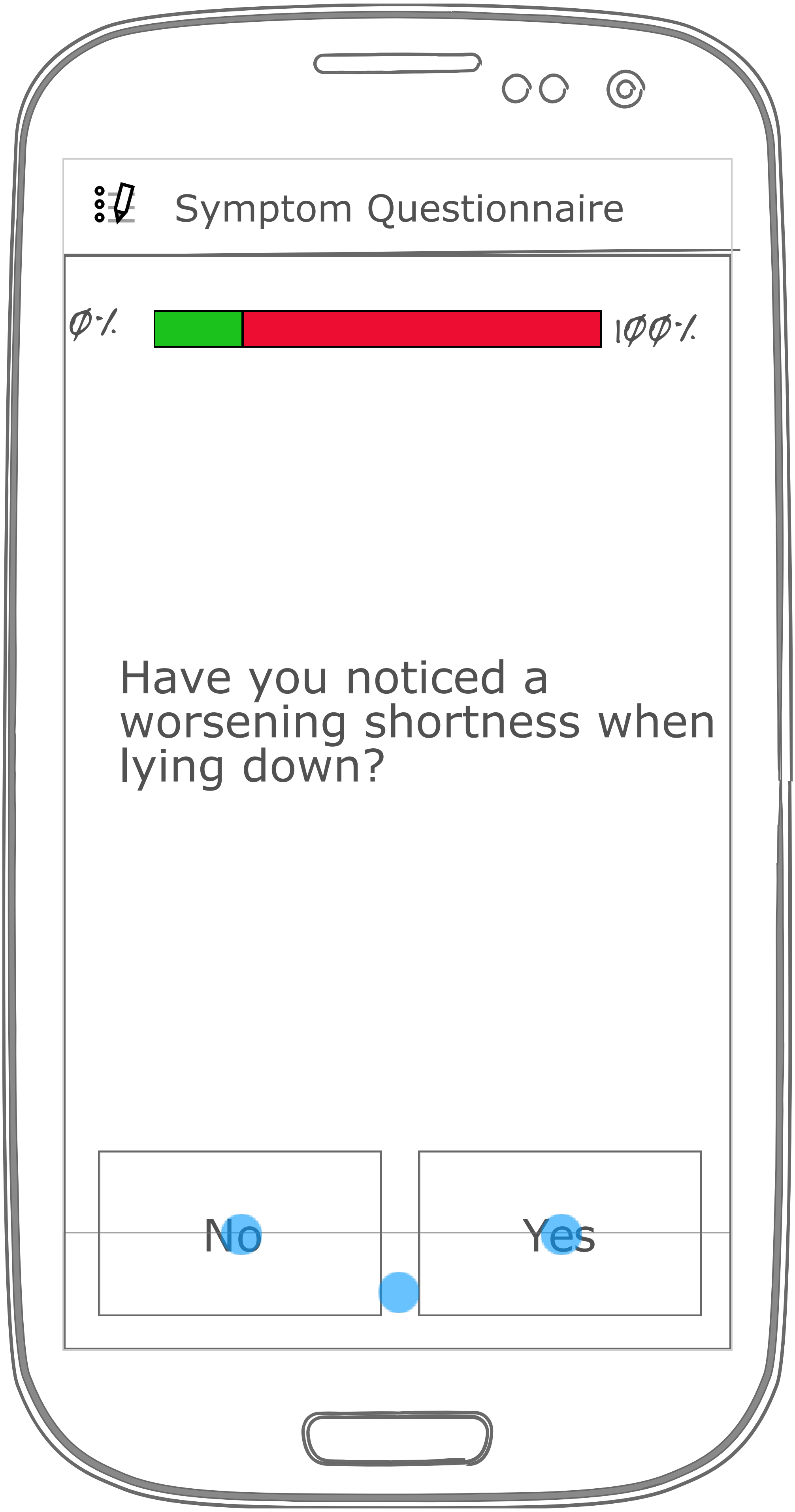
Symptom Questionnaire
0%
100%
Have you noticed a worsening shortness when lying down?
No
Yes

alert q3

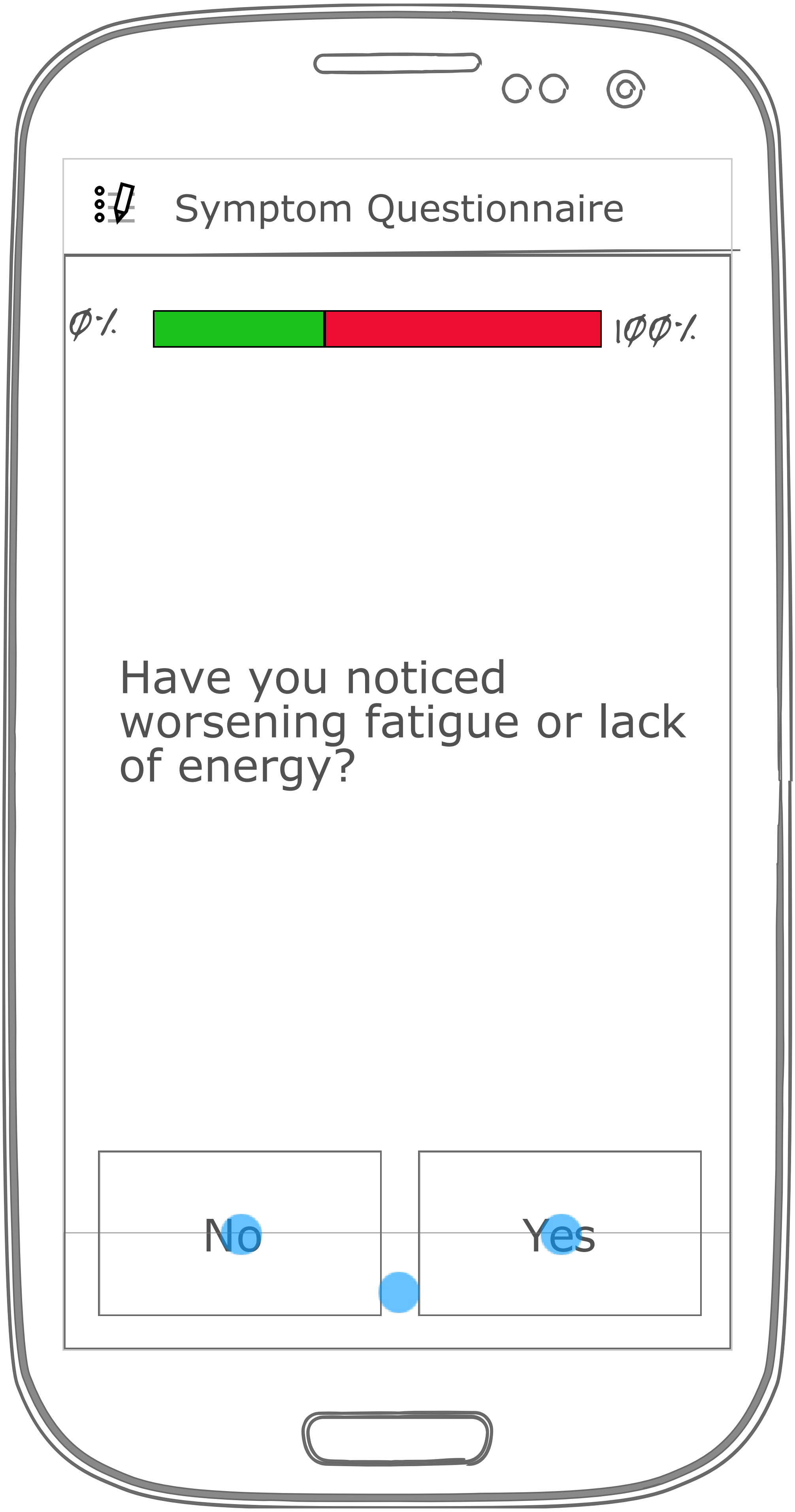
Symptom Questionnaire
0%
100%
Have you noticed worsening fatigue or lack of energy?
No
Yes

alert q4

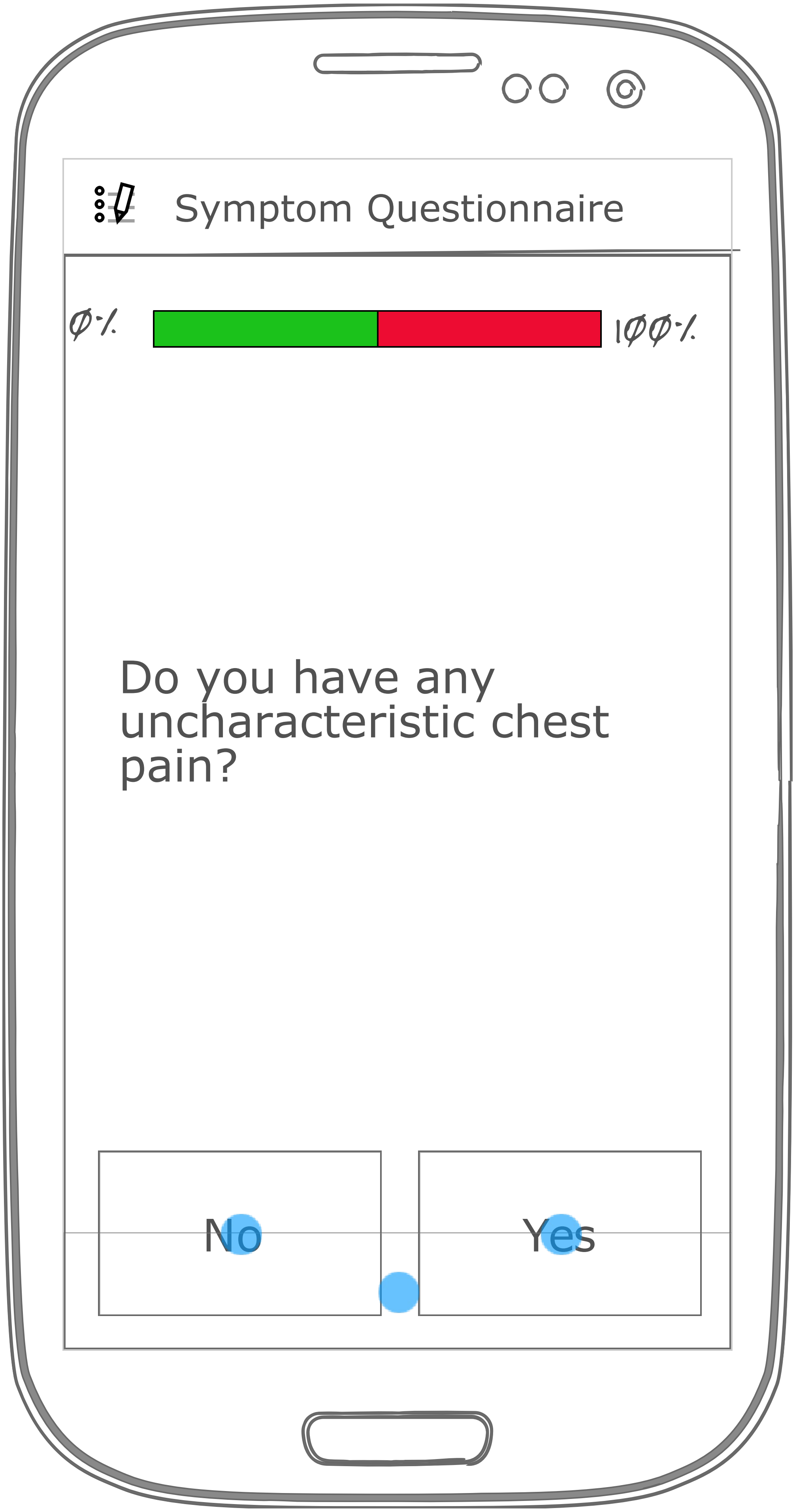

Symptom Questionnaire
0%
100%
Do you have any uncharacteristic chest pain?
No
Yes

alert q5

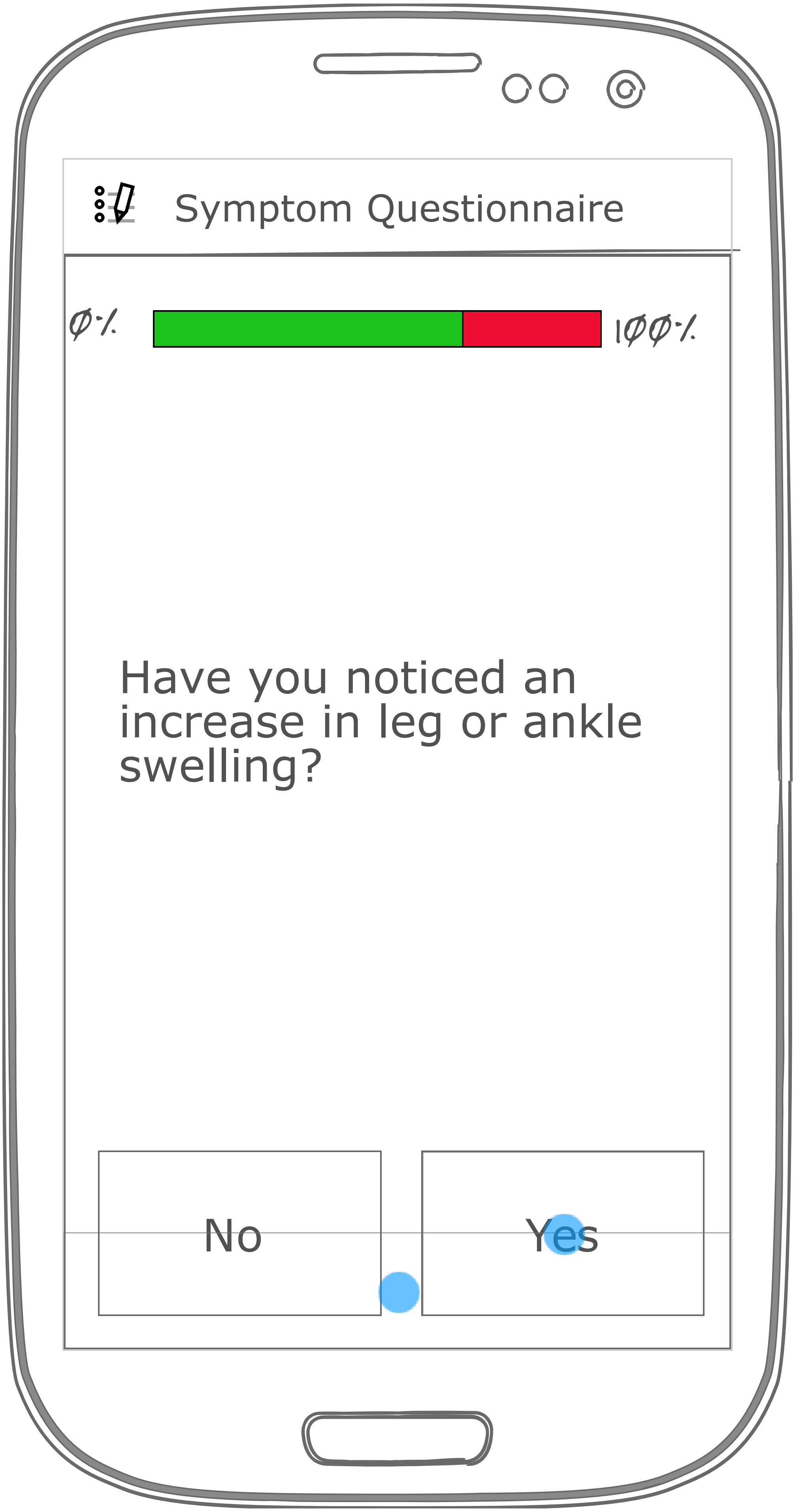
Symptom Questionnaire
0%
100%
Have you noticed an increase in leg or ankle swelling?
No
Yes

alert q6

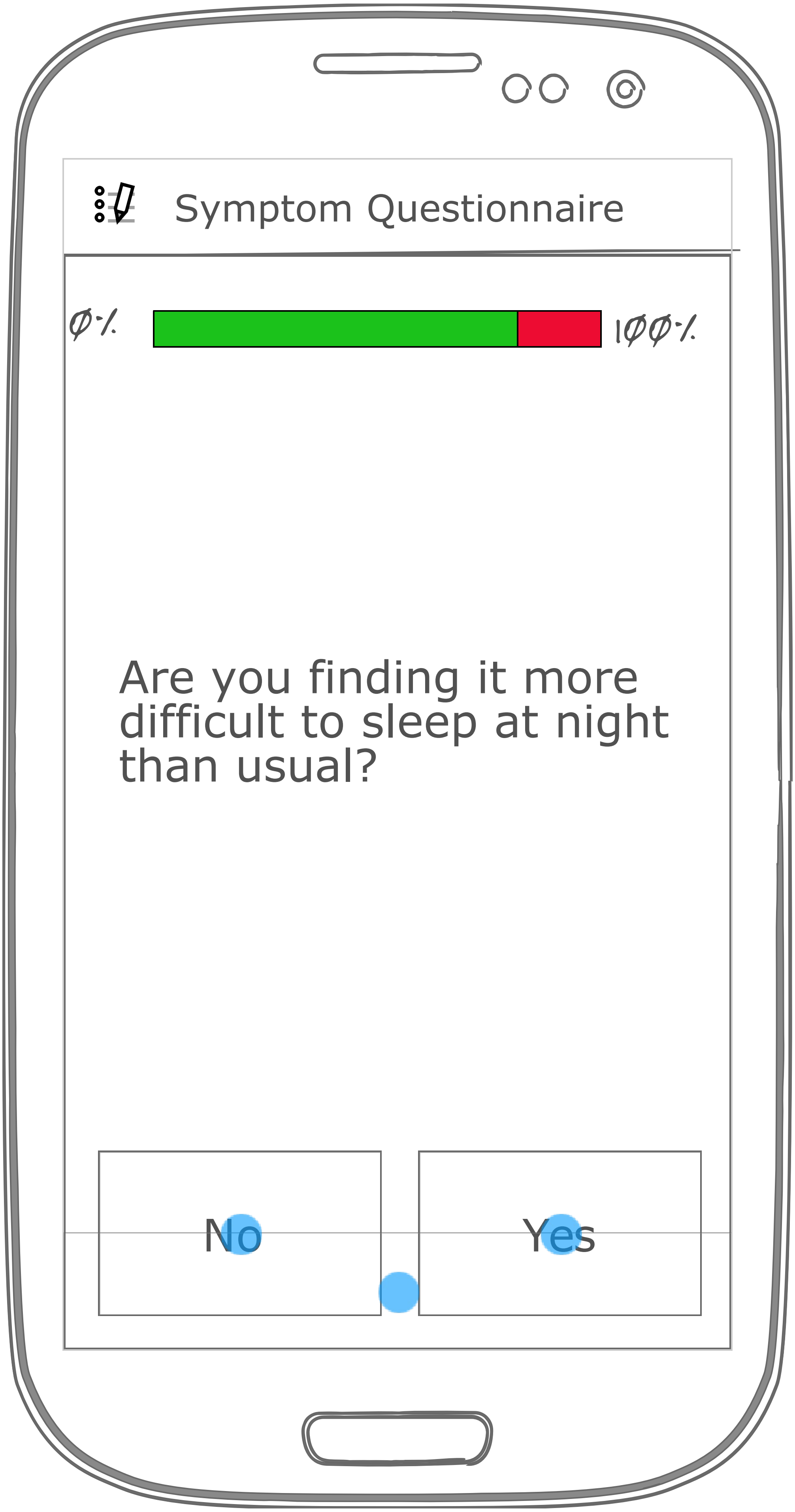
Symptom Questionnaire
0%
100%
Are you finding it more difficult to sleep at night than usual?
No
Yes

alert q7

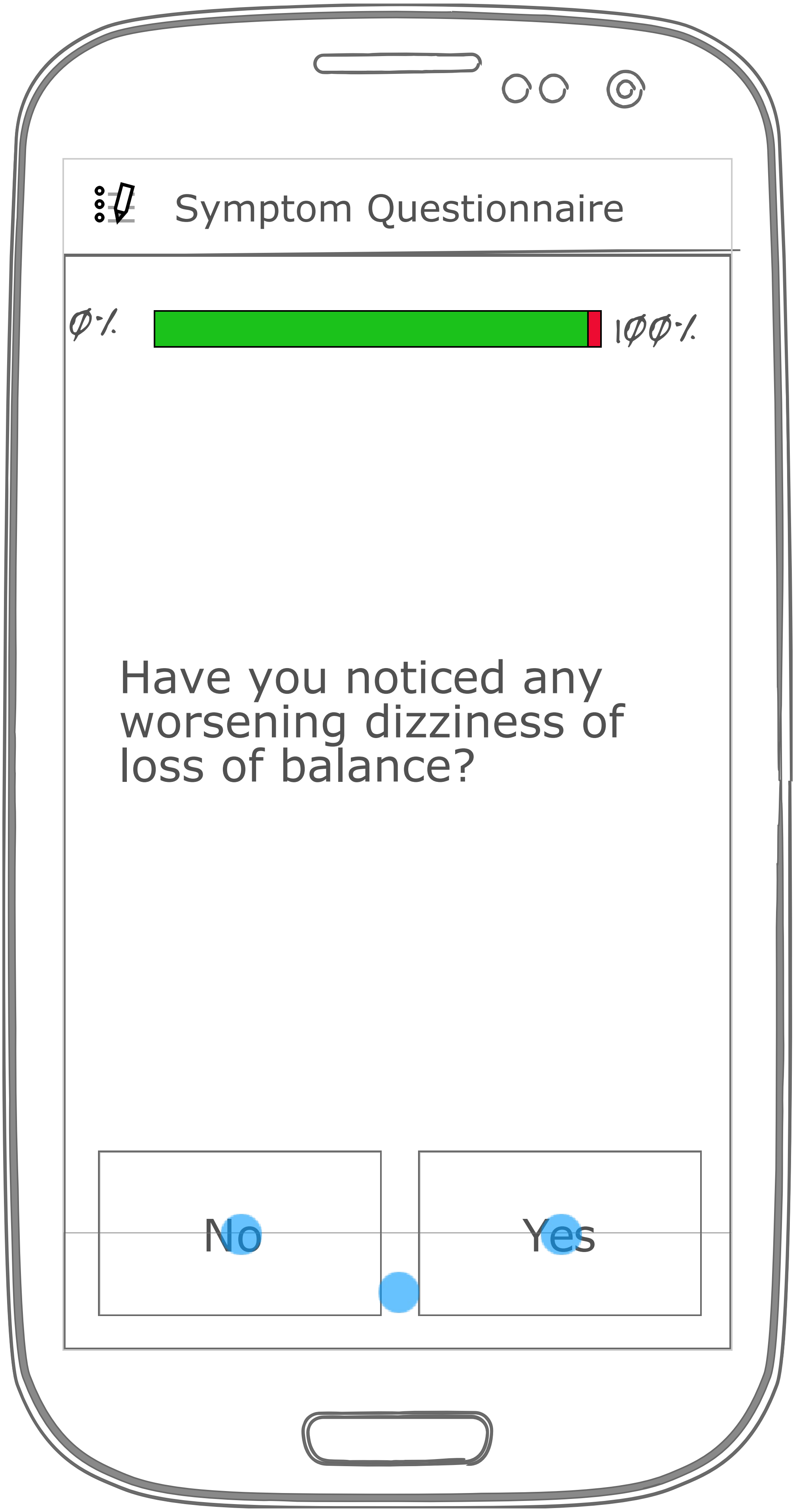
Symptom Questionnaire
0%
100%
Have you noticed any worsening dizziness of loss of balance?
No
Yes

Triggered Alert Weight Example

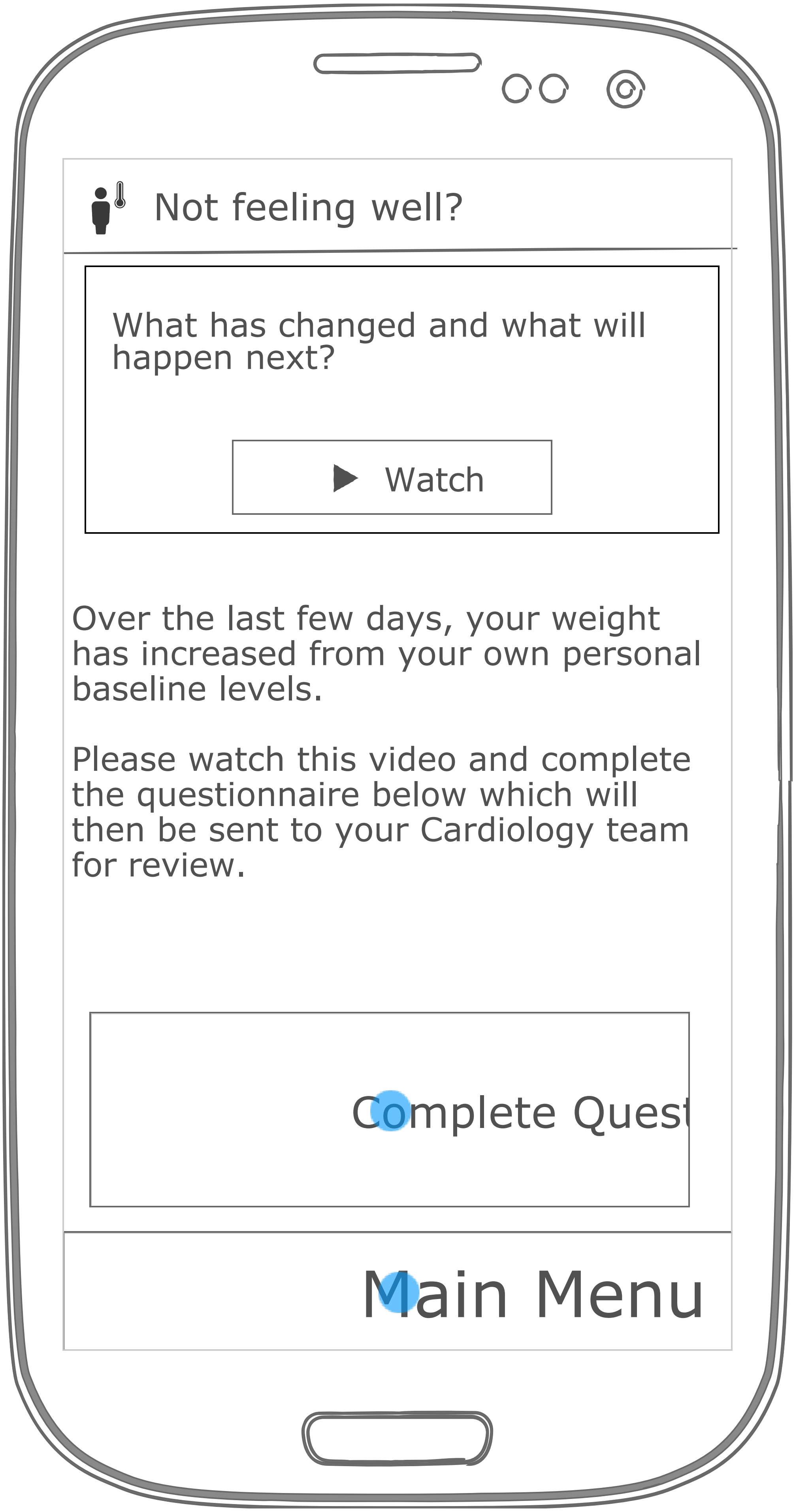

Triggered Alert q1

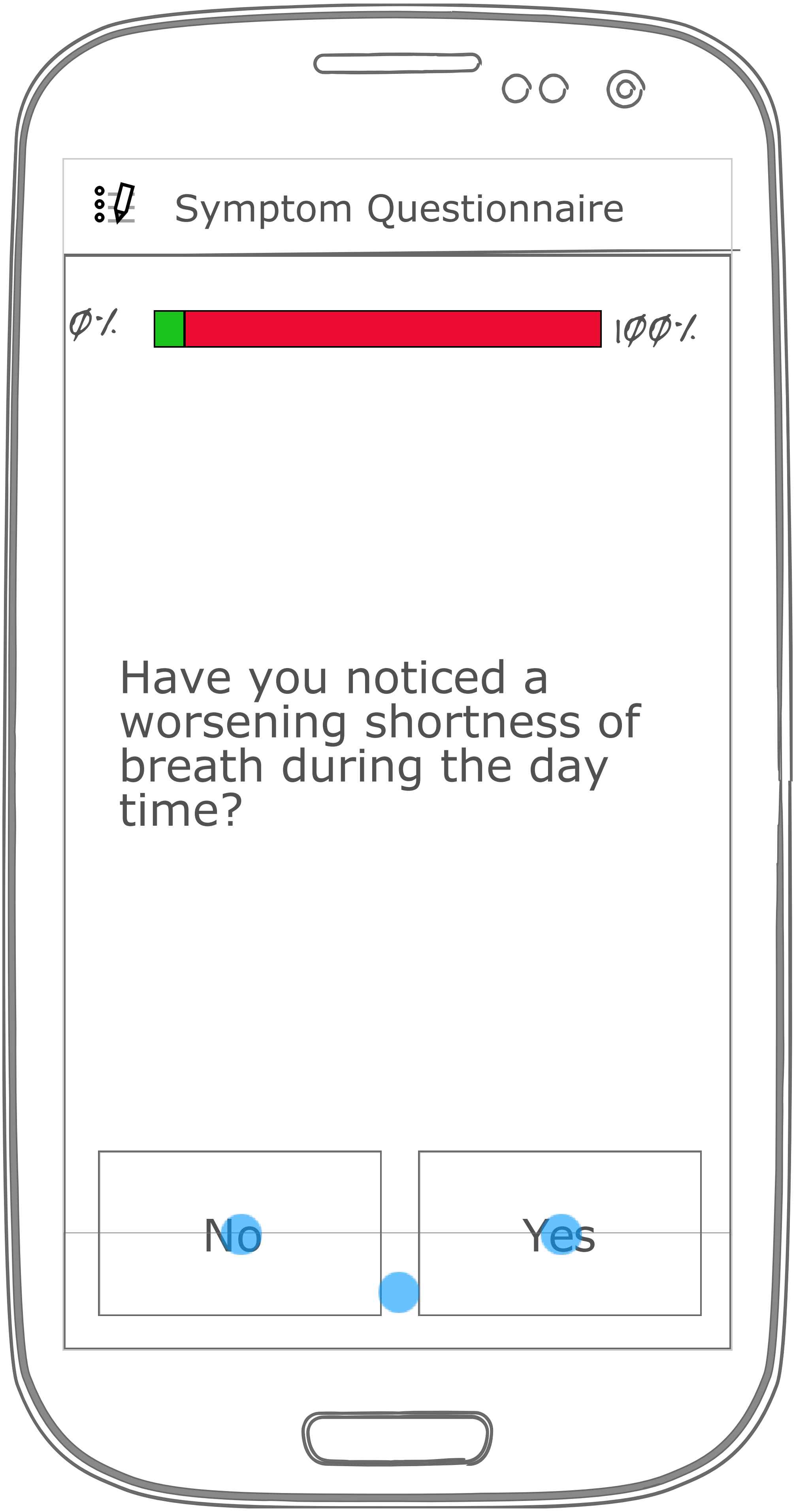

Triggered Alert q2

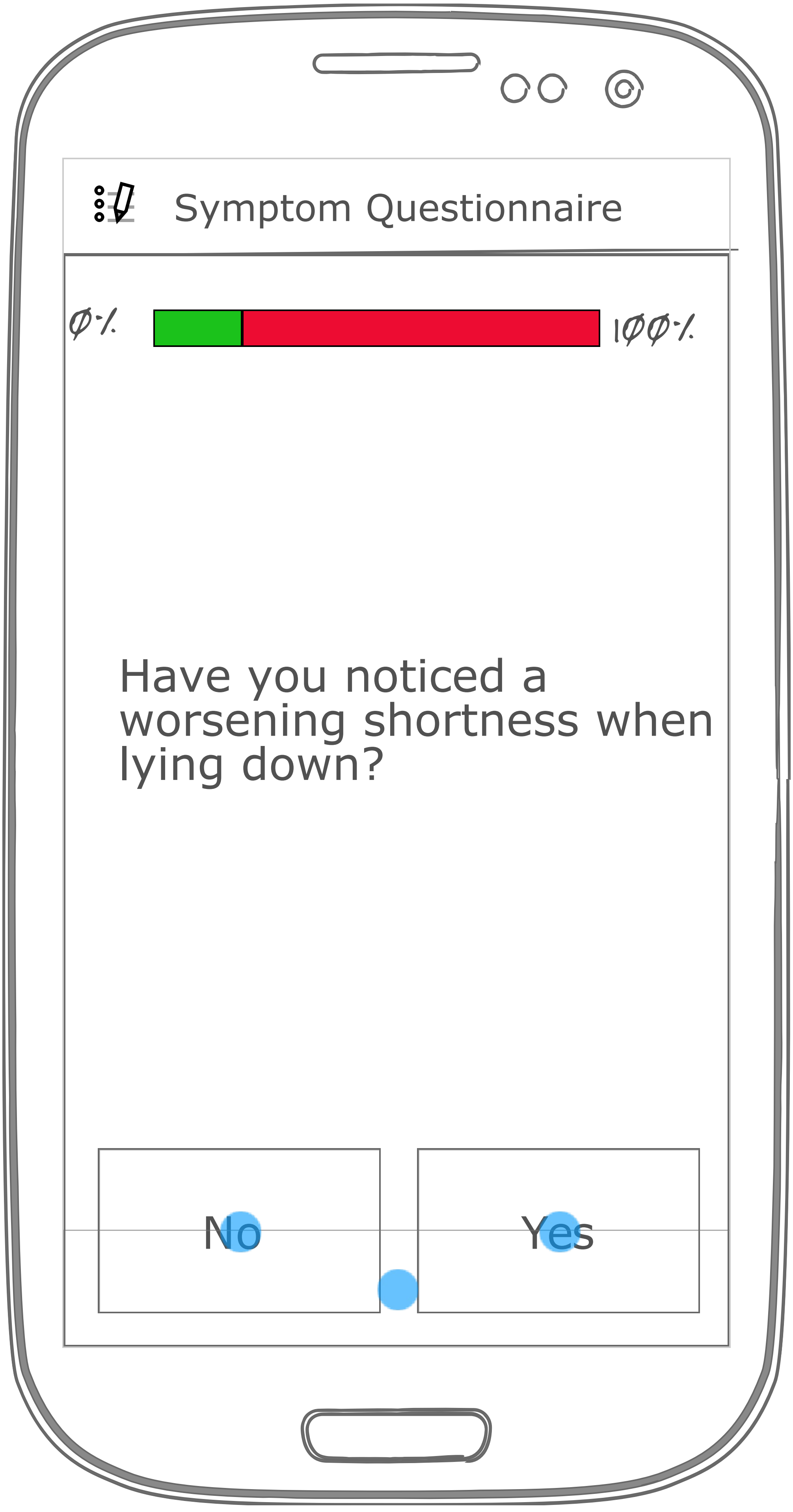

Triggered alert q3

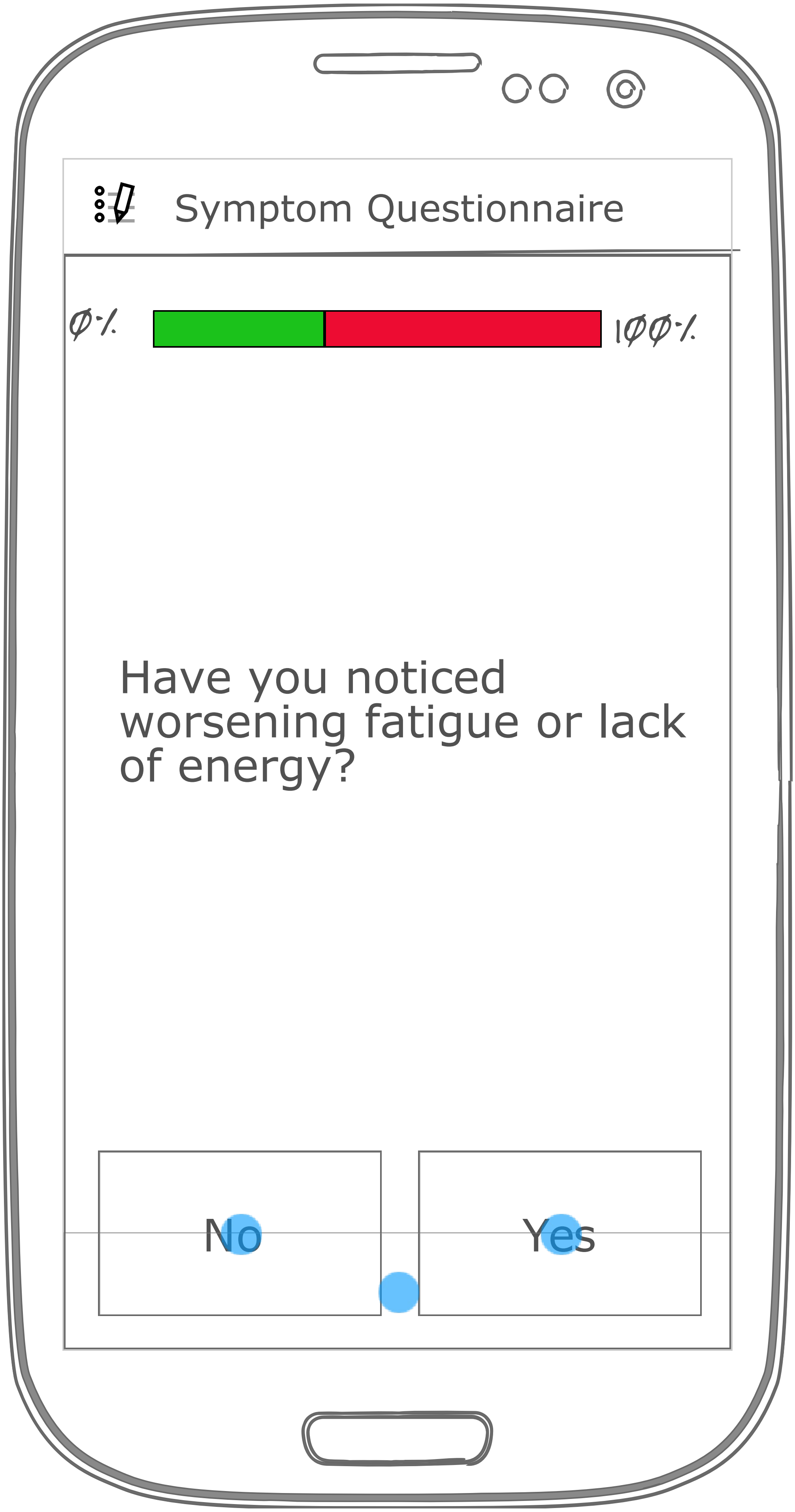

Triggered alert q4

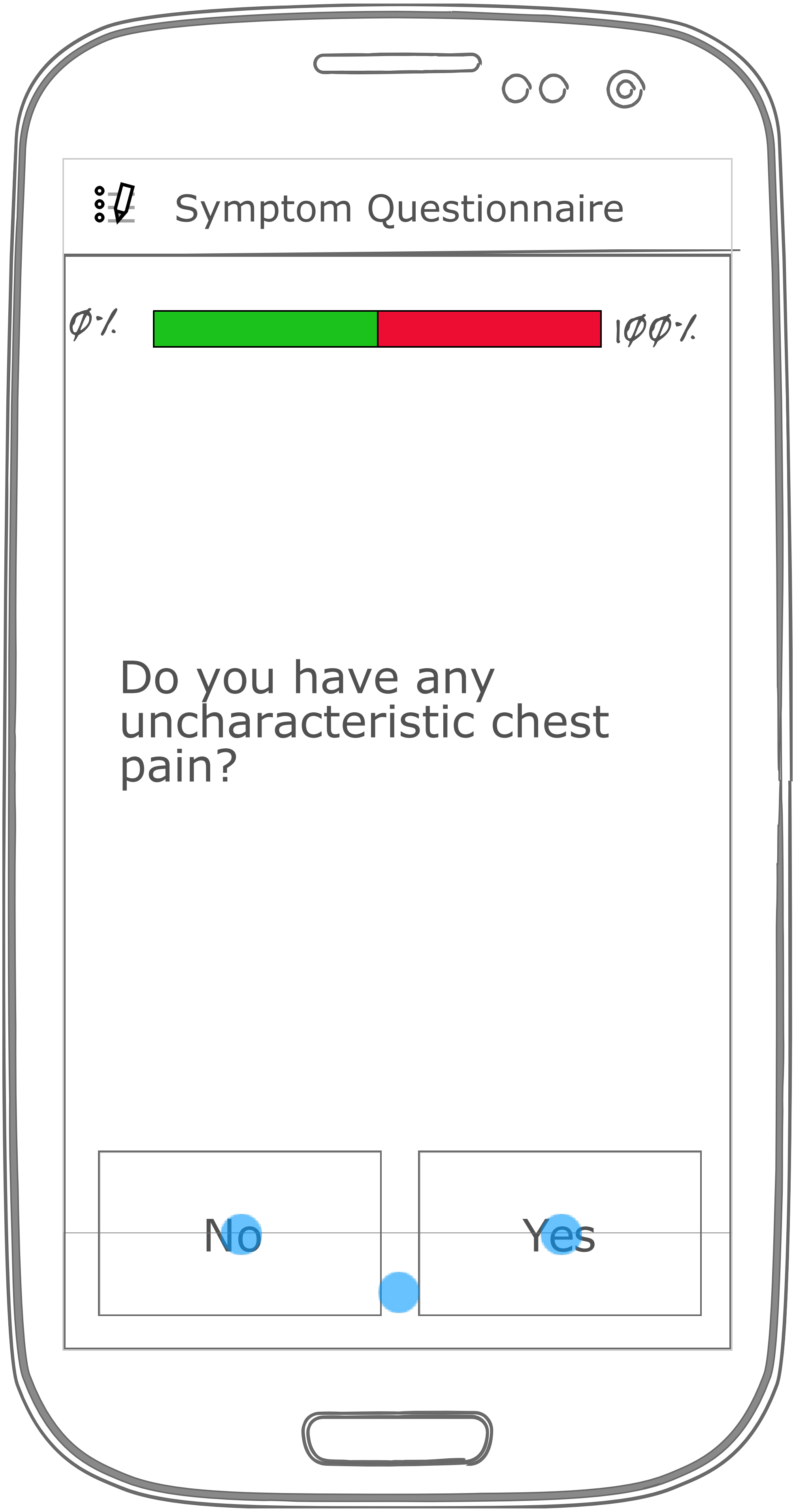

Triggered alert q5

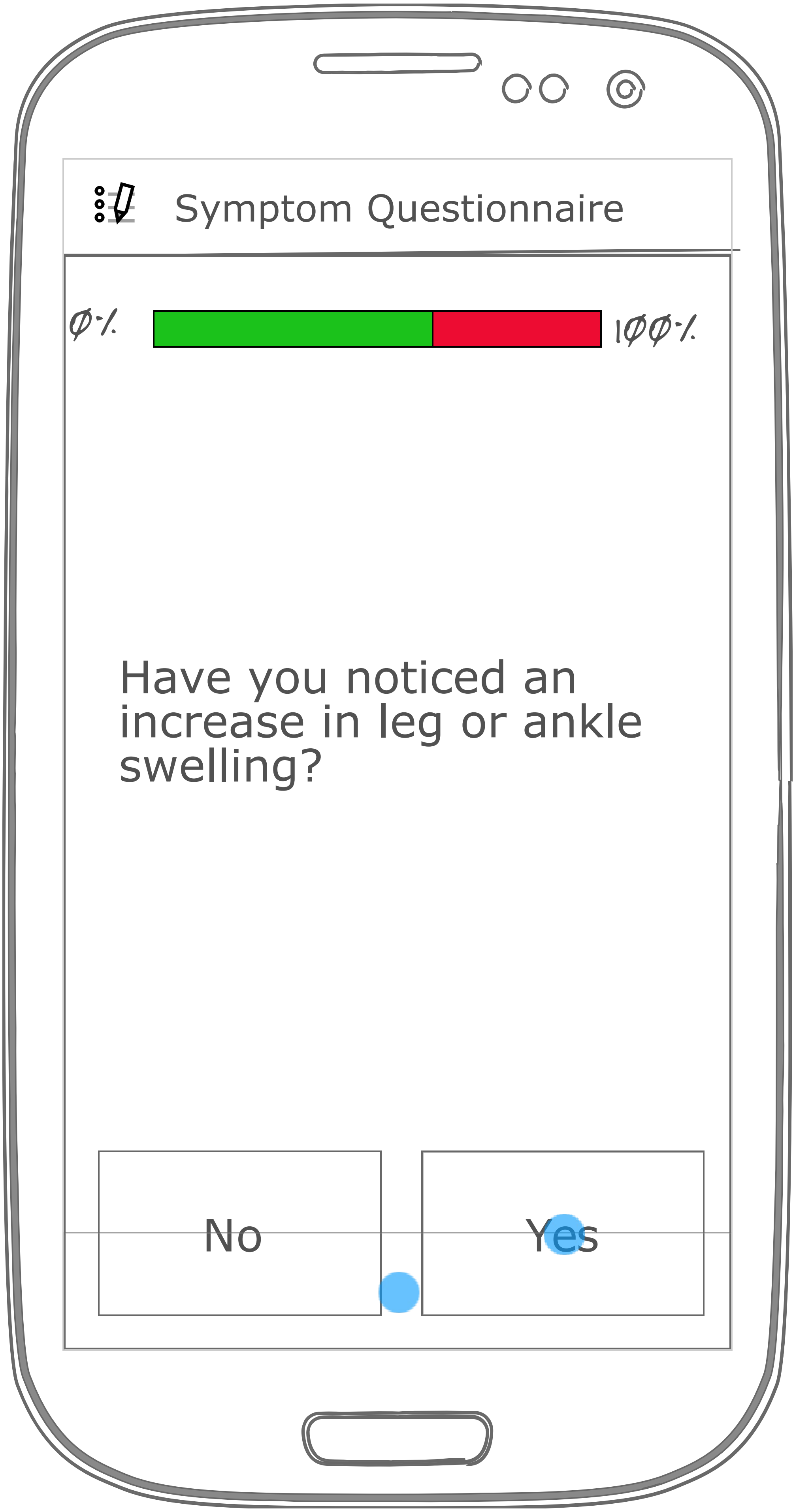

Triggered alert q6

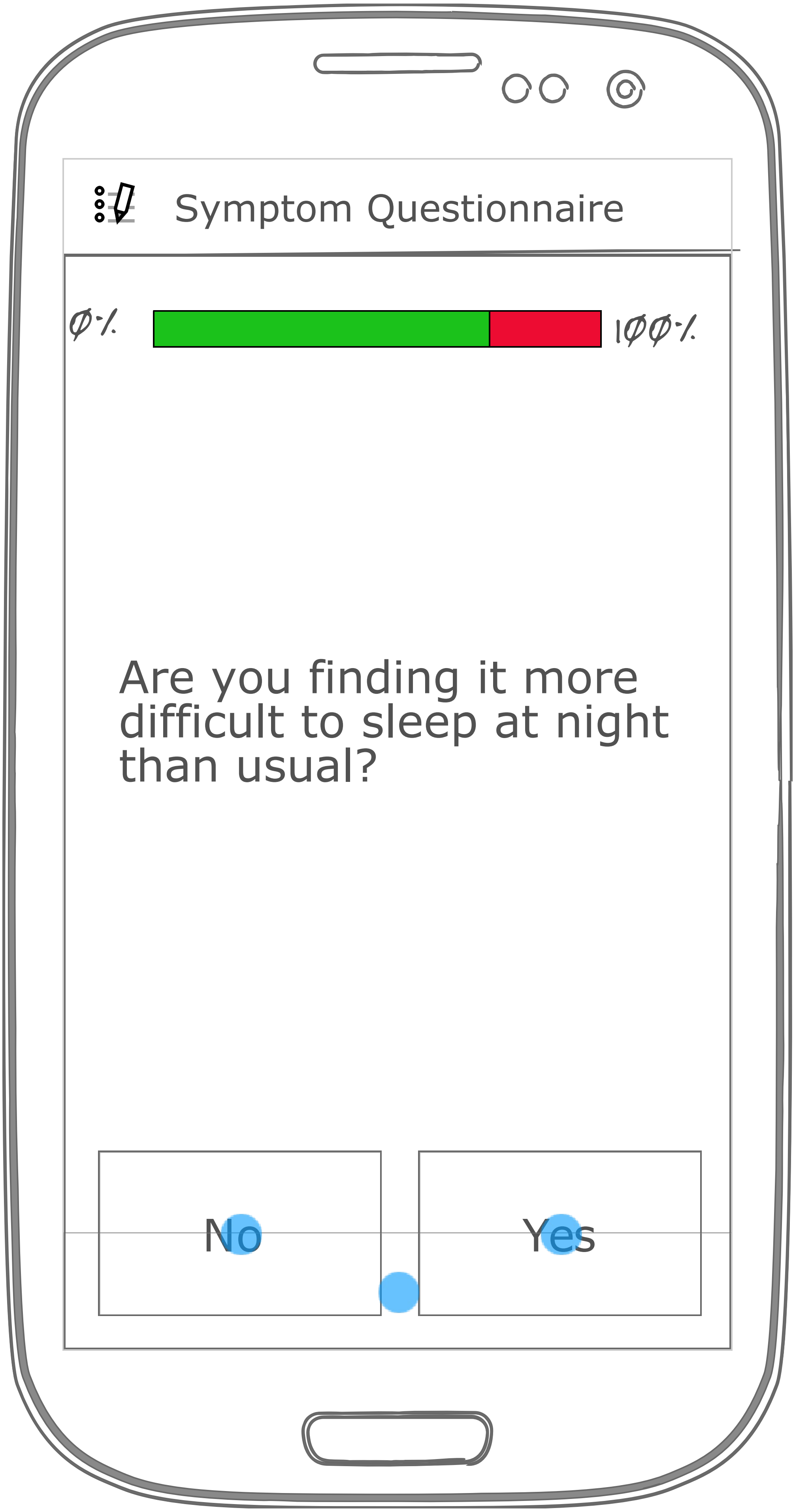

Triggered alert q7

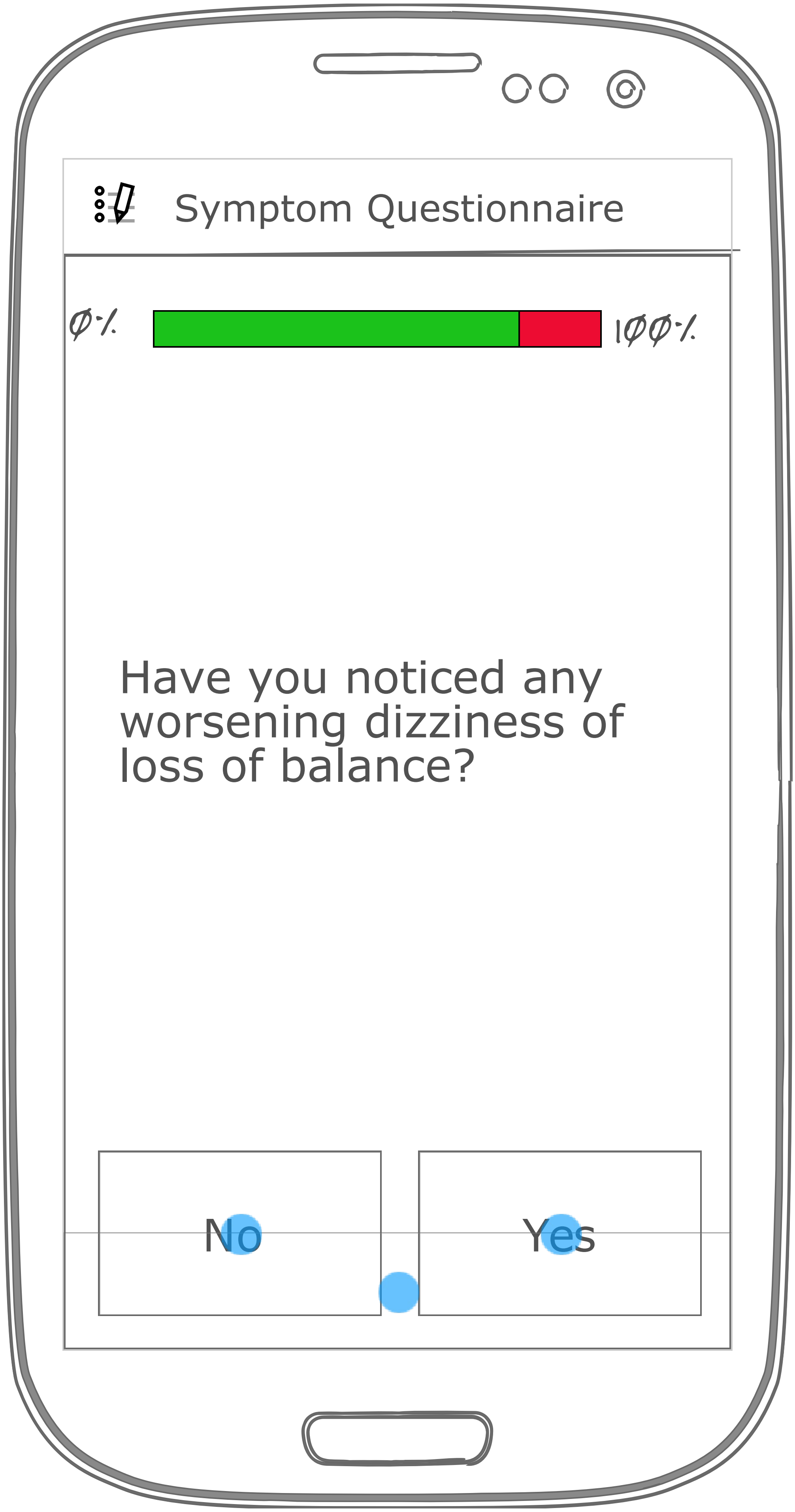

Triggered alert q8

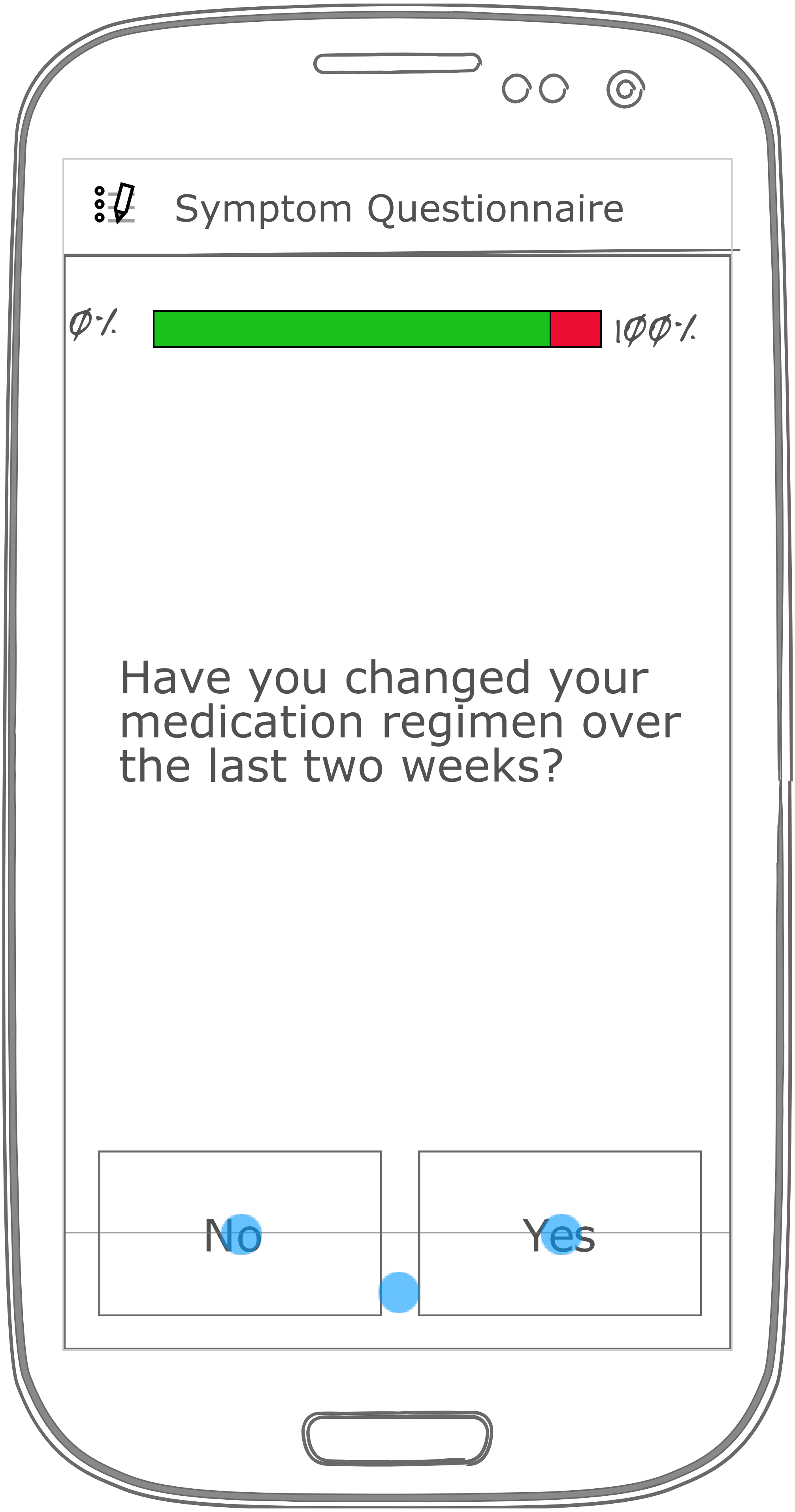

Triggered alert q9

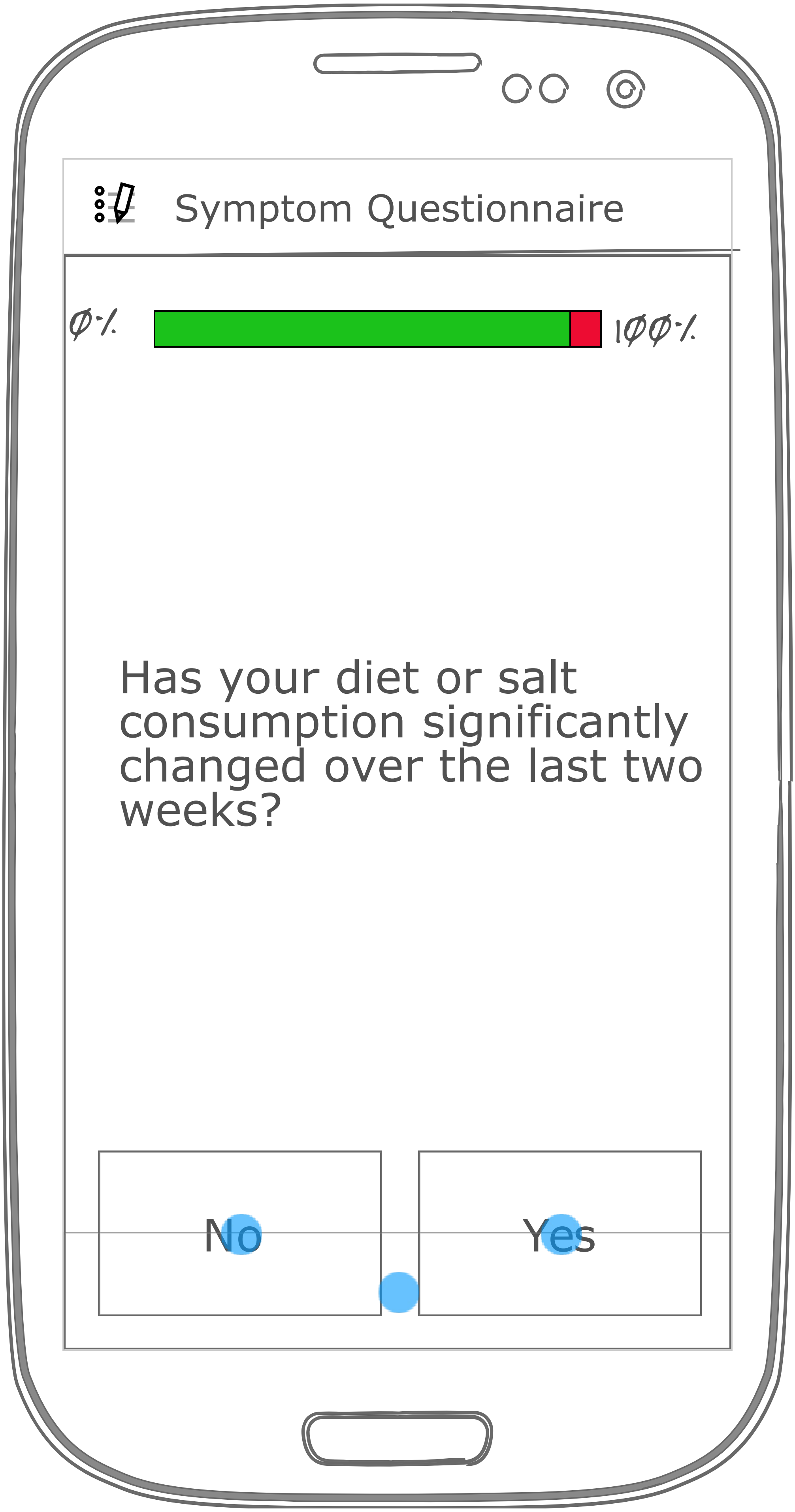

Triggered alert q10

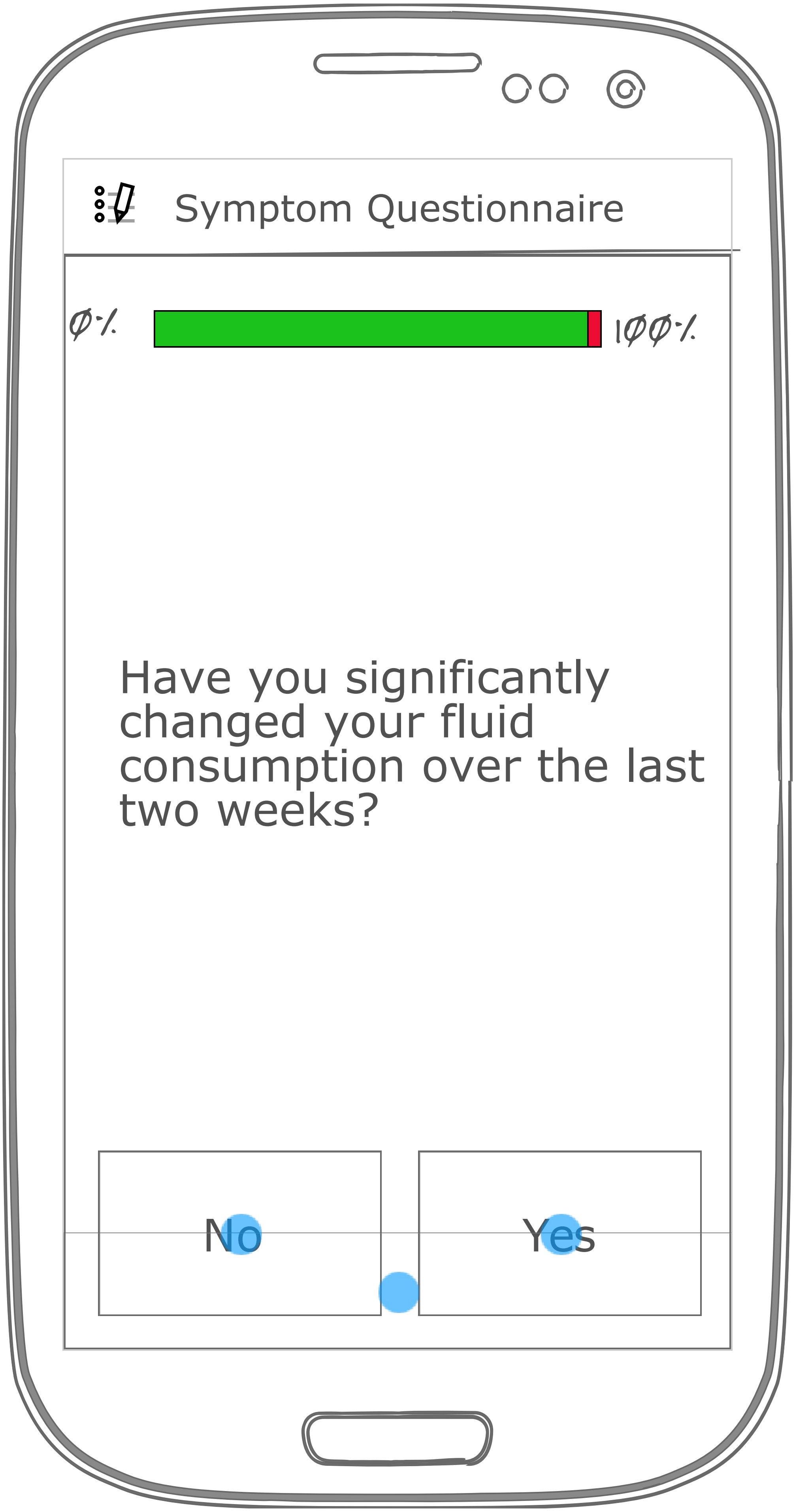

Triggered Alert Finish

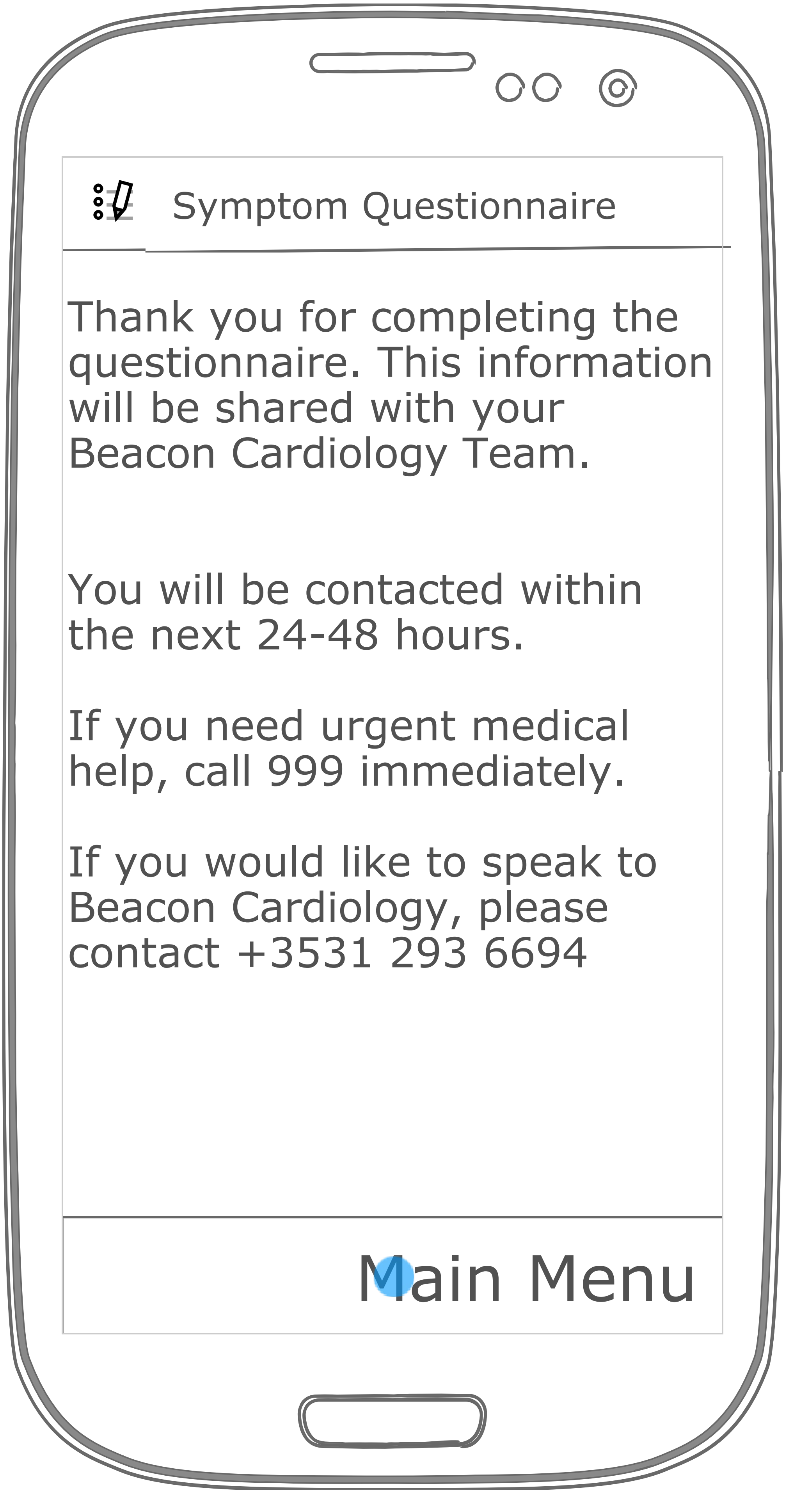

Supplement: Multimedia Appendix 4 [file formative_v6i5e34257_app4.pdf]
