# Supplementary material for: Human-Centered Design of a Digital Health Tool to Promote Effective Self-care in Patients With Heart Failure: Mixed Methods Study
Source: JMIR Form Res. 2022 May 10;6(5):e34257. doi: 10.2196/34257 (PMC9131139; doi:10.2196/34257)
Supplement: Multimedia Appendix 5 [file formative_v6i5e34257_app5.docx]

# Supplemental Appendix 5

Table 1: Mapping of the behaviour change techniques to the specific educational content of the system and system requirements

| **#** | **Purpose** | **Type of Content** | **BCT** | **Requirements** |
| --- | --- | --- | --- | --- |
| 1 | How to set-up the scales. | Video | 4.1 | Screen video (IOS & Android) & scale set-up |
|  |  | Text |  | Link to Fitbit setup |
| 2 | How to set-up the Fitbit | Video | 4.1 | Screen video (IOS & Android) & Fitbit set-up |
| 3 | How to sync the Fitbit | Video |  | Screen video (IOS & Android) |
| 4 | Navigating around the application | Video | 4.1 | Screen video |
|  |  |  |  |  |
|  | **General Condition Specific Education** |  |  |  |
| 5 | The importance of self-care in your heart conditions | Video | 2.3, 5.1, 8.1,15.1 | Animated |
| 6 | How the app can help you manage your heart condition | Video |  | Animated & Screen recording |
|  |  |  |  |  |
|  | **General Variable Specific Education** |  |  |  |
| 7 | Why it is important to track your weight? | Video | 2.1, 2.2, 2.3, 2.6, 2.5, 2.7, 5.1, 5.3, 7.1, 8.1, 8.3, 9.1, 11.1, 15.1 | Consultant cardiologist speaking to camera & screen recording |
| 8 | Why it is important to track your sleep | Video |  | Consultant cardiologist speaking to camera & screen recording |
| 9 | Why it is important to track your resting Heart Rate | Video |  | Consultant cardiologist speaking to camera & screen recording |
| 10 | Why it is important to track your physical activity | Video |  | Consultant cardiologist speaking to camera & screen recording |
| 11 | How to interpret the weight data? | Video |  | Screen Recording |
|  |  | Text |  | Few lines on interpretation |
| 12 | How to interpret the sleep data? | Video |  | Screen Recording |
|  |  | Text |  | Few lines on interpretation |
| 13 | How to interpret the heart rate data? | Video |  | Screen Recording |
|  |  | Text |  | Few lines on interpretation |
| 14 | How to interpret the physical activity data? | Video |  | Screen Recording |
|  |  | Text |  | Few lines on interpretation |
| 15 | How to add medications | Video |  | Screen video |
| 16 | How to add vitals | Video |  | Screen video |
| 17 | How to add appointments | video |  | Screen video |
|  | **Alert Specific Education** |  |  |  |
| 18 | Why you received an alert for weight and what will happen next. | Video | 2.1, 2.2, 2.3, 2.6, 2.5, 3.1, 5.1, 5.3, 7.1, 8.1, 8.3, 9.1, 15.1 | Consultant cardiologist speaking to camera |
| 19 | Why you received an alert for sleep and what will happen next. | Video |  | Consultant cardiologist speaking to camera |
| 20 | Why you received an alert for heart rate and what will happen next. | Video |  | Consultant cardiologist speaking to camera |
| 21 | Why you received an alert for physical activity and what will happen next. | Video |  | Consultant cardiologist speaking to camera |
|  |  |  |  |  |
