# Supplementary material for: Human-Centered Design of a Digital Health Tool to Promote Effective Self-care in Patients With Heart Failure: Mixed Methods Study
Source: JMIR Form Res. 2022 May 10;6(5):e34257. doi: 10.2196/34257 (PMC9131139; doi:10.2196/34257)
Supplement: Multimedia Appendix 6 [file formative_v6i5e34257_app6.docx]

# Supplemental appendix 6

The system was designed to consider four endpoints obtained from the Fitbit API: step count, sleep time, weight and resting heart rate. The system considered three sliding time-based windows; acute window, buffer window and the personal baseline window.

**Acute window:** the most recent five calendar days. A valid acute window consisted of three measurements, within the five day window.

**Buffer window:** the seven days preceding the acute window.

**Baseline Window:** A 30 day window preceding the buffer window. A valid baseline window consisted most recent valid measurements from within this window (max 20 days; min 7 days).


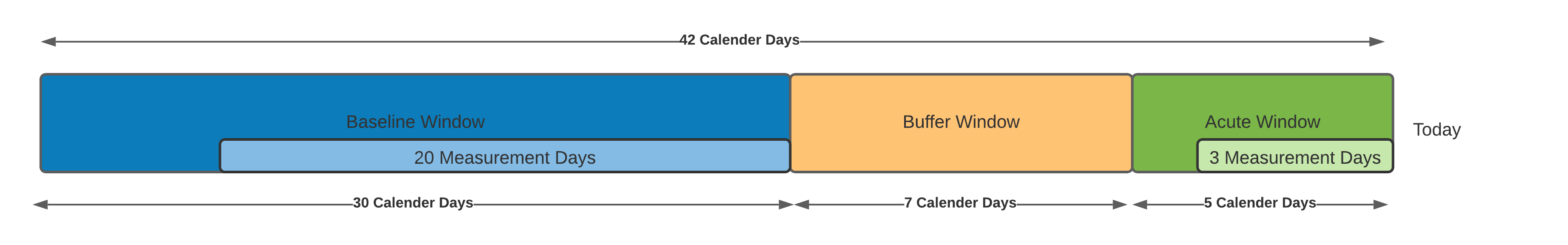
An individuals ‘personal baseline’ was considered as the range between the average of the valid measurements within the baseline window, plus (upper bound) or minus (lower bound) 2 x SD of the valid measurements within the baseline window. This was presented back to the user as a green zone, denoting the normal range for that particular endpoint. The system was also designed to automatically monitor for alterations in these endpoints from the individuals ‘personal baseline’. A change was considered as a >2SD alteration from the individuals baseline window, for three consecutive days within the acute window [1]. As detailed within the manuscript, for the purpose of the two week trial, only the visual feedback of the ‘personal baseline’ was used in the trial. Future evaluation of the long-term use of this system will incorporate the full automated monitoring and symptom questionnaire triggering.

Figure 1: the sliding window for monitoring the Fitbit endpoint data

[38] G. Quer, P. Gouda, M. Galarnyk, E. J. Topol, and S. R. Steinhubl, "Inter- and intraindividual variability in daily resting heart rate and its associations with age, sex, sleep, BMI, and time of year: Retrospective, longitudinal cohort study of 92,457 adults," *PLOS ONE,* vol. 15, no. 2, p. e0227709, 2020.
